# A Systematic Review and Meta‐Analysis of the Role of Peripheral Inflammation in Delirium

**DOI:** 10.1002/brb3.70979

**Published:** 2025-10-20

**Authors:** Hannah C. Moorey, Lauren M. McCluskey‐White, Sahrash Andleeb, Hannah F. Botfield, Thomas A. Jackson

**Affiliations:** ^1^ School of Infection, Inflammation and Immunology University of Birmingham Birmingham UK; ^2^ Curtin Medical School Curtin University Perth Australia

**Keywords:** cytokines, delirium, immunity, inflammation, inter‐leukin‐6

## Abstract

**Introduction:**

The pathophysiology of delirium is poorly understood, but the importance of inflammation is widely accepted. The objective was to investigate whether changes in the peripheral immune system have been associated with the development of delirium in hospitalized adults.

**Methods:**

Embase and MEDLINE databases were searched. Risk of bias was assessed using the Newcastle‐Ottawa Scale. Eligible studies were split into three groups: Peripheral immune response was measured (1) preceding delirium, (2) during delirium, and (3) in both incident and prevalent delirium. Quantitative data was extracted and included in the meta‐analysis; otherwise, studies were included in the qualitative analysis. (Prospero 102931)

**Results:**

149 records were included in the qualitative synthesis and 92 in the meta‐analysis. Measured preceding delirium, there was the strongest evidence for higher neutrophil‐to‐lymphocyte ratio (NLR) (mean difference (MD) 1.00, 95% Confidence Interval (CI) 0.53, 1.48, *p* < 0.00001) in those that developed delirium compared to those that did not. During delirium there was strongest evidence for higher interleukin‐6 (IL‐6) (MD 21.29, 95% CI 11.78, 30.80, *p* < 0.00001), cortisol (MD 159.6, 95% CI 120.52, 198.68, *p* < 0.00001), and leukocyte count (MD 0.79, 95% CI 0.51–1.07, *p* < 0.000001) in delirium compared to no delirium.

**Discussion:**

These results support a role for peripheral immune response and inflammation in delirium. However, the heterogeneity of the condition was reflected in the meta‐analysis, and we should be cautious extrapolating these results to specific populations. Many studies measured the same soluble markers of inflammation, and a new era of delirium research is needed that transcends this to better understand the condition and develop future treatments.

## Graphical abstract

This review found peripheral inflammation is associated with delirium. Preceding delirium, the neutrophil‐to‐lymphocyte ratio (NLR), interleukin‐6 (IL‐6), C‐reactive protein (CRP), and Leukocyte Count(WBC) were higher, and insulin‐like growth factor‐1 (IGF‐1) lower, in those that developed delirium. During delirium, NLR, IL‐6, CRP, WBC, and cortisol were higher in delirium. Future research should move beyond broad markers of inflammation to investigate underlying mechanisms.

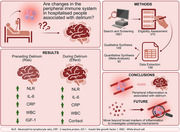

## 1 Introduction

Delirium is an acute neuropsychiatric condition resulting in changes in attention and cognition. The pathophysiology is not well understood; however, the importance of inflammation is widely accepted (Wilson et al. 2020). Common triggers of delirium include infection and surgery. Both triggers lead to a peripheral immune response, which activates immune cells and leads to the release of inflammatory mediators, which in turn activate the brain through multiple routes (Wilson et al. 2020). Delirium is particularly common in intensive care populations. The immune dysregulation seen in critical illness secondary to sepsis (Cao et al. 2023), trauma (Hazeldine et al. 2017), or surgical procedures (Angele and Faist 2002) may be an important factor in explaining this increased prevalence. We also know that with age, the risk of delirium dramatically increases (Ormseth et al. 2023) alongside changes to the immune system, termed immunosenescence (Teissier et al. 2022). A consequence of inmunosenescence is inflammaging, characterized by increased background levels of pro‐inflammatory immune mediators and a reduction in anti‐inflammatory mediators (Teissier et al. 2022). Immunesencence has also been associated with increasing frailty, which is another important risk factor for delirium (Lai et al. 2025). Changes in immune cell function with critical illness, age, and frailty may therefore help explain the pathophysiology of delirium.

Due to the technical difficulties with studying central immune responses in humans, research has predominantly compared peripheral markers of inflammation and immune cell function in people during an episode of delirium and in people without delirium. The objective of this review was to investigate whether age‐related changes in the peripheral immune system (1) preceding delirium (a marker of delirium risk) and (2) during the episode have been associated with the development of delirium in hospitalized adults. Historically reviews in the area have focused on one population, commonly postoperative populations (Mosharaf et al. 2025; Noah et al. 2021) or intensive care populations (Chan et al. 2021). We wanted to investigate if in delirium, there were common differences in the peripheral immune response throughout clinical subpopulations. Furthermore, prior reviews have focused on inflammatory biomarkers, which have little clinical utility in identifying delirium, and the extent to which they elucidate delirium pathophysiology is limited. We felt in our review it was important to include studies of peripheral immune cell phenotype and function. To date, this is the most comprehensive review on the subject, as it assesses the peripheral immune response both preceding and during delirium, includes studies of hospitalized adults from all clinical populations, and includes studies of peripheral immune cell function rather than purely biomarkers.

## 2 Methods

### 2.1 Search Strategy

The Embase and MEDLINE databases were searched in June 2018, and the search was updated in July 2022. We chose a restriction of English language papers and of 1980 (the year delirium was first included in DSM III) to the present. We also performed a manual reference search of all included papers, forward citation search using the Science Citation index and a search of research registers to identify ongoing studies. The search terms were “delirium” AND “inflammation.” For a full list of the synonyms used, please see Supplementary Material.

### 2.2 Inclusion and Exclusion Criteria

Inclusion criteria were: (1) Adults (over 18) in any patient setting (e.g., medicine, surgery, critical care) (2) Delirium prevalence or incidence is measured using Diagnostic and Statistical Manual of Mental Disorders (DSM) criteria or a tool validated against DSM criteria, excluding purely alcohol withdrawal delirium. (3) Peripheral immune system function or immune response is measured through peripheral immune cell composition or function, levels of cytokines, chemokines, or other markers of peripheral inflammation. Exclusion criteria were (1) animal studies (2) Pediatric population (all participants < 18 years). (3) Systematic or narrative reviews, case reports or case series, editorials, letters, or conference abstracts. (4) Not available in the English language. After running the search, a significant number of papers were returned that assessed neuropsychological symptoms, including delirium, in systemic lupus erythematous (SLE). These studies were not felt to be relevant to the study question, and a further exclusion criterion of (5) all patients have SLE was added.

### 2.3 Data Extraction

We imported returned search results into an Excel spreadsheet. Two authors independently reviewed titles and abstracts to assess eligibility (H.C.M. and L.L.M.). The full papers of those selected were further assessed by two authors to confirm eligibility (H.C.M. and L.L.M.). Any disagreements were discussed with a third author (T.A.J.). One author then extracted the data from the papers (H.C.M., L.L.M., T.A.J., S.A.). If levels of peripheral inflammation in a group of patients with and without delirium had been measured but the data could not be extracted, the authors were contacted by email, with a follow‐up email 2 weeks later. If no response was received, the study was either excluded or included in the qualitative synthesis alone.

### 2.4 Data Handling

Extracted data was divided into three groups: (1) studies that assessed peripheral immune response preceding delirium, (2) studies that assessed peripheral immune response during delirium, and (3) studies that documented inclusion of participants with both prevalent (present on admission) and incident (occurred during admission) delirium in the delirium group and therefore could not be allocated to either group 1 or 2. A full list of data extracted and detailed information on how quantitative data was extracted can be found in the Supplementary Material.

### 2.5 Meta‐Analysis

Meta‐analysis was performed using Review Manager Version 5.3 if there were quantitative results available for a measure of peripheral immune response from five or more studies. Whilst conducting the review, it was observed that for certain cytokines, some studies reported that levels were below the detection limit for a high percentage of participants, whilst other studies did not report this information. Furthermore, not all studies reported how undetectable cytokines were analyzed. It was therefore decided that if a study was identified that reported a high number (> 30%) of undetectable results for a certain cytokine, other studies measuring this cytokine would be excluded from meta‐analysis if there was no explanation of how undetectable cytokines were analyzed, or no description of the percentage of results below the detection limit, or if > 30% of samples were under the detection limit. Mean difference was used, and medians and interquartile ranges (IQR) were converted to mean and standard deviation (SD) using the Wan et al. method (2014). If no units were reported, a log value was reported, or no measure of range was supplied, the study was excluded from meta‐analysis. Heterogeneity was assessed using *I* ^2^. If heterogeneity was ≥ 50% a random effects model was used; otherwise, a fixed model was selected. Publication bias was assessed using funnel plot symmetry. Subgroup and sensitivity analysis was performed grouped by clinical population, whether median‐to‐mean conversion was performed, if it was confirmed that delirium was absent at baseline in studies preceding delirium, and whether delirium was present at the time of test in studies during delirium.

### 2.6 Visual Display of Data

Tables were produced listing all studies included in the qualitative and quantitative synthesis and detailing the main study characteristics and the main findings and can be found in the Supplementary Material. A visual depiction of the studies included in the qualitative review was compiled as a summary of the results. Results of the meta‐analysis were compiled in a table with an assessment of the level of certainty, and forest plots were also produced for each analysis.

### 2.7 Risk of Bias Assessment

Risk of bias was assessed using the Newcastle‐Ottawa Scale for cohort or case‐control studies by one author (H.C.M.). Studies were grouped into low risk of bias (7–9 or 7–10 for cohort studies), moderate risk of bias (4–6), and high risk of bias (0–3). Studies with a high risk of bias were not included in the meta‐analysis.

### 2.8 Assessment of Certainty

The certainty of each meta‐analysis result was assessed by considering the sensitivity analysis results, heterogeneity, risk or bias, and publication bias assessment.

## 3 Results

**FIGURE 1:**
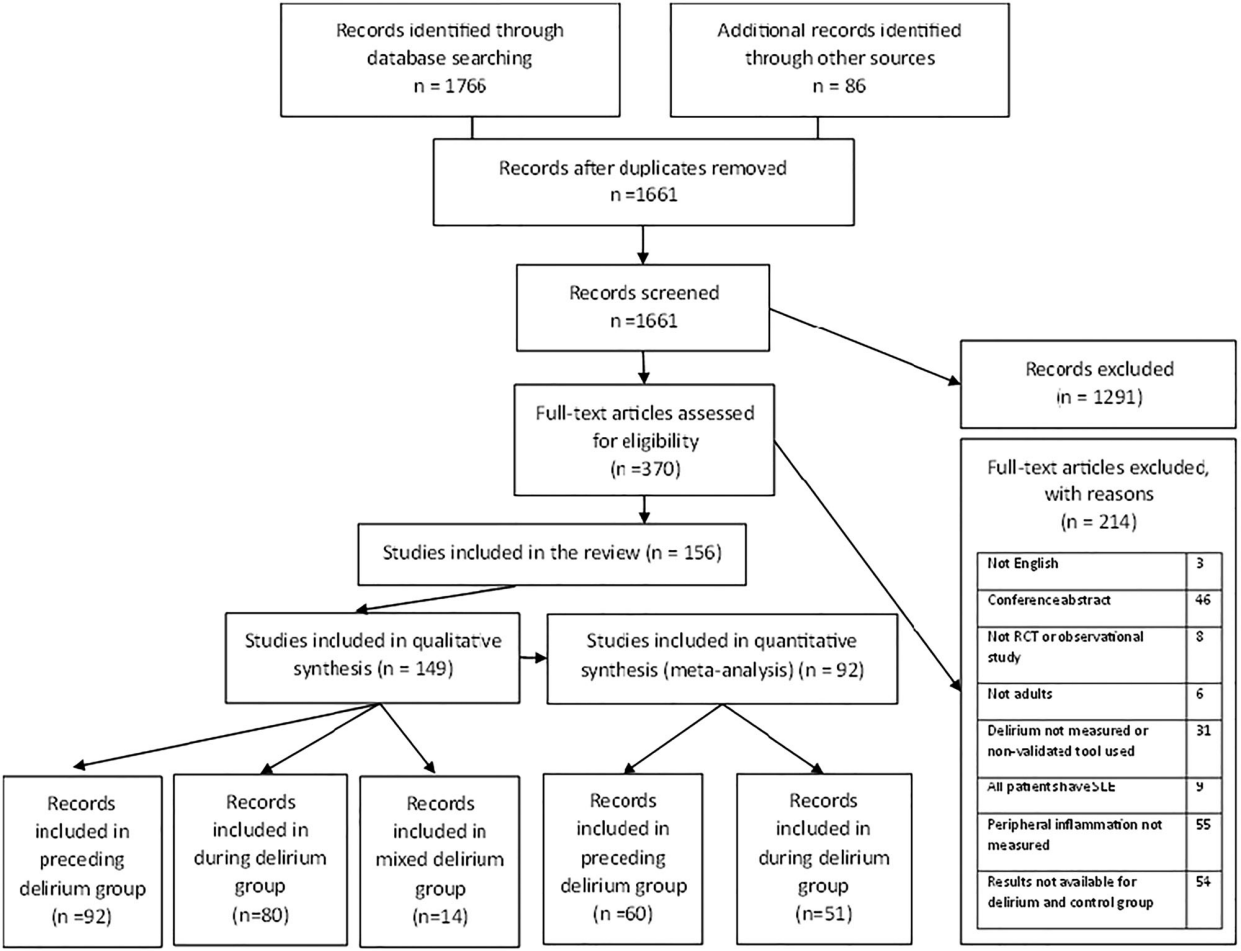
Search and selection process.

The search returned 1661 records that were assessed for eligibility, with 149 records ultimately included in the qualitative synthesis and 92 in the quantitative synthesis (meta‐analysis). Full details are seen in Figure 1.

A total of 97 different measures of peripheral inflammation were identified from the included studies (see Supplementary Material), and data were extracted.

94 records were identified that measured peripheral immune response preceding delirium, 87 during delirium, and 14 in a mixed prevalent and incident delirium population. A wide variety of clinical specialties were included, with surgical specialties being the most frequently studied (Supplementary Material). Ten diagnostic tools were used in the included studies, and the Confusion Assessment Method (CAM) or Confusion Assessment Method—Intensive Care Unit (CAM‐ICU) was the most frequently used (Supplementary Material). Of the studies that measured peripheral immune response preceding delirium, only 44/94 (46.8%) confirmed that delirium was absent at baseline. Of the studies that measured peripheral immune response during delirium, 27/88 (30.7%) did not confirm that delirium was present at the time of the test.

### 3.1 Quantitative Synthesis

92 records were included in the quantitative synthesis in 16 separate meta‐analyses. The random effects model was used in all except IGF‐1 measured preceding delirium and cortisol and leukocyte count measured during delirium. Results of the meta‐analysis are available in Table 1 with an assessment of the level of certainty.

**TABLE 1:** Summary table of meta‐analysis results.

	Outcome	Mean difference and 95% CI	Number of participants	Certainty
Preceding delirium	CRP	2.80 (1.46–4.14)	12178 (31 studies)	Lower Reduced due to heterogeneity, risk of bias and publication bias
	IL‐6	1.66 (0.53–2.79)	2169 (17 studies)	Lower Reduced due to heterogeneity, risk of bias, publication bias and sensitivity analysis
	Leukocyte count	0.52 (0.14–0.90)	9093 (20 studies)	Mod Reduced due to heterogeneity, risk of bias and sensitivity analysis
	IGF‐1	−1.47 (−2.16‐0.79)	693 (7 studies)	Mod Reduced due to sensitivity analysis.
	NLR	1 (0.53–1.48)	10336 (14 studies)	Higher
	Cortisol	41.39 (−47.1‐129.87)	483 (5 studies)	Mod Reduced due to heterogeneity
	Neutrophil count	0.78 (−0.12‐1.68)	6063 (6 studies)	Mod Reduced due to heterogeneity
	Lymphocyte count	−0.07 (−0.22‐0.08)	6331 (7 studies)	Mod Reduced due to heterogeneity
	PLR	−1.18 (−9.86‐7.51)	6165 (7 studies)	Mod Reduced due to heterogeneity
During delirium	CRP	9.92 (5.47−14.37)	9252 (30 studies)	Lower Reduced due to heterogeneity, risk of bias and sensitivity analysis
	IL‐6	21.29 (11.78−30.80)	2436 (26 studies)	Mod Reduced due to heterogeneity and risk of bias.
	Cortisol	159.6 (120.52−198.68)	1114 (9 studies)	Higher
	Leukocyte count	0.79 (0.51−1.07)	6152 (11 studies)	Higher
	NLR	1.95 (0.23−3.67)	5797 (7 studies)	Mod Reduced due to heterogeneity, and sensitivity analysis
	Neutrophil count	0.44 (−0.51‐1.38)	5334 (5 studies)	Mod Reduced due to heterogeneity
	Lymphocyte count	−0.02 (−0.23‐0.19)	5334 (5 studies)	Mod Reduced due to heterogeneity

#### 3.1.1 Measures Preceding Delirium—Delirium Risk

31 records were included in the C‐reactive protein (CRP) (Katsumi et al. 2020; Seo et al. 2021; Macdonald et al. 2007; Sakaguchi et al. 2018; Chung et al. 2015; Knaak et al. 2019; Osse et al. 2012; Vasunilashorn et al. 2017; Visser et al. 2015; Xiang et al. 2017; Zhang et al. 2022; Brattinga et al. 2022; Hindiskere et al. 2020; Khan et al. 2022; Miao et al. 2018; Shen et al. 2016; Guo et al. 2016; He et al. 2020; Lemstra et al. 2008; Neerland et al. 2016; Xu et al. 2019; Dittrich et al. 2016; Erikson et al. 2019; Jiang et al. 2020; Zhang et al. 2014; Alvarez‐Perez 2017; Guldolf et al. 2021; Kotfis et al. 2019; Lechowicz et al. 2021; Lee et al. 2011; Ren et al. 2020), 17 in the interleukin‐6 (IL‐6) (Katsumi et al. 2020; Miao et al. 2018; Shen et al. 2016; Lemstra et al. 2008; Neerland et al. 2016; Mietani et al. 2022; Peng et al. 2019; Vasunilashorn et al. 2015; Capri et al. 2014; Chen et al. 2019; Liu et al. 2013; Lv et al. 2021; Zhang et al. 2016; Ritter et al. 2014; Simons et al. 2018; Rudolph et al. 2008), 20 in the leukocyte count (Seo et al. 2021; Sakaguchi et al. 2018; Zhang et al. 2022; Hindiskere et al. 2020; Guo et al. 2016; Xu et al. 2019; Dittrich et al. 2016; Jiang et al. 2020; Alvarez‐Perez 2017; Kotfis et al. 2019; Lechowicz et al. 2021; Peng et al. 2019; Lv et al. 2021; Cerejeira et al. 2013; Chen et al. 2022; Chu et al. 2016; De Castro et al. 2014; Feng et al. 2019; Guenther et al. 2013; Shi et al. 2010), 7 in the insulin‐like growth factor‐1 (IGF‐1) (Khan et al. 2022; Miao et al. 2018; Shen et al. 2016; Lemstra et al. 2008; Cerejeira et al. 2013; Chu et al. 2016; Yen et al. 2016), 14 in the NLR (Seo et al. 2021; Zhang et al. 2022; He et al. 2020; Jiang et al. 2020; Guldolf et al. 2021; Kotfis et al. 2019; Lechowicz et al. 2021; Chen et al. 2022; Kinoshita et al. 2021; Oyama et al. 2022; Pasqui et al. 2022; Theologou et al. 2018; Yang et al. 2022; Li et al. 2022), 5 in the cortisol (Cerejeira et al. 2013; Avila‐Funes et al. 2015; Colkesen et al. 2013; Deiner et al. 2014; Kazmierski et al. 2013), 6 in the neutrophil count (Seo et al. 2021; He et al. 2020; Jiang et al. 2020; Kotfis et al. 2019; Lechowicz et al. 2021), 7 in the lymphocyte count (Seo et al. 2021; Zhang et al. 2022; He et al. 2020; Jiang et al. 2020; Guldolf et al. 2021; Kotfis et al. 2019; Lechowicz et al. 2021) and 7 in the PLR (Jiang et al. 2020; Kotfis et al. 2019; Lechowicz et al. 2021; Oyama et al. 2022; Pasqui et al. 2022; Yang et al. 2022; Li et al. 2022) meta‐analysis.

**FIGURE 2:**
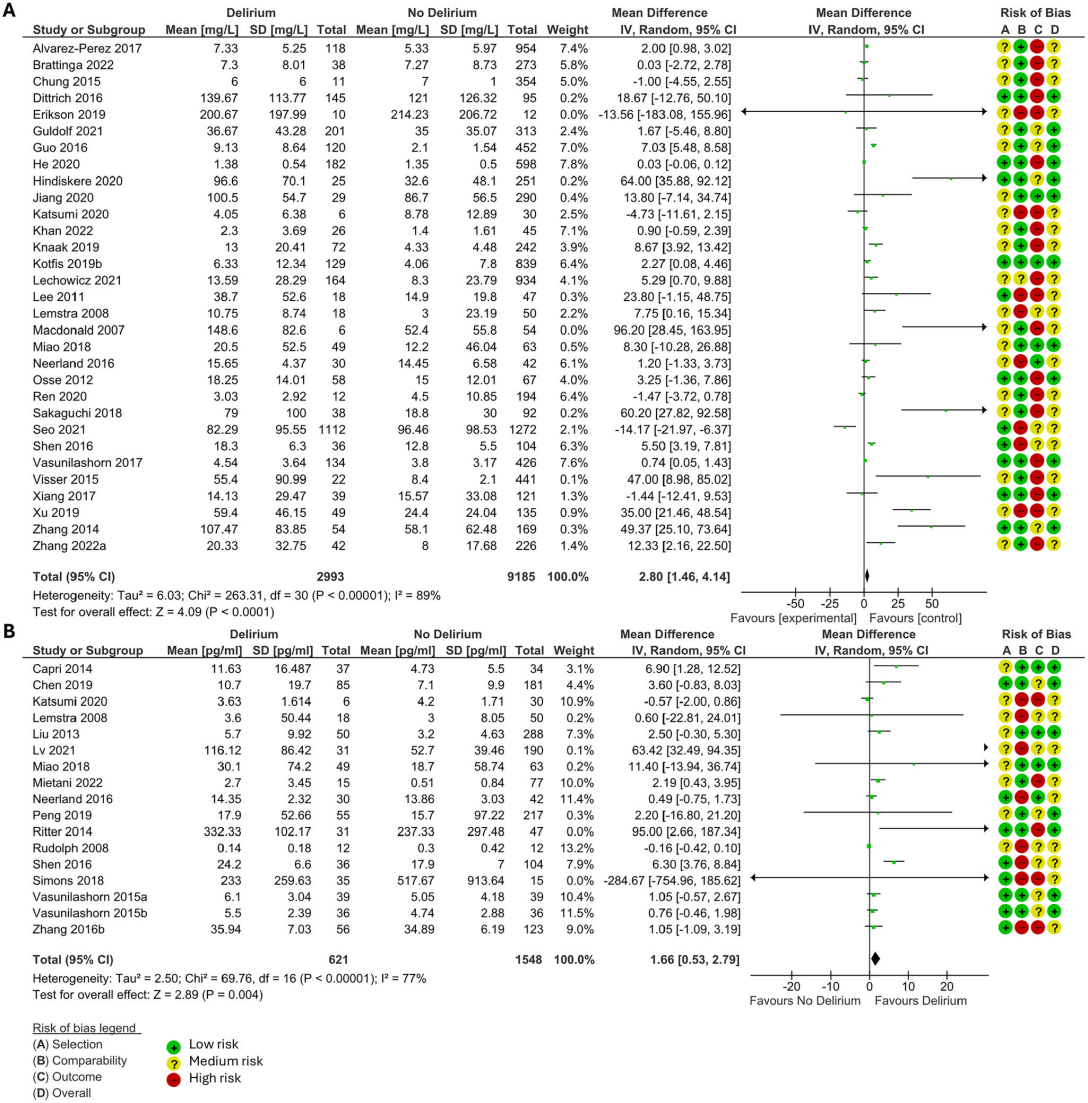
Forest plots of meta‐analysis of records measuring **(A)** CRP (mg/L) and **(B)** IL‐6 (pg/ml) preceding delirium, using a random effects model. MD and 95% CI in patients that did and did not develop delirium. The green squares represent the mean difference for each study, and the size of the square represents the weight of the study. The black lines represent the 95% CI. The black diamonds represent the overall MD.

**FIGURE 3:**
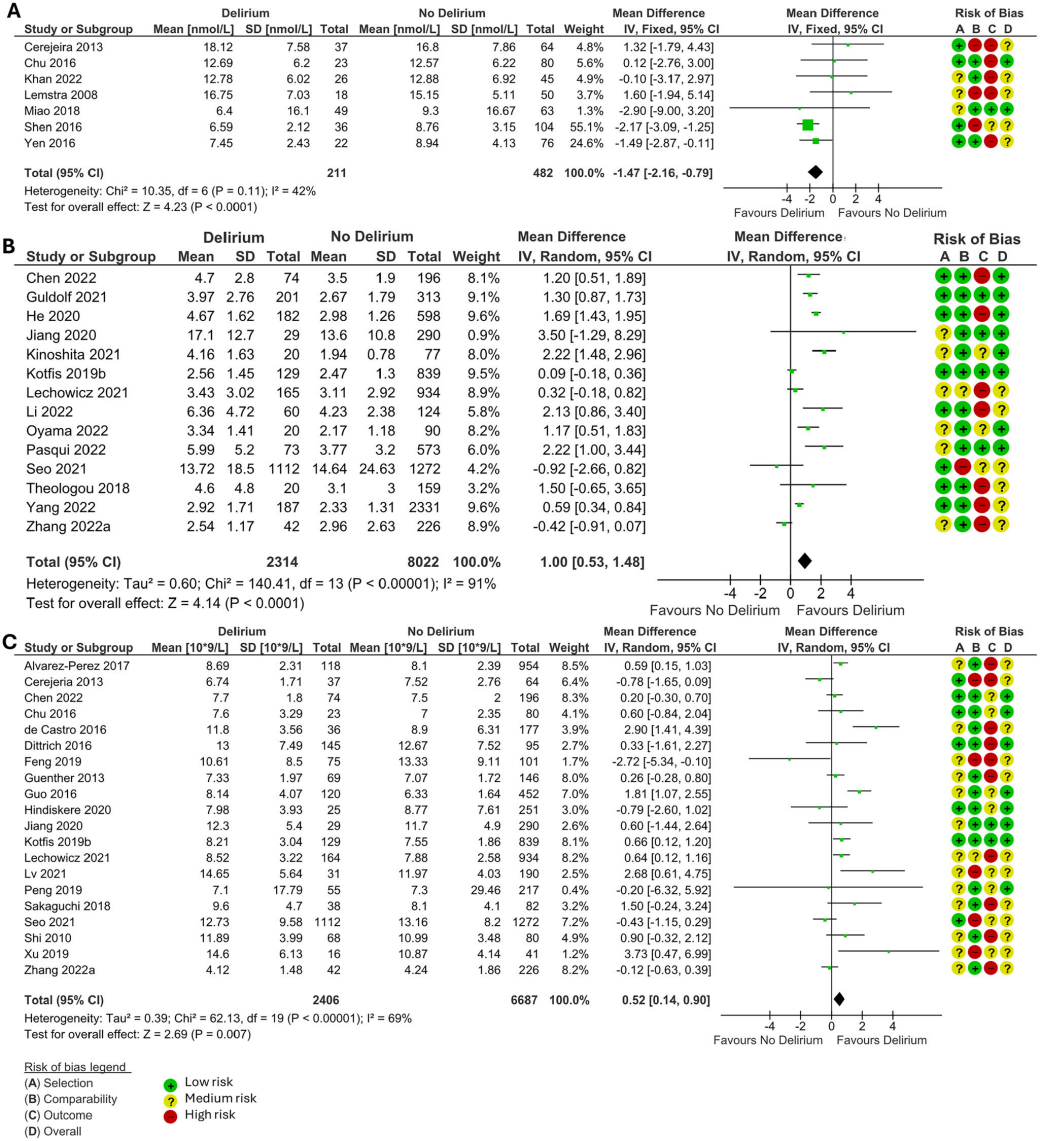
Forest plots of meta‐analysis of records measuring **(A)** IGF‐1 (nmol/L), **(B)** NLR, and **(C)** Leukocyte count (10^9^/l) preceding delirium, using a random effects model for NLR and leukocyte count and a fixed effects model for IGF‐1. MD and 95% CI in patients that did and did not develop delirium. The green squares represent the mean difference for each study, and the size of the square represents the weight of the study. The black lines represent the 95% CI. The black diamonds represent the overall MD.

When measured preceding delirium CRP (MD 2.80, 95% Confidence Interval (CI) 1.46, 4.14, p<0.0001) (Figure 2A), IL‐6 (MD 1.66, 95% CI 0.53, 2.79, *p* = 0.004) (Figure 2B), leukocyte count (MD 0.52, 95% CI 0.14, 0.9, *p* = 0.007) (Figure 3C), and NLR (MD 1.00, 95% CI 0.53, 1.48, *p* < 0.0001) (Figure 3B) were significantly higher in those that developed delirium compared to those that did not. IGF‐1 was significantly lower in those that developed delirium compared to those who did not (MD −1.47, 95% CI −2.16, −0.79, *p* < 0.0001) (Figure 3A). There was not a significant difference in cortisol, neutrophil count, lymphocyte count, or PLR in those that developed delirium and those that did not.

Following planned subgroup analysis CRP measured preceding delirium was significantly higher in delirium in studies in medicine (MD 66.89, 95% CI 37.68, 96.11, *p* < 0.00001), emergency surgery (MD 6.27, 95% CI 1.47, 11.06, *p* = 0.01), and stroke (MD 1.99, 95% CI 0.98, 3, *p* = 0.0001), but not in elective surgery, oncology, intensive care, or mixed surgery. IL‐6 measured preceding delirium was significantly higher in delirium in studies in oncology (MD 3.44, 95% CI 0.15–6.73, *p* = 0.04), but not in elective, emergency, mixed surgery, or intensive care. Leukocyte count was significantly higher in those that developed delirium in emergency surgery (MD 1.49, 95% CI 0.63, 2.34, *p* = 0.0006), mixed surgery (MD 0.91, 95% CI 0.33, 1.49, *p* = 0.002), medicine (MD 2.17, 95% CI 0.16, 4.18, *p* = 0.03) and stroke (MD 0.59, 95% CI 0.15, 1.03), but not in elective surgery, oncology, or intensive care. Sensitivity analysis confirmed CRP and NLR were still significantly higher, and IGF‐1 significantly lower, in the subgroup of studies where a mean was reported and no conversion was needed, but not IL‐6 or leukocyte count. CRP, IL‐6, and NLR were significantly higher, and IGF‐1 significantly lower, in delirium in the subgroup of studies where participants were confirmed delirium‐free at baseline, but not leukocyte count. Full results of the subgroup analysis are available in the Supplementary Material.

#### 3.1.2 Measures During Delirium—Delirium Effect

30 records were included in the CRP (Katsumi et al. 2020; Seo et al. 2021; Macdonald et al. 2007; Knaak et al. 2019; Vasunilashorn et al. 2017; Xiang et al. 2017; Khan et al. 2022; Neerland et al. 2016; Dittrich et al. 2016; Erikson et al. 2019; Kotfis et al. 2019; Lee et al. 2011; Ren et al. 2020; Zhang et al. 2016; Li et al. 2022; Page et al. 2014; Sun et al. 2016; Cizginer et al. 2017; Ma et al. 2022; Li et al. 2019). Cereghetti et al. 2017; Plaschke et al. 2010; Pol et al. 2014; Hasegawa et al. 2015; Nagase et al. 2012; de Rooij et al. 2007; Tsuruta et al. 2010; Soler‐Sanchis et al. 2022; Kotfis et al. 2019; Pfister et al. 2008; Winkelman et al. 2018), 26 in the IL‐6 (Katsumi et al. 2020; Neerland et al. 2016; Erikson et al. 2019; Mietani et al. 2022; Vasunilashorn et al. 2015; Chen et al. 2019; Liu et al. 2013; Lv et al. 2021; Zhang et al. 2016; Simons et al. 2018; de Rooij et al. 2007; Pfister et al. 2008; Winkelman et al. 2018; Jorge‐Ripper et al. 2017; Al Tmimi et al. 2015; Skrobik et al. 2013; van Munster et al. 2010; Van Munster et al. 2008; Adamis et al. 2009; Kowalska et al. 2018; Alexander et al. 2014; van den Boogaard et al. 2011; Plaschke 2013), 9 in the cortisol (Cerejeira et al. 2013; Shi et al. 2010; Kazmierski et al. 2013; Plaschke et al. 2010; Pfister et al. 2008; van Munster et al. 2010; van den Boogaard et al. 2011; Bisschop et al. 2011; Mu et al. 2010), 11 in the leukocyte count (Seo et al. 2021; Dittrich et al. 2016; Kotfis et al. 2019; Zhang et al. 2016; Plaschke et al. 2010; Plaschke et al. 2010; Nagase et al. 2012; Soler‐Sanchis et al. 2022; Kotfis et al. 2019; Kowalska et al. 2018; Shi et al. 2019; Kotfis et al. 2019), 7 in the NLR (Seo et al. 2021; Kotfis et al. 2019; Theologou et al. 2018; Li et al. 2022; Soler‐Sanchis et al. 2022; Kotfis et al. 2019; Kotfis et al. 2019), 5 in the neutrophil count (Seo et al. 2021; Kotfis et al. 2019; Soler‐Sanchis et al. 2022; Kotfis et al. 2019; Kotfis et al. 2019) and 5 in the lymphocyte count (Kotfis et al. 2019; Kotfis et al. 2019; Kotfis et al. 2019) meta‐analysis.

**FIGURE 4:**
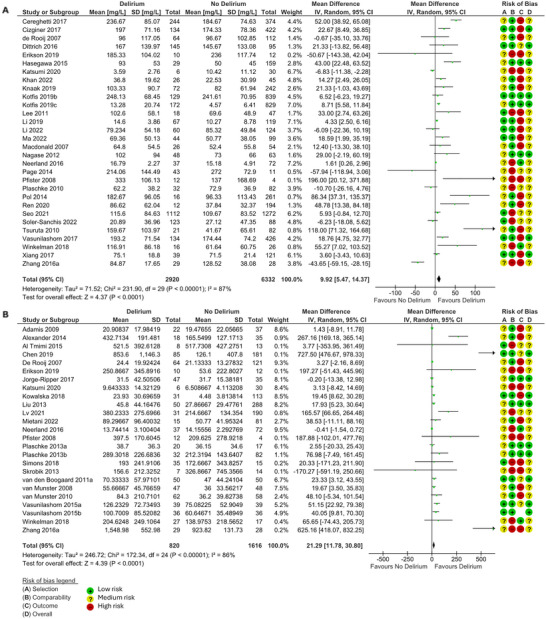
Forest plots of meta‐analysis of records measuring **(A)** CRP (mg/L) and **(B)** IL‐6 (pg/ml) during delirium, using random effects models. MD and 95% CI in patients with and without delirium. The green squares represent the mean difference for each study, and the size of the square represents the weight of the study. The black lines represent the 95% CI. The black diamonds represent the overall MD.

**FIGURE 5:**
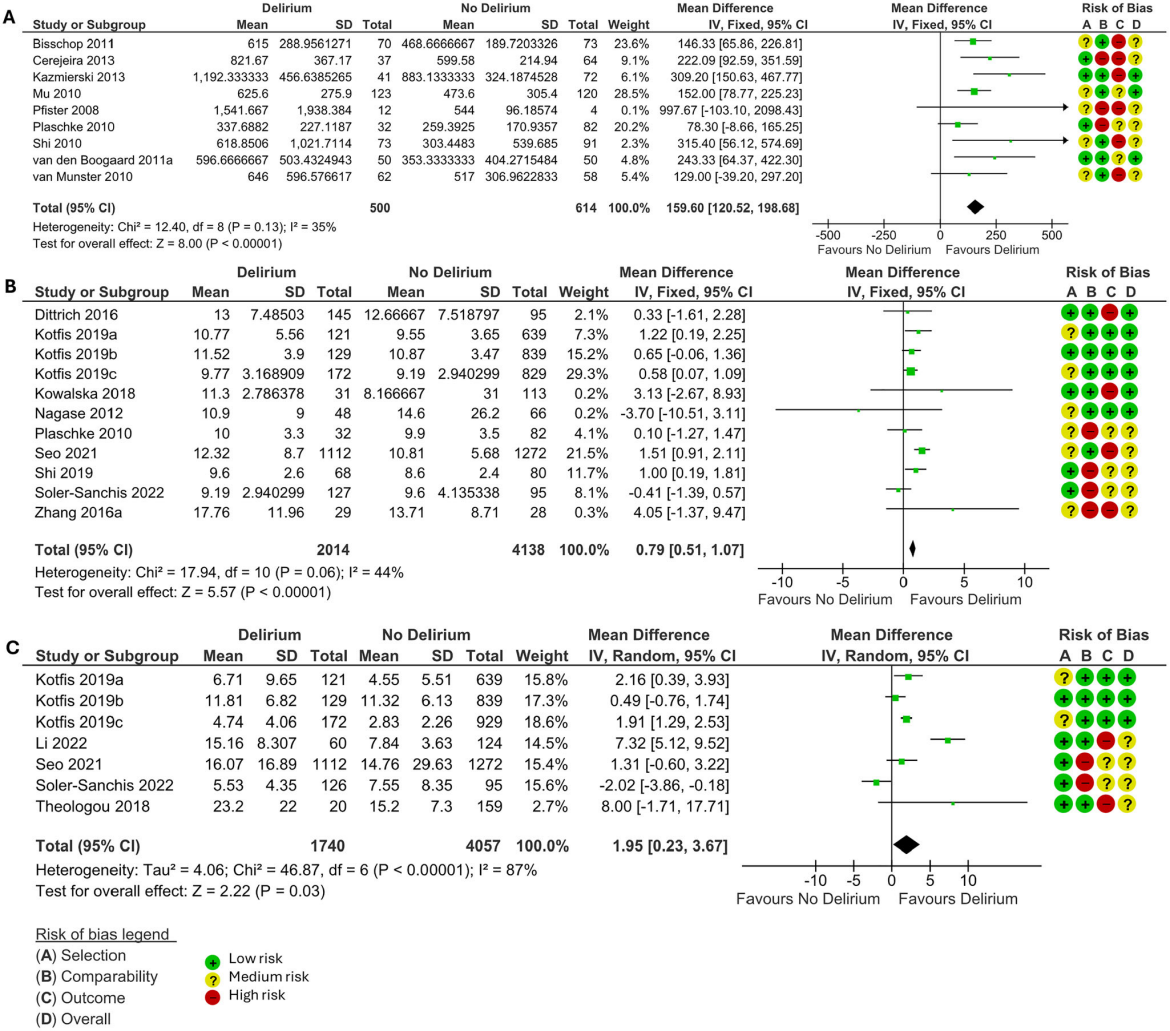
Forest plots of meta‐analysis of records measuring **(A)** Cortisol (nmol/L), **(B)** Leukocyte count (109/L), and **(C)** NLR during delirium, using a fixed effects model for Cortisol and leukocyte count and a random effects model for NLR. MD and 95% CI in patients with and without delirium. The green squares represent the mean difference for each study, and the size of the square represents the weight of the study. The black lines represent the 95% CI. The black diamonds represent the overall MD.

When measured during delirium, CRP (MD 9.92, 95% CI 5.47, 14.37, *p* < 0.0001) (Figure 4A), IL‐6 (MD 21.29, 95% CI 11.78, 30.80, *p* < 0.0001) (Figure 4B), cortisol (MD 159.6, 95% CI 120.52, 198.68, *p* < 0.00001) (Figure 5A), leukocyte count (MD 0.79, 95% CI 0.51, 1.07, *p* < 0.00001) (Figure 5B), and NLR (MD 1.95, 95% CI 0.23, 3.67, *p* = 0.03) (Figure 5C) were significantly higher in those with delirium compared to those without. There was no difference in neutrophil or lymphocyte count.

Following planned subgroup analysis CRP measured during delirium was significantly higher in delirium in studies in emergency surgery (MD 2.67, 95% CI 0.04, 5.30, *p* = 0.05), mixed surgery, (MD 38.95, 95% CI 4.7, 73.2, *p* = 0.03), oncology (MD 19.50, 95% CI 3.15, 35.86, *p* = 0.02), and stroke (MD 8.68, 95% CI 5.55, 11.81, *p* < 0.00001), but not elective surgery, medicine, or intensive care. IL‐6 was significantly higher in delirium in studies in stroke (MD 19.45, 95% CI 8.62, 30.28, *p* = 0.0004) and intensive care (MD 164.35, 95% CI 25.96, 302.73, *p* = 0.02), but not in oncology, emergency surgery, elective surgery, mixed surgery and medicine. Leukocyte count was significantly higher in delirium in studies in intensive care (MD 1.44, 95% CI 0.87, 2.01, *p* < 0.00001), stroke (MD 0.72, 95% CI 0.26, 1.18, *p* = 0.002), and mixed surgery (MD 0.71, 95% CI 0.21, 1.21, *p* = 0.005) but not in oncology or geriatric medicine. Sensitivity analysis confirmed IL‐6, cortisol, and leukocyte count were still significantly higher in delirium in the subgroup of records where mean values were reported and no conversion was needed, but CRP and NLR were not. IL‐6, cortisol, and leukocyte count were significantly higher in delirium in the subgroup where delirium was confirmed at the time of the test, but not in CRP or NLR. Full results of the subgroup analysis are available in the Supplementary Material.

### 3.2 Qualitative Synthesis

**FIGURE 6:**
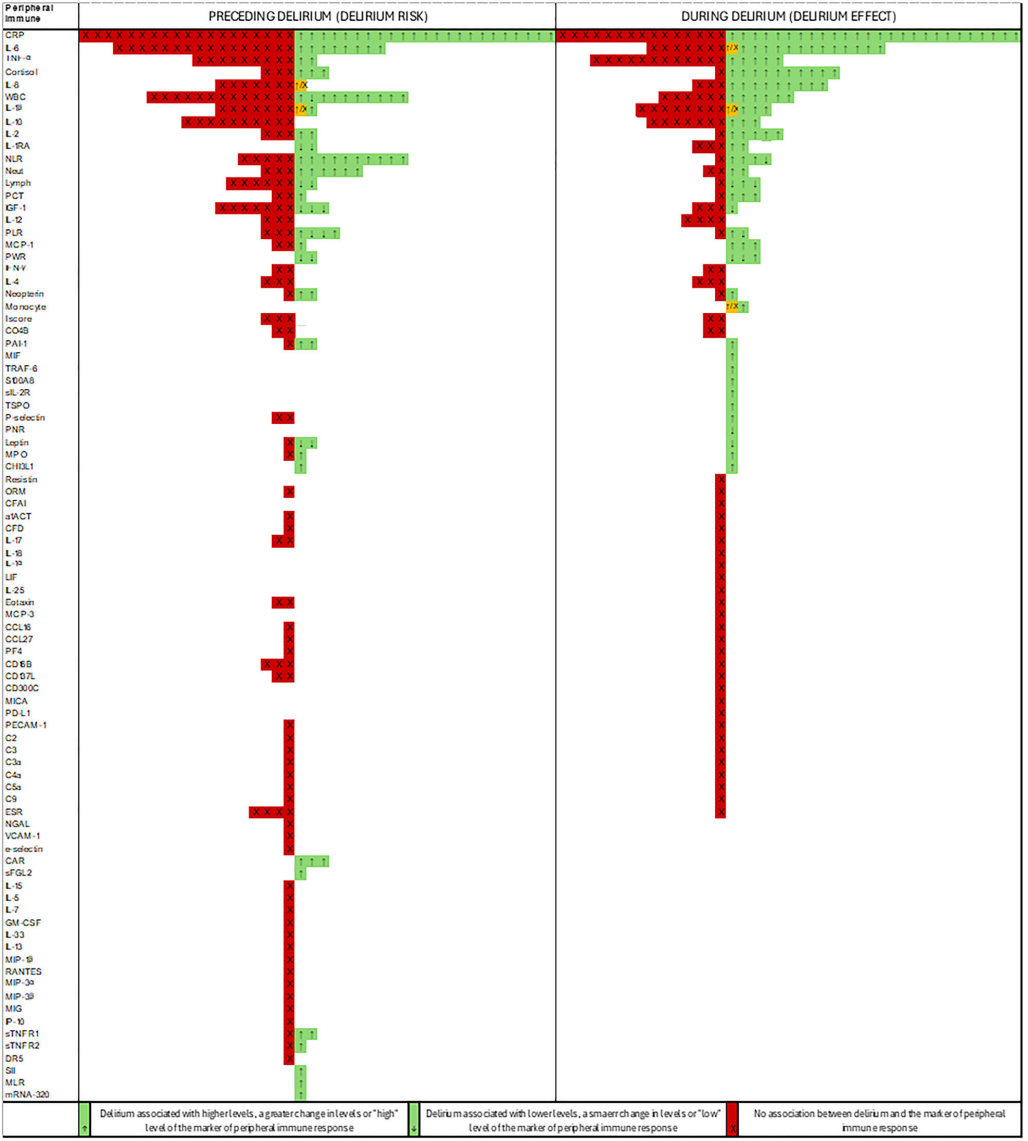
Qualitative synthesis of studies measuring inflammatory factors preceding delirium and during delirium.

156 records were included in the qualitative synthesis, and the full results can be viewed in the Supplementary Material. Figure 6 is a graphical depiction of the qualitative synthesis and reveals many records measured the most common inflammatory factors, although generally with quite contrasting results. Presented below are the results for the less commonly measured inflammatory factors that were not eligible for meta‐analysis but where a possible association with delirium was reported. The results of the records measuring immune cell function are also reported in the main text, as these results have not been reported in previous reviews.

#### 3.2.1 Acute Phase Proteins

The most commonly assessed acute phase protein was CRP, and these results have been presented in the meta‐analysis. Plasminogen activator inhibitor‐1 (PAI‐1) is a protein produced in the liver in response to pro‐inflammatory cytokines, with some evidence of an association with delirium. Two studies, reporting the same dataset, found PAI‐1 was significantly higher preoperatively in those that developed delirium compared to those that did not (Mietani et al. 2022; Mietani et al. 2022). However, one study reported no association (Girard et al. 2012). One record measured PAI‐1 during delirium and found a significantly greater rise in PAI‐1 between pre‐op and postoperative day 1 in those with delirium (Mietani et al. 2022).

#### 3.2.2 Cytokine and Chemokines

Many studies measured cytokines and chemokines, and results for IL‐6 are presented in the meta‐analysis. There was limited evidence for an association between the majority of other cytokines and chemokines included in the qualitative synthesis. However, 4/6 records that measured interleukin‐2 (IL‐2) during delirium reported significantly higher levels of IL‐2 in those with delirium compared to those without (Vasunilashorn et al. 2015; Kazmierski et al. 2013; Kazmierski et al. 2014; Kazmierski et al. 2014), and 1/6 reported a significantly greater change in IL‐2 in those with delirium compared to those without (Ballweg et al. 2021). Macrophage Migration Inhibitory Factor (MIF) was found to be significantly higher in those with delirium in one record (van den Boogaard et al. 2011). Another record reported significantly higher TNF Receptor Associated Factor 6 (TRAF‐6) and S100A8 in patients with sepsis‐associated encephalitis (Zhang et al. 2016). S100A12 was independently associated with delirium in one record (Li et al. 2019) and soluble fibrinogen‐like protein 2 (sFGL2) was an independent predictor of delirium in another (Xu et al. 2019). Monocyte chemoattractant protein (MCP‐1)/CCL2 was found to be significantly higher during delirium in one record (van den Boogaard et al. 2011). A greater change in MCP‐1 between pre‐op and postoperative day 1 in those with delirium compared to those without was reported in two records presenting results for the same dataset (Ballweg et al. 2021; Tanabe et al. 2020) and in another record that measured delirium in a mixed population (Skrede et al. 2015). However, only 1/3 of records reported higher MCP‐1 preceding delirium in those that developed delirium compared to those that did not (Rudolph et al. 2008), with 2/3 finding no association (Ballweg et al. 2021; Simons et al. 2018).

#### 3.2.3 Other Inflammatory Markers

Other inflammatory markers with a possible association with delirium include procalcitonin, a precursor to calcitonin that rises with inflammation, which was found to be significantly higher or have a significantly higher peak in those with delirium compared to those without in 3/4 records (Sun et al. 2016; van den Boogaard et al. 2011; Kupiec et al. 2020). Neopterin, a biomarker of immune system activation, was significantly higher preceding delirium in those that developed delirium compared to those that did not in 2/3 records (Osse et al. 2012; Miao et al. 2018), significantly higher during delirium in 1/2 records (Osse et al. 2012), and significantly higher in two records in a mixed population in patients that had delirium at any point compared to those that never had delirium (Egberts et al. 2015; Hall et al. 2016). Myeloperoxidase (MPO), an enzyme involved in neutrophil bacterial killing, was significantly higher preceding delirium in 1/2 records (Kaźmierski et al. 2022) and significantly higher during delirium in one record (Kaźmierski et al. 2022). CH13L1, a glycoprotein indicated in inflammation, was reported as the sole protein predictor of delirium in both pre‐operative and post‐operative models in one record (Vasunilashorn et al. 2021).

#### 3.2.4 Immune Cells

Immune cell counts, fractions, and ratios were reported in many studies and included in the meta‐analysis. However, some immune cell ratios were only reported in a few studies but provide evidence for an association with delirium. Two records measured Platelet to White Blood Cell Ratio (PWR) preceding delirium, and both reported significantly lower PWR in those that developed delirium compared to those that did not (Kotfis et al. 2019; Li et al. 2022). Four records measured PWR during delirium, and all reported lower PWR in those with delirium compared to those without (Kotfis et al. 2019; Lechowicz et al. 2021; Li et al. 2022; Kotfis et al. 2019). One study measured monocyte‐to‐lymphocyte ratio (MLR) preceding delirium and reported higher levels in those that developed delirium compared to those that did not (Yang et al. 2022). One record measured Platelet to Neutrophil Ratio (PNR) during delirium and reported that PNR was lower in the delirium group (Kotfis et al. 2019).

Immune cell function was measured in only a handful of studies. which had contrasting results. One study reported no association between NK cell activity preceding delirium and the development of delirium but did report a greater change in activity between day 1 and 2 of admission in those developing delirium compared to those without (Hatta et al. 2014). Another study, however, reported no difference in NK cell activity in those with delirium compared to those without (Nakamura et al. 2001). Cytokine production was found to be lower in monocytes from patients with delirium in one study (Kowalska et al. 2018), but two other studies reported no difference in monocyte and leukocyte cytokine production (Hatta et al. 2014; Cheheili‐Sobbi et al. 2021).

### 3.3 Risk of Bias

Most records included in the studies were of moderate (61% cohort and 60% case control) and low risk of bias (34.8% cohort and 40% case control). Risk of bias was higher in the studies included in the CRP, IL‐6, and leukocyte count meta‐analyses preceding delirium and in the CRP, IL‐6, and cortisol meta‐analyses during delirium. Full details are available in the Supplementary Material. Publication bias was assessed with funnel plots for each meta‐analysis. Funnel plots for IL‐6 and CRP measured preceding delirium had an asymmetrical appearance, suggesting high publication bias (Supplementary Material).

## 4 Discussion

Inflammation due to a peripheral immune response is a biologically plausible process in the pathophysiology of delirium, and this review confirms the presence of raised inflammation both preceding (which may be a marker of risk) and during an episode of delirium. Although there was variability in the measures of peripheral immune response studied, some soluble markers were present in the majority of studies, allowing us to complete a meta‐analysis of 16 measures. When measured preceding delirium, CRP, IL‐6, leukocyte count, and NLR were significantly higher, and IGF‐1 significantly lower, in those that developed delirium compared to those that did not. When measured during delirium, CRP, IL‐6, cortisol, leukocyte count, and NLR were significantly higher in delirium compared to no delirium. The qualitative synthesis provided additional evidence for an association between procalcitonin (PCT) and neopterin and delirium. There was also evidence for raised levels of IL‐6, IL‐2, MCP‐1, sTNFR, and sIL‐2R during an episode of delirium. This systematic review confirms the presence of changes in broad markers of inflammation before and during delirium, but these markers have limited clinical usefulness in the populations that develop delirium. The magnitude of the mean difference of these markers included in the meta‐analysis is not clinically relevant at an individual patient level. While it is convenient to measure these soluble markers, they are the product of the immune response. Few studies measured the initiation of the immune response by assessing immune cell function.

### 4.1 Comparison With Other Meta‐Analyses

There have been two previous relevant meta‐analyses of studies of peripheral immune response in delirium. One assessed fluid biomarkers during delirium in an intensive care setting (Chan et al. 2021), and the other measured biomarkers in older adults preoperatively (Noah et al. 2021). Noah et al. reported significantly higher preoperative IL‐6 in older patients who developed postoperative delirium (2021). Although we found overall higher IL‐6 was associated with developing delirium, in subgroup analysis there was no significant association in mixed, emergency, or elective surgery. This difference in results may be explained by the older age cutoff, suggesting IL‐6 may be raised at baseline in an older population in those at risk of delirium, but not in a population with a broader age range. The authors were very restrictive with the studies they included in the meta‐analysis, which may also explain the difference in results. Chan et al. reported IL‐6 was significantly higher in delirium in intensive care patients (2021). This is aligned with our findings of higher IL‐6 in delirium when measured during the episode and confirmed in our subgroup analysis that IL‐6 was higher in studies in an intensive care setting. They also reported higher interleukin‐1 receptor antagonist (IL‐1RA) however, we felt we did not have sufficient data to perform a meta‐analysis on this measure. They reported no association between CRP and delirium. Although we found an association overall, we also did not find an association in the intensive care subgroup, in keeping with their results. This review provides helpful evidence for understanding what may be important in the pathophysiology of delirium in multiple clinical populations, but we should be cautious about extrapolating results to specific populations.

### 4.2 Limitations of the Studies Reviewed

We identified two key limitation themes in the studies included in our review. First, there were concerns around delirium assessment and diagnosis. Assessments were not always performed at the optimum time. Where peripheral immune response was measured preceding delirium, researchers did not always confirm that delirium was absent. Similarly, in studies where peripheral immune response was measured during delirium, researchers did not always confirm that the participants had delirium at the time of the test. We have tried to reduce the impact of this in the meta‐analysis through subgroup analysis. Results suggest we should be more certain that CRP, leukocyte count, and NLR are associated with delirium when measured preceding, and IL‐6, cortisol, and leukocyte count are associated with delirium during. Furthermore, follow‐up was not always long enough to identify delirium. A significant number of studies followed up patients for less than three days following a trigger, and this may have led to some participants being incorrectly classified. Most studies did not provide information on whether there was missing data in the follow‐up delirium screening or if researchers screening participants were blinded to laboratory results. Finally, few studies considered if the delirium phenotype influenced the results, so we were unable to collect this information.

Second, there were specific limitations with studies that investigated inflammatory cytokines. Cytokines are often below the detection limit for the assay, especially when measured at baseline prior to an inflammatory trigger. It is important to specify how these values are dealt with and what percentage of samples had undetectable levels. We address this in the meta‐analysis by choosing not to include studies that did not provide this information if any other studies had reported a high percentage of undetectable levels. There were also limitations with data needing to be excluded from the meta‐analysis because units were not reported, were incorrect, or sufficient data was not available to allow meta‐analysis.

### 4.3 Limitations of this Review

Delirium is by nature a heterogenous condition, with a variety of and sometimes multiple triggers. This inevitably leads to high heterogeneity in any meta‐analyses. However, it allows us to draw broad conclusions about delirium. We used the random effects model in most of the meta‐analyses due to recognized high heterogeneity and results were mainly in keeping with reviews in more specific populations. Data reported in studies was often non‐parametric. and medians were reported. These were converted to means for the meta‐analysis using a commonly used method; however, this data is less reliable. Subgroup analysis was performed to confirm if inclusion of this data changed the results. We can be more certain that CRP, IGF‐1, and NLR preceding, and IL‐6, cortisol, and leukocyte count during, are associated with delirium. The Newcastle‐Ottawa Scale was used to assess risk of bias, and most studies had low or moderate risk of bias. A limitation of this assessment method was that retrospective studies tended to score higher than prospective studies. We know that delirium is often under‐recognized, and studies that retrospectively check records for a diagnosis may be more inaccurate but be classified as lower risk of bias. Furthermore, this assessment did not work well for data included from randomized controlled trials. A strength of this review is that it is extensive, and effort was made to ensure all available data was included. However, it is difficult to account for publication bias. The meta‐analyses of CRP and IL‐6 preceding delirium appear to be at high risk of publication bias, which should be considered when drawing conclusions. Finally, this review focused on measures of peripheral inflammation; however, delirium is a disease of the brain, and the extent to which changes in the periphery reflect the central nervous system is unknown.

### 4.4 Implications of Future Research

There are now almost 50 studies that measure IL‐6 in delirium, demonstrating marginally higher levels. Ten studies have measured interleukin‐10 (IL‐10), with no association found with delirium in any of them. The failure of this methodology to produce clinically relevant results indicated it is time to move past studies measuring the same soluble markers of inflammation. Future research needs to rectify the common methodological issues to provide usable results that will bring us closer to understanding delirium. One approach is to investigate new inflammatory markers that have been implicated in recent studies. For example, tumor necrosis factor receptor‐associated factor 6 (TRAF6), the calcium‐binding proteins S100A8, and S100A12, soluble fibrinogen‐like protein 2 (sFGL2), and the cytokine macrophage migration inhibitory factor (MIF). A more ambitious and innovative approach would be to go beyond markers that represent the end of the inflammation process at a single timepoint and focus on the initiation of the immune response and the consequent cellular response. This could initiate a new era of delirium research.

## 5 Conclusion

Age‐associated changes to the peripheral immune response and inflammation play a role in delirium. When measured preceding delirium, and hence a measure of delirium risk, there was evidence for significantly higher NLR, CRP, IL‐6, and leukocyte count and lower IGF‐1 in those that developed delirium compared to those that did not. During delirium there was evidence for significantly higher IL‐6, cortisol, leukocyte count, CRP, and NLR in delirium compared to no delirium. A new era of delirium research is needed that goes beyond measuring soluble markers of inflammation to underlying biological mechanisms if we are to better understand the condition and develop future treatments.

## Author Contributions

H.C.M. and T.A.J. were involved in the conception or design of the work; H.C.M., T.A.J., L.M.M.W., S.A. were involved in data acquisition. H.C.M. and T.A.J. performed the data analysis and interpretation of data for the work. All authors were involved in the drafting the work or reviewing it critically for important intellectual content and final approval of the version to be published.

## Ethics Statement

Ethical approval was not required for this systematic review and meta‐analysis.

## Conflicts of Interest

The authors declare no conflicts of interest.

## Registration and Protocol

The review was registered with PROSPERO where the protocol can be found (https://www.crd.york.ac.uk/PROSPERO/display_record.php?RecordID=102931).

## Peer Review

The peer review history for this article is available at https://publons.com/publon/10.1002/brb3.70979.

## Supporting information




**Supplementary Material**: brb370979‐sup‐0001‐SuppMat.docx

## Data Availability

The data that support the findings of this study are available from the corresponding author upon reasonable request.
