# Supplementary material for: A Systematic Review and Meta‐Analysis of the Role of Peripheral Inflammation in Delirium
Source: Brain Behav. 2025 Oct 20;15(10):e70979. doi: 10.1002/brb3.70979 (PMC12537839; doi:10.1002/brb3.70979)
Supplement: Supplementary file 1 — Supplementary Material: brb370979‐sup‐0001‐SuppMat.docx [file BRB3-15-e70979-s001.docx]

Supplementary Material

Table 1 Full List of Search Terms included in the Systematic Review

| Delirium (MeSH/Keyword) | OR | Acute confusional state (Keyword) |
| --- | --- | --- |
| Sepsis associated encephalopathy (MeSH) | OR | Delirium (as keyword) |
| Acute confusion (MeSH) | | |
| AND | | |
| Inflammation (MeSH) | OR | Immune system (MeSH) |
| Immunology (MeSH/Keyword) | OR | Immune response (MeSH/Keyword) |
| Immune (Keyword) | OR | Neuroinflammation (Keyword) |
| Nervous system inflammation (MeSH/Keyword) | OR | Monocytes (MeSH) |
| Macrophages (MeSH) | OR | Neutrophils (MeSH) |
| Natural Killer Cells (MeSH) | OR | T lymphocytes (MeSH) |
| B lymphocytes (MeSH) | OR | Cytokines (MeSH) |
| Chemokines (MeSH) | OR | Interleukins /Interleukin derivatives (MeSH) |
| Interleukin-1 (MeSH) | OR | Interleukin-1beta (MeSH) |
| Interleukin-1alpha (MeSH) | OR | Interleukin-1recepetor blocking agent (MeSH) |
| Interleukin-2 (MeSH) | OR | Interleukin-3 (MeSH) |
| Interleukin-4 (MeSH) | OR | Interleukin-6 (MeSH) |
| Interleukin-8 (MeSH) | OR | Interleukin-10 (MeSH) |
| Interleukin-12 (MeSH) | OR | Interleukin-18 (MeSH) |
| Monocyte chemotactic protein 1 (MeSH/Keyword) | OR | Chemokine CCL2 (MeSH/Keyword) |
| Tumour necrosis factor alpha (MeSH) | OR | Interferon gamma (MeSH) |
| C reactive protein (MeSH) | OR | Brain derived neurotrophic factor (MeSH) |
| Leukocytes (MeSH) | OR | Cortisol (Keyword) |
| Insulin-like growth factor 1 (MeSH/Keyword) | | |

## Full List of Data Extracted from Records

- Author
- Year
- Aims of study
- Study design – e.g. cohort study, RCT
- Primary and Secondary outcomes
- Country
- Sample Size
- Justification for sample size
- Age- average in total sample, delirium and no delirium
- Patient population
- Inclusion criteria
- Exclusion criteria
- Dementia - if recorded and level in total sample, delirium and no delirium
- Sex - if recorded and level in total sample, delirium and no delirium
- Comorbidities - if recorded, how measured and level in total sample, delirium and no delirium
- Illness severity - if recorded, how measured and level in total sample, delirium and no delirium
- Frailty – if recorded, how measured and level in total sample, delirium and no delirium
- Other confounding factors
- Mortality – if recorder, over what duration and rate
- Delirium diagnosis tool
- Delirium prevalence – if recorded and rate
- Delirium incidence- if recorded and rate
- Delirium severity – if recorded and scale
- Sample peripheral immune function measured in e.g. blood, saliva etc.
- Timepoint peripheral immune function measured and if samples taken at same time each day
- How undetectable cytokines were dealt with
- Duration of participation
- Location of recruitment
- Informed consent
- Withdrawals and exclusions and missing data
- Ethical approval
- Bias identified by researchers or reviewers
- Funding source specified
- Conflicts of interest

## Supplementary Quantitative Data Extraction Methods

When extracting quantitative data from studies with multiple time points the following rules were observed. For studies measuring immune response preceding delirium the result that was prior to, and closest to, the delirium trigger was selected. For studies measuring immune response during delirium the datapoint was selected where participants could definitely be defined as having delirium. If there was more than one time point that met this criterion, the timepoint with the highest level of inflammation in the delirium group was selected. In many of the studies of surgical populations it could not be confirmed that participants definitely had delirium at each time point, only that they had delirium at some point post-operatively. In this situation if the study reported when the highest incidence of delirium diagnosis was the corresponding time point was selected. If this was not reported the timepoint with the highest level of inflammation in the delirium group was selected. If quantitative data was not available in the text or a table but was presented in a graph an open-source online software, WebPlotDigitizer, was used to extract the data. Data was extracted three times and an average taken to improve accuracy.

Table 2 Table of Measure of Peripheral Inflammation included in the Review and the Number of Records that Measured each Factor

| Inflammatory Factor | Class | Description | No. Records |
| --- | --- | --- | --- |
| C-reactive protein (CRP) | **Acute Phase Proteins** | Pentameric protein synthesized by the liver which rises in response to inflammation. | 79 |
| CRP/Albumin Ratio (CAR) |  | Calculated measure of inflammation. | 3 |
| Plasminogen activator inhibitor-1 (PAI-1) |  | Member of the serine superfamily and regulates the fibrinolytic system by inhibiting plasminogen activators. Levels rise in response to a pro-inflammatory state. | 2 |
| Alpha-1-acid glycoprotein/ Orosomucoid (ORM) |  | Glycoprotein that acts as a primary carrier of basic and neutrally charged lipophilic compounds. Acute phase protein. | 1 |
| Alpha-1-antichymotrypsin |  | Protease inhibitor that protects tissue from damage from neutrophils. Acute phase protein. | 1 |
| Complement factor I (CFAI) |  | Regulates complement activation by cleaving C3b and C4b. Has been implicated in age-related macular degeneration. | 1 |
| Complement factor D |  | Involved in the alternative pathway of the complement system and cleaves factor B. Low levels associated with obesity. | 1 |
| Interleukin-6 (IL-6) | **Cytokines** | Important pro-inflammatory cytokine produced by macrophages that activates acute phase proteins, stimulates the production of neutrophils and supports the growth of b cells. | 47 |
| Tumour Necrosis Factor alpha (TNF-α) |  | Secreted by macrophages and other cells. Potent chemoattractant for neutrophils and stimulates IL-1 production in macrophages. | 26 |
| Interleukin-1β (IL-1β) |  | Secreted by activated macrophages, monocytes and microglia. Mediates inflammatory response and is involved in cell proliferation, differentiation and apoptosis. | 23 |
| Interleukin-10 (IL-10) |  | Anti-inflammatory cytokine that inhibits the activity of TH1 cells, NK cells and macrophages. | 21 |
| Interleukin-2 (IL-2) |  | Secreted by CD4+ and CD8+ T cells and promotes T cell differentiation and enhances the killing abilities of NK cells and CD8+ T cells. | 9 |
| Interleukin-1 receptor antagonist (IL-1RA) |  | Binds to the IL-1 receptor and prevents other IL-1 cytokines binding and therefore blocks their pro-inflammatory effects. | 8 |
| Interleukin-12 (IL-12) |  | Produced by a number of immune cells and stimulates production of IFN-γ and TNF-α. | 6 |
| Interferon Gamma (IFN-γ) |  | Produced by NK cells and CD8+ and CD4+ T cells. Activates macrophages and has a direct effect on viral replication. | 6 |
| Interleukin-4 (IL-4) |  | Produced by TH2 cells, mast cells, eosinophils and basophils. Has a number of functions including inducing differentiation pf T cells to TH2 cells and B cells to plasma cells and class switching of b cells to IgE. | 4 |
| Interleukin-17 (IL-17) |  | Produced by TH22 cells and leads to the production of cytokines and chemokines and immune cell recruitment. | 4 |
| Interleukin-5 (IL-5) |  | Produced by TH2 cells and mast cells and stimulates b cell growth and increases immunoglobulin secretion. | 3 |
| Interleukin-15 (IL-15) |  | Similar to IL-2. Secreted by mononuclear phagocytes following viral infection and induces NK cell proliferation. | 3 |
| Interleukin-18 (IL-18) |  | Produced by macrophages, along with other cells, and acts together with IL-12 to induce IFN-γ production. | 3 |
| Inflammatory score |  | The sum of the pro-inflammatory cytokines (IL-2 + IL-6 + IL-8) divided by anti-inflammatory cytokine (IL-10) | 3 |
| Interleukin-1α (IL-1α) |  | Member of the IL-1 family that is important in the initiation of inflammation. | 2 |
| Interleukin-7 (IL-7) |  | Produced by a number of non-immune cells and important in B and T cell development. | 1 |
| Interleukin-13 (IL-13) |  | Similar role to Il-4 and important in the regulation of IgE and a mediator of allergic inflammation. | 2 |
| Leukaemia Inhibitory Factor (LIF) |  | IL-6 class cytokine. Inhibits cell differentiation. | 2 |
| Granulocyte-macrophage cell stimulating factor (GM-CSF) |  | Cytokine that promotes the proliferation and maturation of granulocytes and monocytes. | 2 |
| Neutrophil gelatinase-associated lipocalin (NGAL) |  | Adipose derived cytokine that modulates inflammation. | 2 |
| Interleukin-25 (IL-25) |  | Produced by many cell types. Stimulates IL-8, IL-4, IL-5 and IL-13 production and decreases in IFN-ɣ. Promotes TH2 immune response. | 1 |
| Interleukin-33 (IL-33) |  | Member of the IL-1 superfamily. Produced by many cells and drives production of Th2 associated cytokines. | 1 |
| Macrophage migration inhibitory factor (MIF) |  | Regulates macrophages through suppressing the anti-inflammatory effects of glucocorticoids. | 1 |
| TNF Receptor Associated factor (TRAF 6) |  | Family of proteins involved in the regulation of inflammation. | 1 |
| S100 calcium binding protein A12 (S100A12) |  | Calcium binding protein that stimulates innate immune cells to produce proinflammatory cytokines and up-regulates cell adhesion molecules. Acts as a monocyte and mast cell chemoattractant. | 1 |
| S100calcium binding protein A8 (S100A8) |  | Calcium binding protein that is expressed on monocytes and neutrophils. Stimulate leucocyte recruitment and cytokine secretion. | 1 |
| Soluble fibrinogen like protein (sFGL2) |  | Secreted by regulatory T cells and represses the differentiation and proliferation of T cells and the maturation of dendritic cells as well as other immunosuppressive activities. | 1 |
| Immune Profiles |  | Cytokines grouped to form different immune profiles. | 1 |
| Interleukin-8 (IL-8/CXCL8) | **Chemokines** | Produced by macrophages and leads to neutrophil and other granulocyte migration and phagocytosis. | 19 |
| Monocyte chemoattractant protein -1 (MCP-1/CCL-2) |  | Recruits monocytes, memory T cells and dendritic cells to sites of inflammation. | 6 |
| Eotaxin (CCL11, CCL24, CCL26) |  | A cc chemokine subfamily involved in eosinophil chemotaxis. | 2 |
| Macrophage inflammatory protein-1β (MIP-1β /CCL4) |  | Involved in the recruitment and activation of cells expressing CCR2 or CCR5 including granulocytes, t lymphocytes and NK cells. . | 1 |
| RANTES/CCL5 |  | Mainly expressed by t-cells and monocytes and acts as a chemoattractant for monocytes, memory T helper cells and eosinophils. | 1 |
| Monocyte chemoattractant protein-3 (MCP-3/CCL7) |  | Closely related to CCL2. Expressed in many types of cells. Mainly a chemoattractant to monocytes but also other leucocytes. | 1 |
| CCL16 |  | Expressed in the liver, thymus and spleen and acts as a chemoattractant to monocytes and lymphocytes. | 1 |
| Macrophage inflammatory protein-3β (CCL19/MIP-3β) |  | Expressed mainly in the thymus and lymph nodes. Chemoattractant to dendritic cells, B cells and memory T cells. | 1 |
| Macrophage inflammatory protein- 3(CCL20/MIP-3) |  | Strong chemoattractant to lymphocytes and weak chemoattractant to neutrophils. | 1 |
| CCL27 |  | Chemokine for skin-associated memory T lymphocytes. | 1 |
| Platelet Factor 4 (PF4)/CXCL4 |  | Released from activated platelets. Involved in coagulation and is a strong chemoattractant for neutrophils and monocytes. | 1 |
| Monokine induced gamma interferon (MIG/CXCL9) |  | Released by several cells in response to IFN-γ. Particularly important in the chemoattraction of T-lymphocytes of the TH-1 phenotype. | 1 |
| Interferon gamma-induced protein 10 (IP-10/CXCL10) |  | Released by several cells in response to IFN-γ. Important in chemoattraction of monocytes, macrophages, T cells, NK cells and dendritic cells and promotes adhesion of T cells. | 1 |
| Soluble tumour necrosis factor receptor-1 (sTNF-R1) | **Cell surface markers and receptors** | Receptor for TNF that is expressed by many cells. Binding leads to pro-inflammatory effects. | 3 |
| Soluble interleukin-2 receptor (sIL-2R) |  | Receptor that IL-2 binds to. | 2 |
| Soluble tumour necrosis factor receptor-2 (sTNF-R2) |  | Receptor for TNF that is expressed by endothelial and immune cells. Binding leads to anti-inflammatory effects. | 2 |
| Soluble interleukin-6 receptor (sIL-6R) |  | Receptor that IL-6 binds to. | 1 |
| Death receptor 5 (DR5) |  | Cell surface receptor that binds TRAIL and is involved in apoptosis. | 1 |
| Low affinity immunoglobulin gamma Fc region receptor III-B (CD16B) |  | Receptor that may act as an immune complex trap by binding aggregated IgG. | 1 |
| Tumour necrosis factor ligand superfamily member 9 (CD137L) |  | Transmembrane glycoprotein that binds 401BB on activated T cells. | 1 |
| Translocator Protein (TSPO) |  | Protein mainly found on the outer mitochondrial membrane. Modulates oxidative burst in neutrophils and macrophages and inhibits cytokine secretion by macrophages. | 1 |
| CMRF35-like molecule 6 (CD300C) |  | Antigen that is present on Monocytes, neutrophils and some T and B lymphocytes. | 1 |
| MHC class I polypeptide-related sequence A (MICA) |  | Cell surface glycoprotein that acts as a stress induced self-antigen and activates the effector cytolytic responses of T cells and NK cells. | 1 |
| Programmed cell death 1 ligand 1 (PD-L1) |  | Transmembrane protein that binds to receptor PD-1 on T cells and inhibits IL-2 production and T cell proliferation. | 1 |
| P-selectin | **Cell adhesion molecules** | Expressed by activated endothelial cells and platelets. Imported in the initial recruitment of leucocytes to the site of inflammation. | 2 |
| PECAM-1 |  | Expressed by hematopoietic and endothelial cells and facilitate leucocyte transendothelial migration. | 2 |
| E-selectin |  | Expressed by endothelial cells in response to cytokines. Important in recruiting leucocytes to the site of injury | 1 |
| VCAM-1 |  | Protein that mediates the adhesion of immune cells to the vascular endothelium. | 1 |
| Leucocyte | **Immune Cells** | Cells of the immune system that are produced in the bone marrow and protect against infection. Representend as a count or cell activity measured through cytokine production. | 42 |
| Neutrophil to lymphocyte ratio (NLR) |  | Neutrophil count divided by the lymphocyte count | 23 |
| Neutrophil |  | Most abundant granulocyte that are phagocytes with an essential role in the innate immune system. Represented as a count or percentage of leucocytes. | 14 |
| Lymphocyte |  | Type of leucocyte that are the major component of the adaptive immune system. Include T cells, B cells and NK cells. Represented as a count or percentage of leucocytes. | 14 |
| Platelet to lymphocyte ratio(PLR) |  | Platelet count divided by the lymphocyte count | 8 |
| Monocyte |  | Type of mononuclear leucocyte found in the peripheral circulation. Differentiate into macrophages in the tissues. Act as phagocytes and are important antigen presentation and cytokine production. Represented as a count, percentage of leucocytes or cell activity measured through cytokine production. | 4 |
| Platelet to white blood cell ratio (PWR) |  | Platelet count divided by the white blood cell count | 4 |
| Natural Killer (NK) Cells |  | Cytotoxic lymphocyte that are able to target cells not identified by the MHC system. Represented as a count or cell activity. | 2 |
| Systemic Immune-Inflammation Index (SII) |  | NLR multiplied by the platelet count. | 2 |
| Basophil |  | Granulocyte that are important in the immune response to parasites and allergy. | 1 |
| Monocyte to lymphocyte ratio (MLR) |  | Monocyte count divided by the lymphocyte count | 1 |
| Platelet to neutrophil ratio (PNR) |  | Platelet count divider by the neutrophil count. | 1 |
| Complement C4-B (CO4B) | **Complement** | Cleaved from C4. Participates in all 3 complement pathways. Has been implicated in schizophrenia risk. | 2 |
| Complement component C2 |  | Part of the classical pathway of the complement system. Has been associated with autoimmune disease and immunodeficiency. | 1 |
| Complement component C3 |  | Plays a central role in activating the classical and alternative complement activation pathways. Associated with autoimmune disease and immunodeficiency. | 1 |
| Complement component C3a |  | Protein formed by the cleavage of C3. Activates T cells and macrophages, induces chemotaxis and mast cell degranulation. | 1 |
| Complement component C4-A |  | Cleaved from C4. Participates in all 3 complement pathways. Has been implicated in schizophrenia risk. | 1 |
| Complement component C5a |  | Protein formed by the cleavage of C5 that is highly inflammatory encouraging complement activation, formation of the membrane attack complex and chemotaxis. | 1 |
| Complement component C9 |  | Member of the complement membrane attack complex which results in lysis and cell death. Under expression has been associated with longevity. | 1 |
| Cortisol | **Hormones** | The main glucocorticoid hormone which has a number of functions including anti-inflammatory action on the immune response. | 17 |
| Insulin-like growth factor 1 (IGF-1) |  | Hormone with a similar molecular structure to insulin which has anabolic effects and bidirectional interactions with the immune system. | 15 |
| Leptin |  | Hormone that helps to regulate energy balance levels rise in response to adipose derived-inflammatory cytokines. | 3 |
| Resistin |  | Peptide hormone that increases pre-inflammatory cytokines and upregulates intercellular adhesion molecules. | 1 |
| Procalcitonin (PCT) | **Other Inflammatory Markers** | Peptide precursor of the hormone calcitonin which rises in response to inflammation. | 5 |
| Erythrocyte sedimentation rate (ESR) |  | The rate at which red blood cells descend over one hour. Rises with inflammation. | 5 |
| Neopterin |  | Belongs to the chemical group pteridines and is produced by macrophages after stimulation by IFN-γ. Considered a biomarker for immune system activation. | 5 |
| Myeloperoxidase (MPO) |  | Enzyme, mainly produced by neutrophils to produced reactive oxygen species (ROS) to kill microbes. Serum levels of MPO have also been suggested as biomarker of inflammation. | 2 |
| Histidine-rish Glycoprotein (HRG) |  | Glycoprotein produced by the liver and some immune cells. Involved in clearance of apoptotic phagocytes, immune complexes, cell adhesion and migration. | 1 |
| Chitinase-3-like protein 1 (CHI3L1) |  | Secreted glycoprotein that catalyses the hydrolysis of chitin. Implicated in inflammation and levels elevated in Alzheimer’s disease. | 1 |
| MicroRNA-320 |  | Non-coding, single-stranded molecules that regulate target genes. Inhibits IGF-1. | 1 |

## Summary Tables of Studies Included in Quantitative Synthesis

Table 3 Records included in the Quantitative Synthesis that measured peripheral immune response preceding delirium

| **Author and Date** | **Setting** | **Number with Delirium/**  **Sample Size** | **Diagnostic Criteria** | **Confirmed delirium free at time of test** | **Measure of Peripheral Inflammatory Response included in Meta-analysis** |
| --- | --- | --- | --- | --- | --- |
| Alvarez-Perez, F. J. and F. Paiva, 2017(Alvarez-Perez F.J., 2017) | Stroke, Portugal | 118/1072 | DSM 5 | No | CRP, leucocyte count |
| Avila-Funes, J. A., et al., 2015(Avila-Funes et al., 2015) | General medicine, Mexico | 23/141 | CAM | Yes | Cortisol |
| Brattinga B. et al., 2022 | Surgery - Oncology, Netherlands | 38/311 | DOS | No | CRP, IL-6 |
| Capri, M., et al., 2014 | Mixed Surgery, Italy | 47/74 | CAM | No | Il-6, IL-8, IL-2, IL-10, I score |
| Cerejeira, J., et al., 2013 | Elective orthopaedic, Portugal | 37/101 | CAM | Yes | Cortisol, IGF-1, leucocyte count |
| Chen, J., et al., 2022 | General Surgery – Oncology, China | 74/270 | DSM 5 | Yes | Leucocyte count, NLR |
| Chen, Y., et al., 2019 | Cardiac surgery, China | 85/266 | CAM ICU | Yes | IL-6 |
| Chu, C. S., et al., 2016 | Orthopaedic surgery, China | 23/103 | CAM | Yes | IGF-1, leucocyte count |
| Chung, K. S., et al.,2015 | Elective orthopaedic surgery, South Korea | 11/287 | DSM IV/CAM | No | CRP |
| Colkesen, Y., et al. ,2013 | Cardiac medical, Turkey | 25/52 | DSM IV | Yes | Cortisol |
| de Castro, S., et al., 2014 | Mixed surgery, Netherlands | 36/213 | DSM IV | No | CRP, leucocyte count |
| Deiner, S., et al., 2014 | Non cardiac surgery, US | 14/76 | CAM | No | Cortisol |
| Dittrich, T., et al., 2016 | Intensive Care, Switzerland | 145/240 | ICDSC | Yes | CRP, leucocyte count |
| Feng, Q., et al., 2019 | Intensive Care (Sepsis), China | 74/175 | CAM-ICU | No | Leucocyte count |
| Guenther, U., et al., 2013 | Cardiac Surgery, Germany | 69/215 | CAM-ICU | No | CRP, leucocyte count |
| Guldolf, K., et al., 2021 | Stroke, Belgium | 201/514 | DSM 5 | No | CRP, neutrophil count, lymphocyte count, NLR |
| Guo, Y., et al., 2016 | Emergency Orthopaedic Surgery, China | 120/572 | CAM | Yes | CRP, ESR, leucocyte count |
| He, R., et al., 2020 | Emergency Orthopaedic Surgery, China | 182/780 | CAM | Yes | Neutrophil count, lymphocyte count, NLR |
| Hindiskere, S., et al., 2020 | Elective Orthopaedic Surgery - Oncology, South Korea | 25/276 | DSM IV | Yes | CRP, leucocyte count |
| Jiang, X., et al., 2020 | Intensive Care, China | 29/319 | CAM-ICU | No | CRP, leucocyte count, neutrophil count, lymphocyte count, NLR, PLR |
| Katsumi, Y., et al., 2020 | Elective Orthopaedic Surgery, US | 6/36 | CAM | No | IL-6, CRP |
| Kazmierski, J., et al., 2013b | Elective Cardiac Surgery, Poland | 47/113 | CAM-ICU | No | Cortisol |
| Khan, S. H., et al., 2022 | General Surgery - Oncology, US | 26/71 | CAM-ICU | No | CRP, IGF-1 |
| Kinoshita, H., et al., 2021 | Elective Plastic Surgery - Oncology, Japan | 20/97 | ICDSC | No | NLR |
| Knaak, C., et al., 2019 | Elective surgery, Germany | 72/314 | DSM IV | No | CRP |
| Kotfis, K., et al., 2019b | Cardiac surgery, Poland | 129/968 | CAM-ICU | Yes | CRP, leucocyte count, neutrophil count, lymphocyte count, NLR, PLR |
| Lechowicz, K., et al., 2021 | Cardiac surgery, Poland | 164/1098 | DSM IV | No | CRP, leucocyte count, lymphocyte count, neutrophil count, NLR, PLR |
| Lee, H. J., et al., 2011 | Orthopaedic Surgery, South Korea | 18/65 | DRS | Yes | CRP |
| Lemstra, A. W., et al., 2008 | Emergency Orthopaedic Surgery, Netherlands | 18/68 | CAM | Yes | CRP, IL-6, IGF-1 |
| Li, X., et al., 2022 | Orthopaedic Surgery, China | 60/184 | CAM | Yes | Neutrophil count, NLR, PLR |
| Liu, P., et al., 2013 | Non-Cardiac Surgery, China | 50/338 | CAM-ICU | No | IL-6 |
| Lv, X. C., et al., 2021 | Cardiac surgery, China | 31/221 | CAM-ICU | No | Leucocyte count, IL-6 |
| Macdonald, A., et al., 2007 | Geriatric Medicine, UK | 26/84 | CAM | No | CRP |
| Miao, S., et al., 2018 | Elective General Surgery - Oncology, China | 49/112 | DSM IV | No | Neopterin, CRP, IL-6, IGF-1 |
| Mietani, K., et al., 2022a | Elective General Surgery, Oncology Japan | 15/96 | CAM-ICU | No | IL-6, CRP |
| Neerland, B. E., et al., 2016 | Emergency Orthopaedic Surgery, UK/Norway | 37/149 | CAM | Yes | CRP, IL-6 |
| Osse, R. J., et al., 2012 | Elective Cardiac Surgery, Netherlands | 63/125 | CAM-ICU | Yes | CRP |
| Oyama, T., et al, 2022 | Elective General Surgery, Oncology Japan | 20/110 | ICDSC | No | NLR, PLR |
| Pasqui, E., et al., 2022 | Vascular surgery, Italy | 73/646 | CAM | No | CRP, leucocyte count, neutrophil count, lymphocyte count, NLR, PLR |
| Peng, J., et al. 2019 | Elective Orthopaedic Surgery, China | 55/272 | DSM 5 | No | Leucocyte count, IL-6 |
| Ren, Q., et al., 2020 | Spinal surgery, China | 12/206 | CAM | No | CRP |
| Ritter, C., et al., 2014 | Intensive care, Brazil | 31/78 | CAM-ICU | Yes | IL-6 |
| Rudolph, J. L., et al., 2008 | Cardiac surgery, US | 12/24 | CAM | Yes | IL-6 |
| Sakaguchi, T., et al., 2018 | Decompensated heart failure, Japan | 38/120 | ICDSC | No | CRP, leucocyte count |
| Seo, C. L., et al., 2021 | Intensive care, South Korea | 1527/2384 | CAM-ICU | Yes | NLR, leucocyte count, neutrophil count, lymphocyte count, CRP |
| Shen, H., et al., 2016 | General Surgery - Oncology, China | 36/140 | DSM IV | Yes | IGF-1, IL-6, CRP |
| Shi, Q., et al., 2019 | Emergency cardiac surgery, China | 68/148 | CAM-ICU | No | Leucocyte count |
| Simons, K. S., et al., 2018 | Intensive care, Netherlands | 35/50 | CAM-ICU | Yes | IL-6 |
| Slor, C. J., et al., 2019 | Emergency Orthopaedic Surgery, Netherlands | 41/121 | CAM | No | CRP |
| Theologou, S., et al., 2018 | Cardiac surgery, Greece | 20/179 | CAM-ICU | Yes | CRP, NLR |
| Vasunilashorn, S. M., et al., 2015 | Elective non-cardiac surgery, US | Discovery: 39/78/Replication: 36/72, Pooled 75/150 | CAM | Yes | IL-6 |
| Vasunilashorn, S. M., et al., 2017 | Elective non-cardiac surgery, US | 134/560 | CAM | Yes | CRP |
| Visser, L., et al., 2015 | Elective vascular surgery, Netherlands | 22/463 | DOS | No | CRP |
| Xiang, D., et al., 2017 | Elective general surgery - oncology, China | 39/160 | CAM-ICU | Yes | CRP |
| Xu, W-B., et al., 2019 | Emergency general surgery, China | 49/184 | CAM | No | CRP, leucocyte count |
| Yang, J. S., et al. 2022 | Orthopaedic surgery (Spinal), South Korea | 187/2518 | NuDESC | Yes | NLR, PLR |
| Yen, T. E., et al. 2016 | Elective orthopaedic surgery, US | 22/106 | CAM | Yes | IGF-1 |
| Zhang, L., et al., 2022a | Elective orthopaedic, China | 42/268 | CAM | No | CRP, leucocyte count, lymphocyte count, NLR |
| Zhang, Z. Y., et al., 2016b | Emergency orthopaedic surgery, China | 56/179 | CAM-ICU | Yes | IL-6 |
| Zhang, Z., et al., 2014 | Intensive care, China | 54/223 | CAM-ICU | Yes | CRP |

*CRP- C-reactive protein, IL-6 – Interleukin-6 , IGF-1 – insulin-like growth factor-1, NLR – neutrophil to lymphocyte ratio, -1, PLR – platelet to lymphocyte ratio, NLR neutrophil to lymphocyte ratio*

Table 4 Records included in the quantitative synthesis that measured peripheral immune response during delirium

| **Author and Date** | **Setting** | **Number with Delirium/**  **Sample Size** | **Diagnostic Criteria** | **Definite delirium at time of test** | **Measure of Peripheral Inflammatory Response included in Meta-Analysis** |
| --- | --- | --- | --- | --- | --- |
| Adamis, D., et al., 2009 | Geriatric Medicine, UK | 28/67 | CAM | Yes | IL-6 |
| Al Tmimi, L., et al., 2015 | Cardiac surgery, Belgium | 8/42.0 | CAM-ICU | No | IL-6 |
| Alexander, S. A., et al., 2014 | Intensive care, US | 35/77 | CAM-ICU | Yes | Il-6 |
| Bisschop, P. H., et al., 2011 | Emergency orthopaedic, Netherlands | 70/112 | CAM | Yes | Cortisol |
| Cereghetti, C., et al., 2017 | Cardiac surgery, Switzerland | 244/618 | ICDS | No | CRP |
| Cerejeira, J., et al., 2013 | Elective orthopaedic, Portugal | 37/101 | CAM | No | Cortisol, IGF-1 |
| Chen, Y., et al., 2019 | Cardiac surgery, China | 85/266 | CAM ICU | No | IL-6 |
| Cizginer, S., et al., 2017 | Mixed Elective surgery, US | 134/556 | CAM | No | CRP |
| de Rooij, S. E., et al., 2007 | General Medicine, Netherlands | 64/185 | CAM | Yes | IL-6, CRP |
| Dittrich, T., et al., 2016 | Intensive Care, Switzerland | 145/240 | ICDSC | Yes | CRP, leucocyte count |
| Erikson, K., et al., 2019 | Intensive Care (Sepsis), Finland | 10/22 | CAM-ICU | Yes | CRP, IL-6 |
| Hasegawa, T., et al., 2015 | Elective Maxillofacial Surgery - Oncology, Japan | 29/188 | DSM IV | No | CRP |
| Jorge-Ripper, C., et al., 2017 | General Medicine (Sepsis), Spain | 47/82 | CAM | No | IL-6 |
| Katsumi, Y., et al., 2020 | Elective Orthopaedic Surgery, US | 6/36 | CAM | No | IL-6, CRP |
| Kazmierski, J., et al., 2013a | Elective Cardiac Surgery, Poland | 47/111 | CAM-ICU | No | IL-2, Cortisol |
| Khan, S. H., et al., 2022 | General Surgery - Oncology, US | 26/71 | CAM-ICU | No | CRP, IL-8, IL-10, TNF-α, IGF-1 |
| Knaak, C., et al., 2019 | Elective surgery, Germany | 72/314 | DSM IV | No | CRP |
| Kotfis, K., et al., 2019a | Stroke, Poland | 121/760 | CAM-ICU | Yes | NLR, CRP, neutrophil count, lymphocyte count, leucocyte count |
| Kotfis, K., et al., 2019b | Cardiac surgery, Poland | 129/968 | CAM-ICU | No | CRP, leucocyte count, neutrophil count, lymphocyte count, PLR, NLR |
| Kotfis, K., et al., 2019c | Stroke, Poland | 172/1001 | CAM-ICU | Yes | CRP, leucocyte count, neutrophil count, lymphocyte count, NLR |
| Kowalska, K., et al., 2018 | Stroke, Poland | 21/144 | DSM 5 | Yes | IL-6, leucocyte count |
| Lee, H. J., et al., 2011 | Orthopaedic Surgery, South Korea | 18/65 | DRS | No | CRP |
| Li, X., et al., 2022 | Orthopaedic Surgery, China | 60/184 | CAM | No | NLR, PLR, CRP |
| Li, Q-H., et al., 2019 | Emergency Orthopaedic Surgery, China | 67/186 | CAM | No | CRP |
| Liu, P., et al., 2013 | Non-Cardiac Surgery, China | 50/338 | CAM-ICU | No | IL-6 |
| Ma, X., et al., 2022 | Elective Orthopaedic Surgery, China | 44/143 | CAM | No | CRP |
| Macdonald, A., et al., 2007 | Geriatric Medicine, UK | 26/84 | CAM | Yes | CRP |
| Mietani, K., et al., 2022b | Elective General Surgery, Oncology Japan | 15/96 | CAM-ICU | No | IL-6, leucocyte count |
| Mu, D. L., et al., 2010 | Cardiac surgery, China | 123/243 | CAM-ICU | No | Cortisol |
| Nagase, M., et al., 2012 | Palliative care - oncology, Japan | 49/115 | MDAS | Yes | CRP, leucocyte count |
| Page, V. J., et al., 2014 | Intensive care, UK | 167/470 | CAM-ICU | Yes | CRP |
| Pfister, D., et al., 2008 | Intensive care/sepsis, Switzerland | 12/16 | CAM-ICU | No | CRP, IL-6, Cortisol |
| Plaschke, K., et al., 2013 | Non-cardiac surgery, Germany | 20/37 | CAM-ICU | Yes | IL-6 |
| Plaschke, K., et al., 2010 | Cardiac surgery, Germany | 32/114 | CAM-ICU | Yes | CRP, IL-6, leucocyte count, Cortisol |
| Pol, R. A., et al., 2014 | Vascular surgery, Netherlands | 16/277 | DSM IV | No | CRP |
| Ren, Q., et al., 2020 | Spinal surgery, China | 12/206 | CAM | No | CRP |
| Seo, C. L., et al., 2021 | Intensive care, South Korea | 1527/2384 | CAM-ICU | Yes | NLR, leucocyte count, neutrophil count, lymphocyte count, CRP |
| Simons, K. S., et al., 2018 | Intensive care, Netherlands | 35/50 | CAM-ICU | No | IL-6, leucocyte count |
| Shi, C., et al., 2010 | Non cardiac surgery, China | 73/192 | Nu-DESC | No | Cortisol |
| Skrobik, Y., et al., 2013 | Intensive Care, Canada | 7/21 (only delirium vs no delirium or coma) | ICDSC | Yes | IL-6 |
| Soler-Sanchis, A., et al, 2022 | Emergency department - Geriatric, Spain | 128/256 | DSM 5 | Yes | CRP, leucocyte count, lymphocyte count, neutrophil count, NLR, PLR |
| Theologou, S., et al., 2018 | Cardiac surgery, Greece | 20/179 | CAM-ICU | No | CRP, NLR |
| Tsuruta, R., et al., 2010 | Intensive Care, Japan | 21/103 | CAM-ICU | No | CRP |
| van den Boogaard, M., et al., 2011a | Intensive Care, Netherlands | 50/100 | CAM-ICU | Yes | CRP, Cortisol |
| Van Munster, B. C., et al., 2008 | Emergency orthopaedic, Netherlands | 50/98 | CAM | Yes | Il-6 |
| van Munster, B. C., et al., 2010 | Emergency orthopaedic, Holland | 62/120 | CAM | Yes | IL-6, Cortisol |
| Vasunilashorn, S. M., et al., 2015 | Elective non-cardiac surgery, US | Discovery: 39/78/Replication: 36/72, Pooled 75/150 | CAM | No | IL-6 |
| Vasunilashorn, S. M., et al., 2017 | Elective non-cardiac surgery, US | 134/560 | CAM | No | CRP |
| Winkelman, C., et al., 2018 | Intensive care, US | 27/44 | CAM-ICU | Yes | IL-6, CRP |
| Xiang, D., et al., 2017 | Elective general surgery - oncology, China | 39/160 | CAM-ICU | Yes | CRP |
| Zhang, L. N., et al., 2016a | Intensive care (Sepsis), China | 29/59 | CAM-ICU | Yes | CRP, IL-6 |

*CRP- C-reactive protein, IL-6 – Interleukin-6 , IGF-1 – insulin-like growth factor-1, NLR – neutrophil to lymphocyte ratio, -1, PLR – platelet to lymphocyte ratio, NLR neutrophil to lymphocyte ratio*

## Summary Tables of Studies Included in Qualitative Analysis

Table 5 Records included in the Qualitative Synthesis that measured peripheral immune response preceding delirium

| **Author and Date** | **Setting** | **Number with Delirium/Sample Size** | **Diagnostic Criteria** | **Confirmed delirium free at time of test** | **Measure of Peripheral Inflammatory Response** | **Key Findings** |
| --- | --- | --- | --- | --- | --- | --- |
| Alvarez-Perez, F. J. and F. Paiva, 2017 | Stroke, Portugal | 118/1072 | DSM 5 | No | CRP, ESR, leucocyte count | No significant difference in peripheral markers measured in those that developed delirium and those that did not. |
| Avila-Funes, J. A., et al., 2015 | General medicine, Mexico | 23/141 | CAM | Yes | Cortisol | Cortisol levels were higher in the delirium group compared to the no delirium group, but results did not reach significance. |
| Ballweg T. et al., 2021 | Elective surgery, US | 38/110 | DRS/CAM ICU | No | IL-1β, IL-1ra, IL-2, IL-4, IL-6, IL-8, IL-10, IL-12p70, MCP-1, TNF-α | IL-1β, IL-1RA and IL-2 significantly higher in those that developed delirium compared to those that did not. No significant difference in other peripheral markers of inflammation measured. |
| Baranyi, A., and Rothenhausler, H. B., 2014 | Cardiac Surgery, Germany | 11/34 | DRS | No | sIL-2R, CRP | No difference in pre-operative mean CRP in those that developed delirium compared to those that did not. |
| Brattinga B. et al., 2022 | Surgery - Oncology, Netherlands | 38/311 | DOS | No | CRP, IL-1β, IL-6, IL-10, NGAL | No difference in pre-operative inflammatory markers between those that developed delirium and those that did not. |
| Capri, M., et al., 2014 | Mixed Surgery, Italy | 47/74 | CAM | No | Il-6, IL-8, IL-2, IL-10, I score | High Il-6 and low IL-2 pre-operatively were associated with postoperative delirium. Il-6 association was independent of confounders. No association between other inflammatory cytokines and delirium. |
| Cerejeira, J., et al., 2013 | Elective orthopaedic, Portugal | 37/101 | CAM | Yes | Cortisol, IGF-1, CRP, IL-6, IL-8, IL-10, I score, leucocyte count | No significance difference in pre-operative cortisol, IGF-1, CRP or inflammatory cytokine levels in patients with and without post-operative delirium. |
| Cerejeira, J., et al., 2012 | Elective orthopaedic, Portugal | 37/101 | CAM | Yes | CRP, IL-6, IL-8, IL-10, IL-1β, TNF-α, I score | No difference in pre-operative inflammatory markers between those that developed post-operative delirium and those that did not. |
| CheheiliSobbi S. et al., 2021 | Cardiac surgery, Netherlands | 14/89 | CAM ICU | No | Leukocyte cytokine production (IL-6, IL-8, IL-10, TNF-α) | No significant difference in pre-operative leukocyte cytokine production between those that developed post-operative delirium and those that did not. |
| Chen, J., et al., 2022 | General Surgery – Oncology, China | 74/270 | DSM 5 | Yes | Leucocyte count, NLR | NLR an independent risk factor for postoperative delirium. |
| Chen, Y., et al., 2019 | Cardiac surgery, China | 85/266 | CAM ICU | Yes | IL-6 | Pre-operative IL-6 levels not significantly higher in patients with post-operative delirium. |
| Chen, X. W., et al., 2014 | Emergency orthopaedic, China | 70/186 | CAM | No | Leptin | Pre-operative leptin an independent predictor of post-operative delirium. |
| Chu, C. S., et al., 2016 | Orthopaedic surgery, China | 23/103 | CAM | Yes | IGF-1, leucocyte count | Pre-operative IGF-1 and leucocyte count did not differ between those that developed post-operative delirium and those that did not. |
| Chung, K. S., et al.,2015 | Elective orthopaedic surgery, South Korea | 11/287 | DSM IV/CAM | No | CRP, ESR | Pre-operative CRP and ESR did not differ between those that developed post-operative delirium and those that did not. |
| Colkesen, Y., et al. ,2013 | Cardiac medical, Turkey | 25/52 | DSM IV | Yes | Cortisol | Pre-op Cortisol significantly higher in those that developed delirium compared with those that did not. Independent when controlling for confounders. |
| de Castro, S., et al., 2014 | Mixed surgery, Netherlands | 36/213 | DSM IV | No | CRP, leucocyte count | Pre-op CRP not significantly higher in those that developed delirium compared to those that did not. |
| Deiner, S., et al., 2014 | Non cardiac surgery, US | 14/76 | CAM | No | Cortisol | No difference in pre-operative Cortisol between those that developed delirium and those that did not. |
| Dillon, S. T., et al., 2017 | Non cardiac surgery, US | 75/150 | CAM | Yes | CRP | CRP was significantly higher in the delirium group pre-operatively compared to the no delirium group. Independent after controlling for confounders. |
| Dittrich, T., et al., 2016 | Intensive Care, Switzerland | 145/240 | ICDSC | Yes | CRP, leucocyte count | No difference in CRP or leucocyte count on the day of blood stream infection diagnosis in those with delirium and those without. |
| Eshmawey, M., et al., 2019 | Elective cardiac surgery, Germany | 60/183 | CAM-ICU | No | Cortisol, CRP | Pre-operative CRP and Cortisol were not significantly higher in those with delirium compared to those without. |
| Feng, Q., et al., 2019 | Intensive Care (Sepsis), China | 74/175 | CAM-ICU | No | Leucocyte count, neutrophil fraction, lymphocyte fraction | Leucocyte count significantly lower in those with septic associated encephalopathy than those with sepsis along. No difference in other inflammatory markers. |
| Fernandez-Jimenez, E., at al., 2021 | COVID-19, Spain | 225/1785 | DSM 5 | No | NLR | NLR on admission predicted subsequent delirium development. Risk of delirium increased twofold in patients between 69 and 80 years with NLR values > 6.3. |
| Girard, T. D., et al., 2012 | Intensive Care (Medicine), Australia | 107/138 | CAM ICU | Yes | CRP, sTNFR1, MPO, NGAL | Higher CRP and sTNFR1 associated with increased probability of delirium. In multivariate analysis association remained for sTNFR1, but not CRP. |
| Guenther, U., et al., 2013 | Cardiac Surgery, Germany | 69/215 | CAM-ICU | No | CRP, leucocyte count | Pre-operative CRP significantly higher in those that developed delirium compared to those that did not. No difference in leucocyte count between groups. |
| Guldolf, K., et al., 2021 | Stroke, Belgium | 201/514 | DSM 5 | No | CRP, neutrophil count, lymphocyte count, NLR | NLR, neutrophil count higher and lymphocyte count lower on admission in those that developed delirium compared to those that did not. No difference in CRP. NLR was an independent predictor of post stroke delirium. |
| Guo, Y., et al., 2016 | Emergency Orthopaedic Surgery, China | 120/572 | CAM | Yes | CRP, ESR, leucocyte count, neutrophil fraction | Higher pre-operative CRP, leucocyte count and neutrophil percentage is significantly associated with post-operative delirium. No difference in ESR. CRP association independent when controlling for confounders. |
| Hatta, K., et al., 2014 | Medical and surgical patients, Japan | 9/29 | DSM IV | Yes | NK cell activity, monocyte IL-Iβ production | No difference in NK cell activity or monocyte IL-1β production on the first morning in patients developing delirium compared to those without delirium |
| He, R., et al., 2020 | Emergency Orthopaedic Surgery, China | 182/780 | CAM | Yes | Neutrophil count, lymphocyte count, NLR | Patients that developed POD had a significantly higher NLR and neutrophil count at baseline compared to those that did not. No difference in Lymphocyte count. NLR predictive of POD with AUC of 0.83. |
| Hindiskere, S., et al., 2020 | Elective Orthopaedic Surgery - Oncology, South Korea | 25/276 | DSM IV | Yes | CRP, leucocyte count, ESR | CRP significantly higher pre-operatively in patients who developed delirium compared to those who did not. Independently associated after controlling for confounders. |
| Jiang, X., et al., 2020 | Intensive Care, China | 29/319 | CAM-ICU | No | CRP, leucocyte count, neutrophil count, lymphocyte count, NLR, PLR | PLR on admission significantly higher in patients the developed delirium compared to those that did not. A PLR >100 independently predicted delirium. |
| Kazmierski, J., et al., 2022 | Elective Cardiac Surgery, Poland | 61/177 | CAM-ICU | Yes | MPO | Pre-operative MPO concentration significantly higher in patients that went on to develop delirium compared to those that did not. Not independent. |
| Kazmierski, J., et al., 2013b | Elective Cardiac Surgery, Poland | 47/113 | CAM-ICU | No | IL-2, cortisol, TNF-α | Preoperative cortisol significantly higher in those that developed delirium compared to those that did not. |
| Kazmierski, J., et al., 2014 | Elective Cardiac Surgery, Poland | 47/113 | CAM-ICU | No | IL-2, Cortisol | Preoperative cortisol levels independently associated with delirium. |
| Khan, S. H., et al., 2022 | General Surgery - Oncology, US | 26/71 | CAM-ICU | No | CRP, IL-8, IL-10, TNF-α, IGF-1 | Pre-operative inflammatory marker concentrations were not associated with post-operative delirium. |
| Kim, M. Y., et al., 2016 | General Surgery, South Korea | 112/561 | CAM | Yes | CRP, leucocyte count | Pre-operative CRP of >10mg/dL independently associated with delirium. |
| Kinoshita, H., et al., 2021 | Elective Plastic Surgery - Oncology, Japan | 20/97 | ICDSC | No | NLR | Pre-operative NLR significantly higher in those that developed delirium compared to those that did not. |
| Knaak, C., et al., 2019 | Elective surgery, Germany | 72/314 | DSM IV | No | CRP | Pre-operative CRP significantly higher in those that developed delirium to compared to those that did not. Independent predictor in multivariate analysis. |
| Kotfis, K., et al., 2019b | Cardiac surgery, Poland | 129/968 | CAM-ICU | Yes | CRP, leucocyte count, neutrophil count, lymphocyte count, PLR, PWR, NLR | Pre-operative leucocyte count and CRP were higher, and PWR and PLR were lower, in those that developed delirium compared to those that did not. |
| Kozak, H. H., et al., 2017 | Stroke, Turkey | 11/60 | DSM 5 | No | TNF-α, IL-18, IL-1β, CRP, leucocyte count | No significant difference in inflammatory markers between those that developed delirium and those that did not. |
| Kupiec, A., et al., 2020 | Cardiac Surgery, Poland | 30/149 | CAM-ICU | No | PCT | Pre-operative PCT above the reference range was recorded more commonly in those that developed delirium than those that did not. |
| Lammers-Lietz, F., et al., 2022 | Mixed Surgery, Germany | BIO-COG: 125/504 PHYDELIO:6/32 | DSM-IV-TR/ DSM5 | No | IL-8 | Pre-operative IL-8 levels higher in those who went on to develop delirium compared to those that did not in the PHYDELIO group. No difference in the BIO-COG group. |
| Lechowicz, K., et al., 2021 | Cardiac surgery, Poland | 164/1098 | DSM IV | No | CRP, leucocyte count, lymphocyte count, neutrophil count, NLR, PLR, PWR | Pre-operative CRP, leucocyte count and Neutrophils were significantly higher, and PWR significantly lower, in those that went on to develop delirium compared to those that did not. |
| Lee, H. J., et al., 2011 | Orthopaedic Surgery, South Korea | 18/65 | DRS | Yes | CRP | Pre-operative CRP not significantly higher in those that developed delirium compared to those that did not. |
| Lemstra, A. W., et al., 2008 | Emergency Orthopaedic Surgery, Netherlands | 18/68 | CAM | Yes | CRP, IL-6, IGF-1 | Pre-operative CRP, IL-6 or IGF-1 did not differ between the two groups. |
| Li, X., et al., 2022 | Orthopaedic Surgery, China | 60/184 | CAM | Yes | Neutrophil count, NLR, PLR | Pre-operative Neutrophils and NLR were significantly higher, and lymphocytes and PLR lower in those that developed delirium compared to those that did not. |
| Li, G., et al., 2017 | Intensive Care, China | 11/336 | CAM-ICU | Yes | Leptin | Lower Leptin at ICU entry was independently associated with subsequent occurrence of delirium. |
| Liu, P., et al., 2013 | Non-Cardiac Surgery, China | 50/338 | CAM-ICU | No | IL-6 | IL-6 not significantly higher in those that developed delirium compared to those that did not but pre-operative IL-6 level ≥7.5pg/ml independently associated with increased risk of delirium. |
| Lv, X. C., et al., 2021 | Cardiac surgery, China | 31/221 | CAM-ICU | No | Leucocyte count, IL-6, PCT | Pre-operative IL-6 and leucocyte count significantly higher in those that developed postoperative delirium compared to those that did not. |
| Macdonald, A., et al., 2007 | Geriatric Medicine, UK | 26/84 | CAM | No | CRP | High CRP independent predictor of incident delirium. |
| Mao, M., et al., 2022 | Elective orthopaedic surgery, China | 35/131 | CAM | Yes | IL-17, IL-33, C3a, C3, C5a, VCAM-1, e-selectin | No pre-operative inflammatory markers measured were significantly higher in those that developed delirium compared to those that did not. |
| McManus, J., et al., 2009 | Stroke, UK | 23/82 | CAM | No | CRP | Development of delirium associated with raised CRP on admission (>3mg/L). Remained significant after controlling for confounders. |
| Miao, S., et al., 2018 | Elective General Surgery - Oncology, China | 49/112 | DSM IV | No | Neopterin, CRP, IL-6, IGF-1 | Pre-operative levels of neopterin, CRP, IL‐6, were significantly higher, levels of IGF-1 significantly lower in patients with a delirium than those without a delirium. Neopterin was an independent predictor. |
| Mietani, K., et al., 2022a | Elective General Surgery, Oncology Japan | 15/96 | CAM-ICU | No | IL-6, PAI-1, PECAM-1, p-selectin, CRP | Pre-operative levels of PAI-1 were significantly lower in those that developed delirium compared to those that did not. |
| Mietani, K., et al., 2022b | Elective General Surgery, Oncology Japan | 15/96 | CAM-ICU | No | IL-6, PAI-1, PECAM-1, p-selectin, leucocyte count | IL-6 significantly higher in those that developed delirium compared to those that did not. |
| Morandi, A., et al., 2011 | Intensive care, US | 40/62 | CAM-ICU | No | IGF-1 | IGF-1 not a risk factor for delirium development the following day. |
| Nakamura, J., et al., 2001 | Cardiac surgery, Japan | 11/26 | ICD-10 | No | NK cell activity | No difference in NK cell activity showed between those with delirium and those without. |
| Nydahl, P., et al., 2017 | Stroke, Germany | 33/309 | CAM | No | CRP | Raised CRP (>8mmol/L) on admission associated with delirium. |
| Osse, R. J., et al., 2012 | Elective Cardiac Surgery, Netherlands | 63/125 | CAM-ICU | Yes | Neopterin, CRP | Pre-operative CRP and Neopterin significantly higher in those that developed POD compared to those that did not. Association for Neopterin only remained in multivariate analysis. |
| Oyama, T., et al, 2022 | Elective General Surgery, Oncology Japan | 20/110 | ICDSC | No | NLR, PLR | Pre-operative NLR, but not PLR, associated with post-operative delirium. NLR ≥ 2.45 independently predictive of delirium. |
| Pasqui, E., et al., 2022 | Vascular surgery, Italy | 73/646 | CAM | No | CRP, leucocyte count, neutrophil count, lymphocyte count, NLR, PLR, SII | Pre-operative CRP, leucocyte count, neutrophil count, NLR, PLR and SII significantly higher in those that developed delirium. Lymph significantly lower. NLR >3.57 and SII >676.4 independent risk factors for POD. |
| Peng, J., et al. 2019 | Elective Orthopaedic Surgery, China | 55/272 | DSM 5 | No | Leucocyte count, IL-6, TNF-α, CAR | IL-6, TNF-α and CAR predictive of delirium. CAR independently predictive of delirium. |
| Pol, R. A., et al., 2011 | Vascular surgery, Netherlands | 10/142 | DSM-IV | No | CRP, leucocyte count | Raised pre-operative CRP is associated with post-op delirium. |
| Ren, Q., et al., 2020 | Spinal surgery, China | 12/206 | CAM | No | CRP | No association between pre-operative CRP and delirium |
| Ritter, C., et al., 2014 | Intensive care, Brazil | 31/78 | CAM-ICU | Yes | IL-1β, IL-6, TNF-α, STNFR1, STNFR2, IL-10 | Il-1β, TNF-α, STNFR1, STNFR2 were significantly higher in patients who developed delirium compared to those that did not. IL-1β, log STNFR1 and log STNFR2 independent association with delirium in multivariate regression. |
| Rudolph, J. L., et al., 2008 | Cardiac surgery, US | 12/24 | CAM | Yes | IL-1β, IL-1RA, IL-2, IL-2R, IL-4, IL-5, IL-6, IL-7, IL-8, IL-10, IL-13, IL-12p40_p70, IL-15, IL-17, IFN-γ, TNF-α, sTNFR1, sTNFR2, IP-10, MIP-1α, MIP-1β, Eotaxin, RANTES, CCL-2, GM-CSF, DR5, MIG | Pre-operative CCL2 levels significantly high in those that developed delirium compared to those that did not. No difference in inflammatory cytokine levels. |
| Sakaguchi, T., et al., 2018 | Decompensated heart failure, Japan | 38/120 | ICDSC | No | CRP, leucocyte count | Admission CRP significantly higher in those that developed delirium compared to those that did not. |
| Shen, H., et al., 2016 | General Surgery - Oncology, China | 36/140 | DSM IV | Yes | IGF-1, IL-6, CRP | Pre-operative IGF-1 was significantly lower, and CRP and IL-6 significantly higher, in those that developed delirium compared to those that did not. |
| Shi, Q., et al., 2019 | Emergency cardiac surgery, China | 68/148 | CAM-ICU | No | Leucocyte count | Leucocyte count is not predictive of post-operative delirium. |
| Simons, K. S., et al., 2018 | Intensive care, Netherlands | 35/50 | CAM-ICU | Yes | IL-1β, IL-6, IL-10, TNF-α, MCP-1, neopterin | Immediately prior to delirium onset there were no differences in the levels of any of the markers in those who developed delirium compared to those that did not. |
| Slor, C. J., et al., 2019 | Emergency Orthopaedic Surgery, Netherlands | 41/121 | CAM | No | CRP | Higher CRP levels associated with delirium using GEE method, after controlling for confounders. |
| Song, Y., et al., 2022 | Non-cardiac and non-neurosurg Surgery, China | 927/29608 | DSM-IV | Yes | SII (NLR x platelets) | SII (NLR x platelets) an independent risk factor for POD. |
| Sun, L., et al., 2016 | Elective Maxillofacial Surgery - Oncology, China | 56/257 | CAM | No | PCT, Cortisol, IL-6, CRP | There were no differences in pre-operative inflammatory markers between those that developed delirium and those that did not. |
| Theologou, S., et al., 2018 | Cardiac surgery, Greece | 20/179 | CAM-ICU | Yes | CRP, NLR | No significant difference in NLR or CRP in those that develop delirium compared to those that did not. |
| Vasunilashorn, S. M., et al, 2019 | Elective non- cardiac surgery, US | 10/20 | CAM | Yes | Histidine-rich glycoprotein (HRG), Complement C2, Complement component C9 (CO9), Complement C4-B (CO4B), Complement factor I (CFAI) 67 75 3 6, CRP, Complement factor D, Complement C4-A, Alpha-1-antichymotrypsin, alpha-1-acid glycoprotein | CRP and AZGP1 was the “best combined model” for delirium preoperatively. |
| Vasunilashorn, S. M., et al., 2022 | Elective non- cardiac surgery, US | 18/36 | CAM | Yes | Chitinase-3-like protien 1, platelet factor 4, MHC class I polypeptide-related sequence A, resistin, CMRF35-like molecule 6 (CD300C), Programmed cell death 1 ligand 1, Low affinity immunoglobulin gamma Fc region receptor III-B, Tumor necrosis factor receptor superfamily member 1A, Complement C4b, CCL27, Tumor necrosis factor ligand superfamily member 9, Eotaxin. IL-25, IL-6, MHC class I polypeptide-related sequence A, CCL16, Plasma protease C1 inhibitor | Chitinase-3-like-protein-1 (CHI3L1/YKL-40) was identified as the sole delirium-associated protein in a preoperative predictor model. Multi-protein modelling found high preoperative CHI3L1/YKL-40 increased the risk of delirium. |
| Vasunilashorn, S. M., et al., 2015 | Elective non-cardiac surgery, US | Discovery: 39/78/Replication: 36/72, Pooled 75/150 | CAM | Yes | IL-1β, IL-2, IL-4, IL-5, IL-6, IL-8, IL-10, IL-12, IFN-ɣ, GM-CSF, TNF-α | In the discovery cohort IL-1β and IL-2 was significantly higher pre-operatively and in recovery in those that went on to develop delirium compared to those that did not. In the pooled cohort only IL-2 was significantly higher. |
| Vasunilashorn, S. M., et al., 2017 | Elective non-cardiac surgery, US | 134/560 | CAM | Yes | CRP | After adjusting for confounders patients with preop CRP≥3 had a greater risk of delirium. |
| Visser, L., et al., 2015 | Elective vascular surgery, Netherlands | 22/463 | DOS | No | CRP | Perioperative CRP significant in univariate analysis but not when controlling for confounders. |
| Wanderlind, M. L. Z., et al., 2020 | Intensive care, Brazil | 47/94 | CAM-ICU | Yes | IL-1β | IL-1β significantly higher on day 1 of ITU admission in those that went on to develop delirium compared to those that did not. |
| Wang, B., et al., 2022 | Orthopaedic surgery, China | 22/200 | CAM | No | microRNA-320, IGF-1 | No difference in plasma IGF-1 in POD and matched non-POD group. microRNA320 upregulated in POD group compared to non-POD group and IGF-1 mRNA downregulated suggesting micro-RNA 320 important in delirium pathophysiology. |
| Watts, G., et al., 2007 | Intensive care, Australia | 21/56 | ICDSC | Yes | CRP, leucocyte count, neutrophil count | Percentage of patients with a high CRP, leucocyte count, neutrophil count not higher in those with delirium compared to those without. |
| Wilson, K., et al., 2005 | General medicine, UK | 12/100 | CAM | Yes | IGF-1 | IGF-1 independently associated with the development of delirium. |
| Xiang, D., et al., 2017 | Elective general surgery - oncology, China | 39/160 | CAM-ICU | Yes | CRP | Preoperative CRP concentrations were the only independent predicator for delirium. |
| Xu, W-B., et al., 2019 | Emergency general surgery, China | 49/184 | CAM | No | CRP, sFGL2, leucocyte count | CRP and WCC significantly higher in delirium. sFLG2 was an independent predictor for delirium. |
| Yang, J. S., et al. 2022 | Orthopaedic surgery (Spinal), South Korea | 187/2518 | NuDESC | Yes | NLR, PLR, MLR, CAR | CAR, NLR and MLR significantly higher in those that went on to develop delirium compared to those that did not. None were independent predictors. ROC analysis revealed all were poor at discriminating by delirium status. |
| Yen, T. E., et al. 2016 | Elective orthopaedic surgery, US | 22/106 | CAM | Yes | IGF-1 | No difference in pre-operative IGF-1 levels in those that developed delirium compared to those that did. |
| Zhang, L., et al., 2022a | Elective orthopaedic, China | 42/268 | CAM | No | CRP, CAR, leucocyte count, lymphocyte count, NLR | CRP and CAR significantly higher in group that developed POD. Association did not remain in multivariate analysis. No difference in leucocyte count, lymph or NLR. |
| Zhang, S., et al., 2022b | Cardiac surgery, China | 72/242 | CAM-ICU | No | IL-6 | IL-6 pre-operatively was not predictive. |
| Zhang, W., et al., 2022c | Orthopaedic surgery, China | 13/53 | CAM | Yes | IL-1β, IL-6, TNF-α | No difference in IL-6, IL-1B, and TNF-α preoperatively in those that developed delirium and those that did not. |
| Zhang, Z. Y., et al., 2016b | Emergency orthopaedic surgery, China | 56/179 | CAM-ICU | Yes | IL-6, IL-8, IL-1β, TNF-α | No difference in baseline cytokines in the delirium group when compared with the no delirium group. |
| Zhang, Z., et al., 2014 | Intensive care, China | 54/223 | CAM-ICU | Yes | CRP | CRP independently associated with risk of delirium. Change in CRP greater than 8.1 mg/L showed a significantly increased risk of the development of delirium as compared with those with change in CRP less than −9.8 mg/L. |
| Zhao, Y., et al., 2021 | General medicine, China | 101/740 | CAM | Yes | NLR | Patients with delirium had a significantly higher median NLR than those without delirium. |

*CRP- C-reactive protein, ESR- erythrocyte sedimentation rate, IL-1β- interleukin-1β, IL-1ra – Interleukin-1 receptor agonist, IL-2- Interleukin-2, IL-4 – Interleukin-4, IL-6 – Interleukin-6, IL-8 – Interleukin-8, IL-10 – Interleukin-10, IL-12 – Interleukin-12, MCP-1 – Monocyte chemoattractant protein-1, TNF-α – Tumour necrosis factor-α, sIL-2R – soluble interleukin-2 receptor, NGAL – neutrophil gelatinase-associated lipocalin, I score – inflammatory score, IGF-1 – insulin-like growth factor-1, NLR – neutrophil to lymphocyte ratio, sTNFR1, soluble tumour necrosis factor receptor-1, MPO – myeloperoxidase, PLR – platelet to lymphocyte ratio, PWR – platelet to white blood cell ratio, NLR neutrophil to lymphocyte ratio, IL-18 – interleukin-18, PCT – procalcitonin, IL-17 – Interleukin-17, IL-33, Interleukin-33, C3a – Complement component 3a, C3 – Complement component 3, C5a - – Complement component 5a, PAI-1 – Plasminogen activator inhibitor-1, SII - Systemic Immune-Inflammation Index , CAR – CRP to albumin ratio, sTNFR2 – soluble tumour necrosis factor receptor 2, IL-2R – Interleukin-2 receptor, IL-5 – Interleukin-5, IL-7 – interleukin-7, IL-13 – Interleukin-13, IL-15 – interleukin-15, IFN-γ – Interferon gamma, IP-10 - Interferon gamma-induced protein 10, MIP-1α - Macrophage inflammatory protein-1α, GM-CSF – Granulocyte macrophage colony stimulating factor, DR5 – death receptor 5, MIG – monokine induced interferon-gamma, IL-25 – Interleukin-25, sFGL2 – soluble fibrinogen like protein 2.*

Table 6 Records included in the qualitative synthesis that measured peripheral immune response during delirium

| **Author and Date** | **Setting** | **Number with Delirium/Sample Size** | **Diagnostic Criteria** | **Definite delirium at time of test** | **Measure of Peripheral Inflammatory Response** | **Key Findings** |
| --- | --- | --- | --- | --- | --- | --- |
| Adamis, D., et al., 2007 | Geriatric Medicine, UK | 42/164 | CAM | Yes | IL-1β, IL-1ra, IL-6, TNF-α, IGF-I, IFN-γ, LIF | Low levels of IGF-1 associated with prevalent delirium. |
| Ballweg T. et al., 2021 | Elective surgery, US | 38/110 | DRS/CAM ICU | No | IL-1β, IL-1ra, IL-2, IL-4, IL-6, IL-8, IL-10, IL-12p70, MCP-1, TNF-α | Significant difference in the change in IL-1β, IL-1ra, IL-2, IL-8, IL-10 and MCP-1 in those with delirium compared to those without. |
| Baranyi, A. and Rothenhausler, H. B., 2014 | Cardiac Surgery, Germany | 11/34.0 | DRS | No | sIL-2R, CRP | No difference in mean CRP at any point postoperatively in those that developed delirium and those that did not. sIL-2R levels significantly higher on post-operative day 1 in those with delirium (lasting >1 day) compared to those without. |
| Beloosesky, Y., et al., 2007 | Emergency orthopaedic surgery, Israel | 11/42.0 | CAM | No | CRP, IL-8, IL-6, IL-1β, TNF- α, IL-10, IL-1RA | Significant difference in CRP and IL-8 kinetic curve in those with delirium compared to those without delirium or other post-op complications. No difference in other cytokine kinetic curves. |
| Beloosesky, Y., et al., 2004 | Emergency orthopaedic surgery, Israel | 10/32.0 | CAM | No | CRP, ESR | Significant difference in CRP kinetic curve in those with delirium compared to those without delirium or other post-op complications. No difference in ESR kinetic curve. |
| Bisschop, P. H., et al., 2011 | Emergency orthopaedic, Netherlands | 70/112 | CAM | Yes | Cortisol | Cortisol significantly higher in patients with delirium compared to those without but not significant after controlling for confounders. |
| Brum, C., et al., 2015 | Oncology, Brazil | 17/45 | CAM | Yes | TNF- α | No difference in TNF-α levels between oncology patients with and without delirium. |
| Burkhart, C. S., et al., 2010 | Cardiac surgery, Switzerland | 45/113 | CAM | No | CRP | Maximum value of CRP post-operatively was independently associated with delirium. |
| Casey CP. Et al., 2020 | Elective surgery, US | 39/108 | DRS/CAM ICU | No | IL-8 | Significantly greater change in IL-8 between pre-op and postoperative day 1 in those with delirium compared to those without. Higher IL-8 also associated with delirium severity. |
| Cereghetti, C., et al., 2017 | Cardiac surgery, Switzerland | 244/618 | ICDS | No | CRP | Maximum postoperative CPR independently associated with postoperative delirium. |
| Cerejeira, J., et al., 2013 | Elective orthopaedic, Portugal | 37/101 | CAM | No | Cortisol, IGF-1, CRP, IL-6, IL-8, IL-10, I score | Post-operative cortisol significantly higher in patients that developed post-operative delirium. No association between postoperative CRP, IGF-1 or inflammatory cytokines and postoperative delirium found. |
| Cerejeira, J., et al., 2012 | Elective orthopaedic, Portugal | 37/101 | CAM | No | CRP, IL-6, IL-8, IL-10, IL-1β, TNF- α, I score | No significant difference in inflammatory markers post-operatively in those with delirium and those without. |
| CheheiliSobbi, S., et al., 2021 | Cardiac surgery, Netherlands | 14/89 | CAM ICU | No | Leukocyte cytokine production (IL-6, IL-8, IL-10, TNF- α) | No significant difference in post-operative leukocyte cytokine production between those that developed post-operative delirium and those that did not. |
| Chen, Y., et al., 2019 | Cardiac surgery, China | 85/266 | CAM ICU | No | IL-6 | Post-operative IL-6 levels and change in IL-6 level significantly higher in patients with post-operative delirium. |
| Cizginer, S., et al., 2017 | Mixed Elective surgery, US | 134/556 | CAM | No | CRP | Participants with delirium had significantly higher CRP on POD2 compared to those without delirium. |
| de Rooij, S. E., et al., 2007 | General Medicine, Netherlands | 64/185 | CAM | Yes | TNF- α, IL-1β, IL-10, IL-8, IL-6, CRP | Inflammatory markers were not significantly higher in those with delirium compared with those without. However significantly more IL-6 levels and IL-8 levels were above the detection limit, this association remained after controlling for confounders. |
| Dillon, S. T., et al., 2017 | Non cardiac surgery, US | 75/150 | CAM | No | CRP | CRP was significantly higher in the delirium group at post-operative day 2 compared to the no delirium group. Independent after controlling for confounders. |
| Dittrich, T., et al., 2016 | Intensive Care, Switzerland | 145/240 | ICDSC | Yes | CRP, leucocyte count | No difference in CRP or leucocyte count the day before blood stream infection diagnosis in those that developed delirium and those that did not. |
| Erikson, K., et al., 2019 | Intensive Care (Sepsis), Finland | 10/22 | CAM-ICU | Yes | CRP, PCT, IL-6, TNF- α, IL-17 | IL-6 was significantly higher in those with delirium compared to those without. No other difference in inflammatory markers. |
| Fassbender, K., et al., 1994 | Stroke, Germany | 9/23 | DSM III-R | No | Cortisol | No difference in cortisol at any time point or maximal cortisol between those with delirium and those without. |
| Hasegawa, T., et al., 2015 | Elective Maxillofacial Surgery - Oncology, Japan | 29/188 | DSM IV | No | CRP | Postoperative CRP significantly higher in those with delirium compared to those without. |
| Hatta, K., et al., 2014 | Medical and surgical patients, Japan | 9/29 | DSM IV | No | NK cell activity, Monocyte IL-Iβ production | Mean change in blood NK cell activity on the second morning, compared to the first morning, in patients developing delirium was significantly greater than that in patients without delirium. No difference change in monocyte production of IL-1β on the first to second morning in those with delirium compared to those without. |
| John, M., et al., 2017 | Cardiac Surgery, Germany | 60/217 | CAM-ICU | No | CRP | Patients with delirium had a significantly higher CRP than those without. |
| Kazmierski, J., et al., 2022 | Elective Cardiac Surgery, Poland | 61/177 | CAM-ICU | No | MPO | Post-operative MPO concentration independently associated with delirium. |
| Kazmierski, J., et al., 2013a | Elective Cardiac Surgery, Poland | 47/111 | CAM-ICU | No | IL-2, cortisol, TNF-α | Postoperative cortisol, TNF-α and IL-2 significantly higher in those with delirium compared to those without. |
| Kazmierski, J., et al., 2014 | Elective Cardiac Surgery, Poland | 47/111 | CAM-ICU | No | IL-2, Cortisol | Postoperative cortisol and IL-2 significantly in those with delirium as opposed to those without. Not independent |
| Kazmierski, J., et al., 2013b | Elective Cardiac Surgery, Poland | 47/111 | CAM-ICU | No | IL-2, TNF-α | Raised IL-2 and TNF-α independently associated with delirium. Optimal cut-offs for IL-2 and TNF-α concentrations in predicting the development of delirium were 907.5 U/ml and 10.95 pg/ml, respectively. |
| Khan, S. H., et al., 2022 | General Surgery - Oncology, US | 26/71 | CAM-ICU | No | CRP, IL-8, IL-10, TNF-α, IGF-1 | CRP level on postoperative day 1 was significantly higher in those with delirium than those without delirium. No difference in any other inflammatory markers. |
| Knaak, C., et al., 2019 | Elective surgery, Germany | 72/314 | DSM IV | No | CRP | Maximum post-operative CRP not significantly higher in those with delirium to compared to those without. |
| Kotfis, K., et al., 2019a | Stroke, Poland | 121/760 | CAM-ICU | Yes | NLR, CRP, PWR, neutrophil count, lymphocyte count, leucocyte count, PNR | NLR, CRP, leucocyte count and neutrophil count were significantly higher in patients with delirium compared to those without. PWR, PNR and lymphocytes was significantly lower. |
| Kotfis, K., et al., 2019b | Cardiac surgery, Poland | 129/968 | CAM-ICU | No | CRP, leucocyte count, neutrophil count, lymphocyte count, PLR, PWR, NLR | PWR significantly lower, in those with delirium compared to those without on post-operative day 1. Lymphocyte count, neutrophil count and NLR significantly higher and PWR significantly lower in delirium on post-operative day 3. |
| Kotfis, K., et al., 2019c | Stroke, Poland | 172/1001 | CAM-ICU | Yes | CRP, leucocyte count, neutrophil count, lymphocyte count, NLR | Leucocyte count, neutrophil count, NLR and CRP were significantly higher and lymphocytes lower, in those with delirium compared to those without. A NLR of >4.86 predicted delirium. |
| Kowalska, K., et al., 2018 | Stroke, Poland | 21/144 | DSM 5 | Yes | Serum IL-6, TNFα, leucocyte count, monocytes. LPS induced TNF-α, IP-10, IL-1β, IL-6, IL-12, IL-10, IL-8 | Patients with delirium had significantly lower levels of LPS induced TNFα, IP-10, IL-1β, IL-6, and IL-12 release, compared to those without. IL-6 was associated with increased risk of delirium in multivariate analysis. |
| Kupiec, A., et al., 2020 | Elective Cardiac Surgery, Poland | 30/149 | CAM-ICU | No | PCT | Post-operative PCT was significantly higher in those with delirium compared with those without. |
| Lammers-Lietz, F., et al., 2022 | Mixed Surgery, Germany | BIO-COG: 125/504 PHYDELIO:6/32 | DSM-IV-TR/ DSM5 | No | IL-8 | Post-operative day one IL-8 levels significantly higher in patients with delirium compared to patients without in BIO-COG group. On post-operative day 7 IL-8 levels were significantly higher in patients with delirium compared to those without in the PHYDELIO group. |
| Lee, H. J., et al., 2011 | Orthopaedic Surgery, South Korea | 18/65 | DRS | No | CRP | CRP on post-operative day 1 and 3 significantly higher in those with delirium compared to those without. |
| Li, X., et al., 2022 | Orthopaedic Surgery, China | 60/184 | CAM | No | NLR, PLR, PWR, CRP | Postoperative NLR, PWR and PLR significantly higher in delirium. Change in NLR, PLR and CRP associated with post-operative delirium. In multi-variate regression greater change in CRP and NLR were associated with greater chance of delirium. |
| Li, Q-H., et al., 2019 | Emergency Orthopaedic Surgery, China | 67/186 | CAM | No | CRP, S100A12 | Postoperative S100A12 levels and CRP were associated with the risk of developing delirium. S100A12 independent. |
| Liu, P., et al., 2013 | Non-Cardiac Surgery, China | 50/338 | CAM-ICU | No | IL-6 | Post-operative IL-6 level higher in those with delirium compared to those without. |
| Lu, G. W., et al., 2020 | Breast Surgery – Oncology, China | 30/152 | CAM | No | Translocator Protein (TSPO) | Postoperative translocator protein levels independently associated with delirium. |
| Ma, X., et al., 2022 | Elective Orthopaedic Surgery, China | 44/143 | CAM | No | CRP | Post-op CRP significantly higher in those with POD compared to those without. Independent when controlling for confounders. |
| Macdonald, A., et al., 2007 | Geriatric Medicine, UK | 26/84 | CAM | Yes | CRP | No significant difference in CRP in prevalent delirium on admission. |
| McIntosh, T. K., et al., 1985 | Mixed Elective Surgery, US | 3/7 | DSM III | No | Cortisol | Postoperative delirium is associated with a prolonged rise in cortisol. |
| McKay, T. B., et al., 2022 | Cardiac surgery, US | 12/24 | CAM | No | IL-6, CCL20, CCL19, MCP-3 (CCL7) | Increase in IL-6 seen in both delirious and non-delirious cohort. Increase in MCP-3 seen in delirious cohort. Decrease in CCL19 and CCL20 seen in delirious cohort. |
| Mietani, K., et al., 2022b | Elective General Surgery, Oncology Japan | 15/96 | CAM-ICU | No | IL-6, PAI-1, PECAM-1, p-selectin, leucocyte count | Change in PAI-1 between pre-operatively and post-operative day 1 was significantly greater in those that developed delirium compared to those that did not. Change in p-selectin between post-operative day 2 and 3 was also significantly greater. |
| Mu, D. L., et al., 2010 | Cardiac surgery, China | 123/243 | CAM-ICU | No | Cortisol | Serum cortisol on post-operative day one significantly higher in those with delirium compared to those without. |
| Nagase, M., et al., 2012 | Palliative care - oncology, Japan | 49/115 | MDAS | Yes | CRP, leucocyte count | CRP significantly higher in those with delirium compared to those without. Remained significant in dendritic analysis. |
| Osse, R. J., et al., 2012 | Cardiac surgery, Netherlands | 63/125 | CAM-ICU | No | Neopterin, CRP | Post-operative neopterin significantly higher in those with delirium compared to those without. Post-operative neopterin >44nmol/l also independently associated. |
| Oyama, T., et al, 2022 | Elective General Surgery, Oncology Japan | 20/110 | ICDSC | No | NLR, PLR | Post-operative PLR or NLR not associated with delirium. |
| Pfister, D., et al., 2008 | Intensive care/sepsis, Switzerland | 12/16 | CAM-ICU | No | CRP, IL-6, Cortisol | CRP and cortisol significantly higher in those with sepsis associated delirium compared to those without. No difference in IL-6. |
| Plaschke, K., et al., 2013 | Non-cardiac surgery, Germany | 20/37 | CAM-ICU | Yes | IL-6 | No significant difference between IL-6 levels in those with delirium and those without. |
| Plaschke, K., et al., 2010 | Cardiac surgery, Germany | 32/114 | CAM-ICU | Yes | CRP, IL-6, leucocyte count, Cortisol | IL-6 and cortisol levels significantly higher in those with delirium compared to those without. |
| Pol, R. A., et al., 2014 | Vascular surgery, Netherlands | 16/277 | DSM IV | No | CRP | Raised post-operative CRP (>5mg/L) is independently associated with delirium. Patients with delirium had significantly higher CRP levels than those without delirium. |
| Ren, Q., et al., 2020 | Spinal surgery, China | 12/206 | CAM | No | CRP | Pots-operative CRP significantly higher in those with delirium compared to those without. Post -operative CRP independently predictive of delirium. |
| Ritchie, C. W., et al., 2014 | General Medicine, UK | 87/710 | CAM | No | CRP | CRP is associated with delirium in acute medical admissions, after controlling for confounders. |
| Sanchez, J. C., et al., 2021 | All hospitalised patients, Colombia | 27/115 | DSM IV | Yes | Leptin, leucocyte count | Leptin levels were significantly lower in those with delirium compared to those without. |
| Simons, K. S., et al., 2018 | Intensive care, Netherlands | 35/50 | CAM-ICU | No | IL-1β, IL-6, IL-10, TNF-α, MCP-1, neopterin, leucocyte count | There were no differences in the levels of any of the markers between those with delirium and those without. |
| Shi, C., et al., 2010 | Non cardiac surgery, China | 73/192 | Nu-DESC | No | Cortisol | Post-operative delirium is associated with an increased cortisol on post-operative day one. |
| Slor, C. J., et al., 2019 | Orthopaedic surgery, Netherlands | 41/121 | CAM | No | CRP | Higher CRP levels associated with delirium using GEE method, after controlling for confounders. |
| Soler-Sanchis, A., et al, 2022 | Emergency department - Geriatric, Spain | 128/256 | DSM 5 | Yes | CRP, leucocyte count, lymphocyte count, neutrophil count, monocyte count, NLR, PLR | No difference in CRP, leucocyte count, neutrophil count, lymphocyte count, monocyte count or lymph fraction between those with delirium and those without. Monocyte fraction significantly higher in delirium group. Neutrophil fraction, NLR and PLR significantly lower in delirium group. |
| Sun, L., et al., 2016 | Elective Maxillofacial Surgery - Oncology, China | 56/257 | CAM | No | PCT, Cortisol, IL-6, CRP | The peak levels of all inflammatory markers were higher in the delirium group than in the no delirium group. |
| Tanabe, S., et al., 2020 | Surgery, US | 22/70 | CAM | No | IL-1β, IL-2, IL-4, IL-6, IL-8, IL-10, IL-12, MCP-1 | IL-8, IL-10 and MCP-1 rose to higher levels between pre-op and post-op in delirium group compared to no delirium group. |
| Theologou, S., et al., 2018 | Cardiac surgery, Greece | 20/179 | CAM-ICU | No | CRP, NLR | NLR was significantly higher on postoperative day 2 in those with delirium compared to those without. This was an independent predictor of delirium. There was no difference in CRP or in NLR on postoperative day 1 |
| Tsuruta, R., et al., 2010 | Intensive Care, Japan | 21/103 | CAM-ICU | No | CRP | CRP significantly higher in those with delirium. |
| van den Boogaard, M., et al., 2011a | Intensive Care, Netherlands | 50/100 | CAM-ICU | Yes | CRP, PCT, Cortisol, IL-1β, IL-1RA, Il-6, IL-8, IL-10, IL-17, IL-18, TNF-α, MCP-1, MIF | TNF-α, IL-6, IL-8, MIF, IL-1RA, IL-10, MCP-1, PCT and Cortisol were significantly higher in those with delirium compared to those without. In multivariate regression analysis, IL-8 was independently associated with delirium in inflamed patients and IL-10 with delirium in noninflamed patients. |
| van den Boogaard, M., et al., 2011b | Intensive Care, Netherlands | 10/20 | CAM-ICU | Yes | CRP | CRP not significantly higher in those with delirium compared to those without. |
| van der Sluis, F. J., et al., 2017 | General Surgy - oncology, Netherlands | 45/436 | DSM-IV | No | CRP, leucocyte count | CRP is not associated with post-operative delirium. |
| Van Munster, B. C., et al., 2008 | Emergency orthopaedic, Netherlands | 50/98 | CAM | Yes | Il-6, IL-8, IL-12, TNF-α, IL1-β, IL-10 | IL-6 and IL-8 levels were significantly higher in delirium compared to those without delirium. Changes over time in IL-6 and IL-8 levels in patients with delirium differed significantly from changes in levels in patients without delirium. Less than 4% of TNFα, IL1-β, IL-10 were above the detection level. |
| van Munster, B. C., et al., 2010 | Emergency orthopaedic, Holland | 62/120 | CAM | Yes | IL-6, IL-8, Cortisol | IL-6 and IL-8 significantly higher in those with delirium compared to those without. In multivariable analysis cortisol and LogIL-6,  were significantly associated with delirium, but not after adjusting for pre-existing cognitive impairment. |
| Vasunilashorn, S. M., et al, 2019 | Elective non- cardiac surgery, US | 10/20 | CAM | No | Histidine-rich glycoprotein (HRG), Complement C2, complement component C9 (CO9), Complement C4-B (CO4B), Complement factor I (CFAI) 67 75 3 6, CRP, Complement factor D, Complement C4-A, Alpha-1-antichymotrypsin, alpha-1-acid glycoprotein | The model with IL-6, IL-2, and CRP was the “best combined model” postoperatively. |
| Vasunilashorn, S. M., et al., 2022 | Elective non- cardiac surgery, US | 18/36 | CAM | No | Chitinase-3-like protien 1, platelet factor 4, MHC class I polypeptide-related sequence A, resistin, CMRF35-like molecule 6 (CD300C), Programmed cell death 1 ligand 1, Low affinity immunoglobulin gamma Fc region receptor III-B, Tumor necrosis factor receptor superfamily member 1A, Complement C4b, CCL27, Tumor necrosis factor ligand superfamily member 9, Eotaxin. IL-25, IL-6, MHC class I polypeptide-related sequence A, CCL16, Plasma protease C1 inhibitor | Chitinase-3-like-protein-1 (CHI3L1/YKL-40) was identified as the sole delirium-associated protein in a postoperative predictor model. Multi-protein modelling found high postoperative IL-6 increased the risk of delirium. |
| Vasunilashorn, S. M., et al., 2015 | Elective non-cardiac surgery, US | Discovery: 39/78/Replication: 36/72, Pooled 75/150 | CAM | No | IL-1β, IL-2, IL-4, IL-5, IL-6, IL-8, IL-10, IL-12, IFN-ɣ, GMCSF, TNF-α | Post-operative day 2 levels of IL-2 and IL-6 were significantly higher in those with delirium compared to those without in the discovery cohort. In the replication cohort IL-5 and IL-6 were significantly higher. In the pooled cohort IL-2 and IL-6 were significantly higher. |
| Vasunilashorn, S. M., et al., 2017 | Elective non-cardiac surgery, US | 134/560 | CAM | No | CRP | On post-operative day 2 patients with CRP in the highest quartile were 1.5 times more likely to develop delirium. |
| Wanderlind, M. L. Z., et al., 2020 | Intensive care, Brazil | 47/94 | CAM-ICU | Yes | IL-1β | IL-1b significantly higher on day 2 in those with delirium compared to those without. |
| Wang, B., et al., 2022 | Emergency orthopaedic surgery, China | 22/44 | CAM | No | microRNA-320, IGF-1 | No difference in postoperative plasma IGF-1 in delirium and no delirium groups. MicroRNA320 upregulated in postoperatively in delirium group compared to no delirium group and IGF-1 mRNA downregulated. |
| Wang, C, G., at al., 2018 | Orthopaedic surgery, China | 59/306 | CAM | No | Leucocyte count | Postoperative leucocyte count was a significant risk factor for post operative delirium, however, did not remain significant in multivariate analysis. |
| Wu, J. G., et al., 2022 | Surgery, US | 24/64 | CAM | No | IL-18 | Non-delirious patients had similar changes in plasma IL-18 to delirious patients. |
| Xiang, D., et al., 2017 | Elective general surgery - oncology, China | 39/160 | CAM-ICU | Yes | CRP | CRP on post-operative day 2 was significantly higher in those with delirium. Not significant after controlling for confounders. |
| Zhang, W., et al., 2022c | Orthopaedic surgery, China | 13/53 | CAM | No | IL-1β, IL-6, TNF-α | No difference in IL-6, IL-1β, and TNF-α on postoperative day 1 between those that developed delirium and those that did not. On POD3 and POD7 IL-1β higher in delirium patients. On postoperative day 3, but not 7, TNF-α higher in POD patients. |
| Zhang, L. N., et al., 2016a | Intensive care (Sepsis), China | 29/59 | CAM-ICU | Yes | CRP, PCT, IL-1β, IL-6, TNF-α, leucocyte count, TRAF 6, S100A8 | IL‑1β, TNF‑α, IL‑6, S100A8 and TRAF 6 significantly higher in sepsis associated encephalopathy group compared to sepsis alone group. |

*IL-1β- interleukin-1β, IL-1ra – Interleukin-1 receptor agonist, IL-6 – Interleukin-6, TNF-α – Tumour necrosis factor-α, IFN-γ – Interferon gamma, LIF – leukaemia inhibitory factor, IL-10 – Interleukin-10, IL-8 – Interleukin-8, IL-2- Interleukin-2, IL-4 – Interleukin-4, IL-12 – Interleukin-12, MCP-1 – Monocyte chemoattractant protein-1, sIL-2R – soluble interleukin-2 receptor, CRP- C-reactive protein, ESR- erythrocyte sedimentation rate, I score – inflammatory score, MPO – myeloperoxidase, NLR – neutrophil to lymphocyte ratio, PWR – platelet to white blood cell ratio, PLR – Platelet lymphocyte ratio, PNR – platelet to neutrophil ratio, IP-10 - Interferon gamma-induced protein 10, MCP-3 – monocyte chemoattractant protein- 3, PAI-1 – Plasminogen activator inhibitor-1, IL-17 – Interleukin-17, MIP-1β- Macrophage inflammatory protein-1β, PCT – Procalcitonin, IL-18 – Interleukin-18, MIF – Macrophage migration inhibitory factor, GM-CSF – Granulocyte macrophage colony stimulating factor, IGF-1 – insulin-like growth factor-1, IL-15 – Interleukin-15, TRAF 6 - TNF Receptor Associated factor 6.*

Table 7 Records included in the qualitative synthesis that measured peripheral immune response in a mixed population of prevalent and incident delirium

| **Author and Date** | **Setting** | **Number with Delirium/Sample Size** | **Diagnostic Criteria** | **Measure of Peripheral Inflammatory Response** | **Key Findings** |
| --- | --- | --- | --- | --- | --- |
| Adamis, D., et al., 2020 | Geriatric Medicine, Ireland | 40/198 | CAM | IGF-1 | Low levels of IGF-I were independently associated with the occurrence of any delirium during the hospitalisation of medically ill older people. |
| Adamis, D., et al., 2009 | Geriatric Medicine, UK | 28/67 | CAM | IL-1α, IL-1β, IL-1RA, IL-6, TNF-α, IFN-γ, LIF, IGF-I | Low levels of IGF-I and IL-1RA were associated with delirium. but no association was found between delirium and any other -pro-inflammatory factors studied. High IFN-γ and low IGF-I had significant effects on delirium severity. |
| Alexander, S. A., et al., 2014 | Intensive Care, US | 35/77 | CAM-ICU | Il-6, Il-10, IL-8 | Mean IL-6 levels significantly higher in patients with delirium compared to those without delirium. Mean IL-8 and IL-10 levels did not differ. |
| Cape, E., et al., 2014 | Emergency Orthopaedic Surgery, UK | 9/23.0 | CAM | IL-1β, IL-1RA, IGF-1, IFN-ɣ | IL-1β, IL-1ra or IGF-1 were not significantly higher in those with delirium at any point compared to those that never developed delirium. IFN-ɣ not analysed as all values were below the level of detection. |
| Egberts, A. and Mattace-Rraso, F. U. S., 2017 | Geriatric Medicine, Netherlands | 23/86 | DSM IV/DSM 5 | CRP, leucocyte count, neutrophil count, lymphocyte count, neutrophil fraction, lymphocyte fraction, NLR | NLR and neutrophil fraction were higher and lymph fraction lower in patients with incident and prevalent delirium compared to those without. No difference in other inflammatory markers. |
| Egberts, A., et al., 2015 | Geriatric Medicine, Netherlands | 23/86 | DSM IV | Neopterin, IGF-1, IL-6, CRP | Levels of IL-6 and neopterin were higher and IGF-1 lower in those with incident and prevalent delirium than those without. |
| Forget, M. F., et al., 2021 | COVID-19, Canada | 62/127 | DSM 5 | CRP | Mean CRP value over the first 3 days was associated with a higher risk of delirium. |
| Foroughan, M., et al., 2016 | Geriatric Medicine, Iran | 44/200 | DSM IV-TR | CRP, ESR | Higher ESR level and positive CRP results significantly associated with delirium. |
| Hall, R. J., et al., 2016 | Emergency Orthopaedic Surgery, UK/Norway | 64/137 | CAM | Neopterin | Neopterin levels higher in those with delirium at any time, compared to those without delirium. |
| Neerland, B. E., et al., 2016 | Emergency Orthopaedic Surgery, UK/Norway | 37/149 | CAM | CRP, IL-6, IL-6R | No difference in serum CRP, IL-6 or IL-6R levels between those with incident or prevalent delirium and those without delirium. |
| Reznik, M. E., et al., 2022 | Stroke, US | 157/284 | DSM 5 | Leucocyte count, NLR | Higher NLR and leucocyte count associated with incident and prevalent delirium. High NLR associated with delirium after controlling for confounders. |
| Skrede, K., et al., 2015 | Emergency Orthopaedic Surgery, Norway | (5 delirium pre-op, 6 delirious post op)/19 | CAM | MCP-1 | No statistically significant difference in pre-operative MCP-1 between patients with and without preoperative delirium. A rise in MCP-1 from pre to post op was associated with delirium and this was statistically significant. |
| Thisayakorn, P., et al., 2022 | Orthopaedic surgery, Thailand | 19/65 | CAM-ICU | IL-2, IL-5, IL-10, IL-12 (p70), IL-13, IL-15, GM-CSF, immune profiles: IRS/CIRS, M1, T cell growth) | More than 20% of all measured concentrations of IL-2, IL-5, IL-10, IL-12, IL-13, IL-15 and GM-CSF were below the detection limit and therefore excluded from analysis of single cytokines. IRS/CIRS ratio, M1, T17, T cell growth, and NLR were significantly and positively associated with delirium at any point. In multivariable analysis IL-8 was independently predictive of delirium on day 1, IL-4 was independently predictive on day 2 and TH1 profile and sIL-1RA also on day 2 in separate regression and IRS/CIRS ratio at day 2 in another regression. Mean DRS explained by IL-6 on day 1. |
| Thisayakorn, P., et al., 2021 | Orthopaedic surgery, Thailand | 19/65 | CAM-ICU | Leucocyte count, neutrophil fraction, lymphocyte fraction, NLR z score, monocyte fraction, eosinophil fraction, basophil fraction | Neutrophil fraction and NLR z score significantly higher in those with delirium at any point compared to those without. Lymphocyte fraction significantly lower. |

*IGF-1 – insulin-like growth factor-1, IL-1α – Interleukin-1α, IL-1β- interleukin-1β, IL-1ra – Interleukin-1 receptor agonist, IL-6 – Interleukin-6, TNF-α – Tumour necrosis factor-α, IFN-γ – Interferon gamma, LIF – leukaemia inhibitory factor, IL-10 – Interleukin-10, IL-8 – Interleukin-8, CRP- C-reactive protein, NLR – Neutrophil to lymphocyte ratio, ESR – Erythrocyte sedimentation rate, IL-6R – Interleukin-6 receptor, MCP-1 – Monocyte chemoattractant protein-1, IL-2- Interleukin-2, IL-5 – Interleukin-5, IL-15 – interleukin-15, GM-CSF – Granulocyte macrophage colony stimulating factor.*

## Demographic Tables

Table 8 Number and Percentage of Records Investigating Each Clinical Population

| **Population** | | **Preceding Delirium (n=94)** | **During Delirium (n=87)** | **Mixed Prevalent and Incident Delirium (n=15)** |
| --- | --- | --- | --- | --- |
| **Surgery** | **Elective Surgery** | 20 | 18 | 0 |
|  | **Emergency Surgery** | 8 | 7 | 3 |
|  | **Mixed Surgery** | 29 | 27 | 4 |
|  | **Surgery – Oncology** | 12 | 8 | 0 |
| **Medicine** | **Stroke** | 5 | 4 | 1 |
|  | **Medicine** | 6 | 4 | 0 |
|  | **Medicine - Oncology** | 0 | 2 | 0 |
|  | **Geriatric Medicine** | 1 | 3 | 5 |
| **Intensive Care** | **Intensive Care** | 12 | 13 | 1 |
| **Mixed** | **All Hospitalised Patients** | 0 | 1 | 0 |
|  | **All Hospitalised Patients not ITU** | 1 | 1 | 0 |

Table 9 Diagnostic tools used and number and percentage of studies that used each one

| **Diagnostic Tool** | **Preceding Delirium (n=94)** | **During Delirium (n=82)** | **Mixed Prevalent and Incident Delirium (n=15)** |
| --- | --- | --- | --- |
| **CAM** | 36 | 34 | 6 |
| **CAM-ICU** | 31 | 35 | 3 |
| **DSM 5** | 8 | 3 | 4 |
| **DSM IV** | 10 | 6 | 2 |
| **DMS III** | 0 | 2 | 0 |
| **DOS** | 1 | 0 | 1 |
| **NuDESC** | 1 | 1 | 0 |
| **DRS** | 2 | 2 | 0 |
| **ICDS** | 5 | 4 | 0 |
| **MDAS** | 0 | 1 | 0 |

## Supplementary Qualitative Synthesis

### Acute Phase Proteins (APPs)

APPs are produced in the liver in response to pro-inflammatory cytokines. Seven APPs were identified.

##### C-Reactive Protein (CRP)

CRP was most measured with 79 records included. CRP is a protein produced by the liver that increases with inflammation. 47 records measured CRP preceding delirium and 45 were included in the qualitative synthesis. 19/45 (42.2%) studies reported that preceding delirium CRP was significantly higher in those that developed delirium compared to those that did not (Dillon et al., 2017; Girard et al., 2012; Guenther et al., 2013; Guo et al., 2016; Hindiskere et al., 2020; Knaak et al., 2019; Kotfis, Ślozowska, et al., 2019; Lechowicz et al., 2021; Miao et al., 2018; Osse et al., 2012; Pasqui et al., 2022; Pol R.A. et al., 2011; Sakaguchi T. et al., 2018; Shen et al., 2016; Vasunilashorn et al., 2019; Xiang et al., 2017; Xu et al., 2019; L. Zhang et al., 2022; Z. Zhang et al., 2014). This was confirmed to be independent of confounders in 6/45 (13.3%) studies(Girard et al., 2012; Guo et al., 2016; Hindiskere et al., 2020; Knaak et al., 2019; Xiang et al., 2017; Z. Zhang et al., 2014). 5/45 (11.1%) studies reported that a “high” CRP was associated with the development of delirium(Kim et al., 2016; Macdonald et al., 2007; McManus et al., 2009; Nydahl et al., 2017; Vasunilashorn et al., 2017). 20/44 (44.4%) reported no association between CRP and delirium(Alvarez-Perez F.J., 2017; Baranyi, 2014; Brattinga et al., 2022; Cerejeira et al., 2012, 2013; Chung K.S. et al., 2015; De Castro et al., 2014; Dittrich T. et al., 2016; Eshmawey et al., 2019; Guldolf et al., 2021; Jiang et al., 2020; Khan et al., 2022; Lee et al., 2011; Lemstra et al., 2008; Mietani, Hasegawa-Moriyama, Yagi, Inoue, Ogata, Shimojo, et al., 2022; Ren et al., 2020; Slor et al., 2019; Sun et al., 2016; Theologou et al., 2018; Watts et al., 2007). 48 records measured CRP during delirium and 44 were included in qualitative synthesis. 24/43 (55.8%) reported significantly higher levels of CRP in patients with delirium (Burkhart C.S. et al., 2010; Cereghetti C. et al., 2017; Cizginer S. et al., 2017; Dillon et al., 2017; Hasegawa et al., 2015; John M. et al., 2017; Khan et al., 2022; Kotfis, Bott-Olejnik, Szylińska, Listewnik, et al., 2019; Kotfis, Bott-Olejnik, Szylińska, & Rotter, 2019; Lee et al., 2011; Q.-H. Li et al., 2019; Ma et al., 2022; Nagase et al., 2012; Osse et al., 2012; Pfister et al., 2008; Pol et al., 2014, 2014; Ren et al., 2020; Ritchie et al., 2014; Slor et al., 2019; Sun et al., 2016; Tsuruta et al., 2010; Vasunilashorn et al., 2019; Xiang et al., 2017). 11/43 (25.6%) confirmed the association after controlling for confounders(Burkhart C.S. et al., 2010; Cereghetti C. et al., 2017; Kotfis, Bott-Olejnik, Szylińska, Listewnik, et al., 2019; X. Li et al., 2022; Ma et al., 2022; Nagase et al., 2012; Pol et al., 2014; Ren et al., 2020; Ritchie et al., 2014; Slor et al., 2019; Tsuruta et al., 2010). One study reported that patients with a CRP level in the top quartile following elective surgery were more likely to have delirium(Vasunilashorn et al., 2017). Belooseskey et al. reported a difference in the CRP kinetic curve in patients with delirium following orthopaedic surgery compared to those without delirium(Beloosesky et al., 2004, 2007). There was a greater increase in CRP following surgery and sustained higher levels in the delirium group compared to the no delirium group until to 30 days post-op. One study reported a significantly greater change in CRP between pre-op and following orthopaedic surgery in those with delirium compared to those without(X. Li et al., 2022). 16/43 (37.2%) records found no association between CRP and delirium(Baranyi, 2014; Cerejeira et al., 2013, 2013; de Rooij et al., 2007; Dittrich T. et al., 2016; Erikson et al., 2019; Knaak et al., 2019; Kotfis, Ślozowska, et al., 2019; Macdonald et al., 2007; Plaschke et al., 2010; Soler-Sanchis et al., 2022; Theologou et al., 2018; van den Boogaard et al., 2011; van den Boogaard M. et al., 2011; van der Sluis F.J. et al., 2017; Zhang L.-N. et al., 2016). Six records were identified that measured CRP and combined prevalent and incident delirium (Egberts et al., 2015; Egberts & Mattace-Raso, 2017; Forget et al., 2021; Foroughan M. et al., 2016; Neerland et al., 2016; Visser L. et al., 2015). 3/6 (50%) reported a positive association between CRP and delirium(Forget et al., 2021; Foroughan M. et al., 2016; Visser L. et al., 2015). 3/6 (50%) reported no associations

##### Other Acute Phase Proteins

Three records measured Plasminogen activator inhibitor-1 (PAI-1) preceding delirium with two records reporting results from the same dataset. 2/3 (67.7%) reported PAI-1 was significantly higher preoperatively in those that developed delirium compared to those that did not (Mietani, Hasegawa-Moriyama, Yagi, Inoue, Ogata, Kurano, et al., 2022; Mietani, Hasegawa-Moriyama, Yagi, Inoue, Ogata, Shimojo, et al., 2022). 1/3 (33.3%) reported no association(Girard et al., 2012). Two records measured PAI-1 during delirium and found a significantly greater rise in PAI-1 between pre-op and post-operative day 1 in those with delirium. All remaining APPs were measured in one record, both preceding and during delirium (Vasunilashorn et al., 2019). This study then combined APPs to develop a multi protein model for delirium. A model with pre-operative raised CRP and reduced AZGP1 predicted delirium at post-operative day 2(Vasunilashorn et al., 2019).

### Cytokines

Cytokines are small proteins with an important role in immune cell signalling and a variety have been studies in delirium.

##### Interleukin-6 (IL-6)

The most commonly measured was interleukin-6 (IL-6), a pro-inflammatory cytokine. 25 records measured IL-6 preceding delirium. 24 were included in the qualitative synthesis with results from 23 datasets. 6/24 (25%) reported significantly higher IL-6 in those who developed delirium compared to those who did not(Brattinga et al., 2022; Capri et al., 2014; Lv et al., 2021; Miao et al., 2018; Peng et al., 2019; Shen et al., 2016). 2/24 (8.3%) reported the logIL-6 was significantly higher in those that developed delirium(Mietani, Hasegawa-Moriyama, Yagi, Inoue, Ogata, Kurano, et al., 2022; Mietani, Hasegawa-Moriyama, Yagi, Inoue, Ogata, Shimojo, et al., 2022). 16/24 (66.6%) records reported no association(Ballweg et al., 2021; Cerejeira et al., 2012, 2013; CheheiliSobbi et al., 2021; Y. Chen et al., 2019; Lemstra et al., 2008; Liu et al., 2013; Ritter et al., 2014; Rudolph et al., 2008; Simons et al., 2018; Sun et al., 2016; Vasunilashorn et al., 2015, 2021; S. Zhang et al., 2022; W. Zhang et al., 2022, 2022; Zhang Z.-Y. et al., 2016). 27 records measured IL-6 during delirium. 22 records were included in the qualitative synthesis, reporting results from 20 datasets. 11/22 (50%) records reported significantly higher IL-6 levels in delirium(Y. Chen et al., 2019; Erikson et al., 2019; Kowalska et al., 2018; Liu et al., 2013; McKay et al., 2022; Plaschke et al., 2010; Sun et al., 2016; van den Boogaard et al., 2011; Van Munster et al., 2008; Vasunilashorn et al., 2015; Zhang L.-N. et al., 2016) and 1/22 (4.5%) reported significantly higher levels in one cohort(Plaschke K., 2013). 3/22 (13.6%) also reported an association between IL-6 and delirium. Van Munster et al. reported that logIL-6 was significantly higher in the delirium group(van Munster B.C. et al., 2010) and De Rooij et al. reported significantly more patients with IL-6 levels above the detection level in the delirium group(de Rooij et al., 2007). Beloosesky et al. reported a significant difference in the kinetic curve of IL-6 in those with delirium compared to those without(Beloosesky et al., 2007). 7/22 (31.8%) reported no association between IL-6 and delirium(Adamis et al., 2007; Cerejeira et al., 2012, 2013; Mietani, Hasegawa-Moriyama, Yagi, Inoue, Ogata, Shimojo, et al., 2022; Pfister et al., 2008; Simons et al., 2018; W. Zhang et al., 2022). Four records measured IL-6 in a mixed population. 2/4 (50%) reported significantly higher levels of IL-6 in those with delirium at any point compared to those who never had delirium(Alexander et al., 2014; Egberts et al., 2015). 2/4 (50%) reported no association(Adamis et al., 2009; Neerland et al., 2016).

##### Tumour Necrosis Factor Alpha (TNF-α)

13 records measured the pro-inflammatory cytokine Tumour necrosis factor alpha (TNF-α) preceding delirium. 2/13 (15.4%) reported an association between TNF-α and delirium. Peng et al found pre-operative TNF-α was significantly higher in those that developed delirium compared to those that did not(Peng et al., 2019). Ritter et al found that TNF-α on admission to ITU was significantly higher in those that developed delirium(Ritter et al., 2014). 11/13 (84.6%) records found no association(Ballweg et al., 2021; Cerejeira et al., 2013; Khan et al., 2022; Kozak et al., 2017; Rudolph et al., 2008; Simons et al., 2018; Vasunilashorn et al., 2015; W. Zhang et al., 2022; Zhang Z.-Y. et al., 2016). 20 records measured TNF-α during delirium. 17 were included in the qualitative analysis with results from 15 datasets. 5/20 (25%) reported significantly higher TNF-α levels in patients with delirium compared to those without(Kazmierski, Banys, Latek, Bourke, & Jaszewski, 2014; Kazmierski, Banys, Latek, Bourke, Jaszewski, et al., 2014; van den Boogaard et al., 2011; Zhang L.-N. et al., 2016; W. Zhang et al., 2022). 12/20 (60%) records found no association (Adamis et al., 2007; Ballweg et al., 2021; Beloosesky et al., 2007; Brum et al., 2015; Cerejeira et al., 2012, 2013; Erikson et al., 2019; Khan et al., 2022; Kowalska et al., 2018; Simons et al., 2018; Van Munster et al., 2008; Vasunilashorn et al., 2015), with one reporting <4% above the detection level(Van Munster et al., 2008).

##### Interleukin-1 Beta (IL-1β)

Nine records were identified that measured Interleukin-1 Beta (IL-1β) preceding delirium. 2/9 (22.2%) records found an association. Vasunilashorn et al. reported a that IL-1β levels were higher in those that developed postoperative delirium compared to those that did not in the discovery cohort but results did not remain in the pooled cohort(Vasunilashorn et al., 2015). Wanderlind et al. found IL-1β levels were higher on admission to ITU in patients who developed delirium compared to those that did not(Wanderlind et al., 2020). 7/9 (77.7%) records found no association(Brattinga et al., 2022; Kozak et al., 2017; Ritter et al., 2014; Rudolph et al., 2008; Simons et al., 2018; W. Zhang et al., 2022; Zhang Z.-Y. et al., 2016). 13 records were identified that measured IL-1β during delirium and 11 were included in the qualitative synthesis. 3/11 (27.3%) reported an association. Wanderlind et al. and Zhang et al. both reported IL-1β was significantly higher in intensive care patients with delirium compared to those without(Wanderlind et al., 2020; Zhang L.-N. et al., 2016). Ballweg et al. reported a significantly greater change in IL-1β between pre-op and post-operative day 1 in those with delirium compared to those without(Ballweg et al., 2021). Another study reported IL-1β was significantly higher in patients with delirium on post-operative day 3, but not post-operative day 1(W. Zhang et al., 2022). 8/11 (72.7%) reported no association(Adamis et al., 2007; Beloosesky et al., 2007; de Rooij et al., 2007; Simons et al., 2018; Tanabe et al., 2020; van den Boogaard et al., 2011; Van Munster et al., 2008; Vasunilashorn et al., 2015), including one that reported less than 4% of values were above the detection level(Van Munster et al., 2008). Two records were identified in a mixed population of incident and prevalent delirium and reported no association between delirium and IL-1β(Adamis et al., 2009; Cape et al., 2014).

##### Interleukin-10 (IL-10)

Ten records measured interleukin-10 (IL-10) preceding delirium with results presented from nine datasets. All studies reported no association between Il-10 and delirium(Ballweg et al., 2021; Brattinga et al., 2022; Capri et al., 2014; Cerejeira et al., 2012, 2013; Khan et al., 2022; Ritter et al., 2014; Rudolph et al., 2008; Simons et al., 2018; Vasunilashorn et al., 2015). 15 records measured IL-10 during delirium. 10 were included in the qualitative synthesis and reported results from nine datasets. 3/10 (30%) records reported a positive association between delirium and IL-10. Van den Boogaard et al. reported that IL-10 was significantly higher in intensive care patients with delirium, and this was independent of confounders in the subgroup of patients without infection(van den Boogaard et al., 2011). Ballweg et al. and Tanabe et al. reported results from the same dataset and described a greater change in IL-10 between pre-op and postoperative day 1 in those with delirium(Ballweg et al., 2021; Tanabe et al., 2020). 7/10 (70%) found no association(Beloosesky et al., 2004, 2007; Cerejeira et al., 2012, 2013; de Rooij et al., 2007; Simons et al., 2018; Van Munster et al., 2008), with one not analysing results as less than 4% were above the detection limit. One record measured IL-10 in a mixed population of incident and prevalent delirium but did not analyse results as >20% of samples were below the detection limit(Thisayakorn et al., 2022).

##### Interleukin-2 (IL-2)

Five records measured IL-2 preceding delirium, presenting results from 4 datasets. 2/5 (40%) reported significantly higher IL-2 in those that developed delirium(Ballweg et al., 2021; Vasunilashorn et al., 2015). 3/5 (60%) reported no association(Kazmierski, Banys, Latek, Bourke, & Jaszewski, 2014; Kazmierski et al., 2013; Rudolph et al., 2008). Six records measured IL-2 during delirium, presenting data from four datasets. 4/6 (66.7%) reported significantly higher IL-2 levels in those with delirium compared to those without(Kazmierski, Banys, Latek, Bourke, & Jaszewski, 2014; Kazmierski, Banys, Latek, Bourke, Jaszewski, et al., 2014; Kazmierski et al., 2013; Vasunilashorn et al., 2015) and 1/6 (16.7%) reported a significantly greater change in IL-2 in those with delirium compared to those without(Ballweg et al., 2021). Tanabe et al. presented results from a subpopulation of the same dataset but did not report a significant change in IL-2(Tanabe et al., 2020).

##### Other Cytokines

Two studies measured Neutrophil gelatinase-associated lipocalin (NGAL). Girard et al. reported no difference in levels of NGAL on admission to ITU in those that developed delirium compared to those that did not(Girard et al., 2012). Brattinga et al. reported no difference in pre-operative NGAL (Brattinga et al., 2022). Van den Boogard et al reported significantly higher Macrophage Migration Inhibitory Factor (MIF) in those with delirium(van den Boogaard et al., 2011). Zhang et al. reported significantly high TNF Receptor Associated Factor 6 (TRAF-6) and S100A8 in patients with sepsis associated encephalitis(Zhang L.-N. et al., 2016). Li et al. reported that S100A12 was independently associated with delirium(Q.-H. Li et al., 2019). Xu et al. reported that pre-operative Soluble fibrinogen like protein 2 (sFGL2) was an independent predictor of delirium(Xu et al., 2019). No association was reported between delirium and interleukin-4 (IL-4)(Ballweg et al., 2021; Rudolph et al., 2008; Vasunilashorn et al., 2015), interleukin-12 (IL-12)(Ballweg et al., 2021; Rudolph et al., 2008; Thisayakorn et al., 2022; Van Munster et al., 2008; Vasunilashorn et al., 2015), interferon gamma (IFN-γ)(Adamis et al., 2007, 2009; Cape et al., 2014; Rudolph et al., 2008; Vasunilashorn et al., 2015), interleukin-17 (IL-17)(Mao et al., 2022; Rudolph et al., 2008; van den Boogaard et al., 2011), interleukin-5 (IL-5)(Rudolph et al., 2008; Thisayakorn et al., 2022), interleukin-15 (IL-15)(Rudolph et al., 2008; Thisayakorn et al., 2022), interleukin-18 (IL-18)(van den Boogaard et al., 2011), interleukin-1 alpha (IL-1α)(Adamis et al., 2007, 2009), interleukin-7 (IL-7)(Rudolph et al., 2008), leukaemia inhibitory factor (LIF)(Adamis et al., 2007, 2009), granulocyte and monocyte colony stimulating factor (GM-CSF)(Rudolph et al., 2008; Thisayakorn et al., 2022), interleukin-25 (IL-25)(Vasunilashorn et al., 2021), interleukin-33 (IL-33)(Mao et al., 2022) and inflammatory score(Capri et al., 2014; Cerejeira et al., 2012, 2013). One study assessed multiple cytokines together as “immune profiles” in a mixed population. Thisayakorn et al. reported IRS/CIRS, M1 macrophage, Th1, Th17, T cell growth immune profiles were significantly higher in delirium than no delirium(Thisayakorn et al., 2022).

### Chemokines

Chemokines are a group of cytokines who trigger cell migration, particularly of leucocytes.

##### Interleukin-8 (IL-8)

Interluekin-8 (IL-8)/CXCL8 was the most measured chemokine with 10 records reporting results. Eight studies measured IL-8 preceding delirium but this only included results from seven datasets. Only 1/8 (12.5%) reported positive results. Lammers-Lietz reported results from two cohorts of patients and in one found IL-8 was significantly higher in those that developed delirium(Lammers-Lietz et al., 2022). 7/8 (87.5%) reported no association(Ballweg et al., 2021; Capri et al., 2014; Cerejeira et al., 2012, 2013; Khan et al., 2022; Rudolph et al., 2008; Vasunilashorn et al., 2015; Zhang Z.-Y. et al., 2016). 14 records measured IL-8 during delirium. 11 were included in the qualitative synthesis which reported data from 8 datasets. 4/11 (36.4%) reported significantly higher IL-8 in patients with delirium compared to those without(Lammers-Lietz et al., 2022; van den Boogaard et al., 2011; Van Munster et al., 2008; van Munster B.C. et al., 2010). One study confirmed this to be independent of confounders in the subgroup of patients with infection(van den Boogaard et al., 2011). 3/11 (27.3%) reported a greater change in IL-8 in those with delirium but results were all from the same dataset (Ballweg et al., 2021; Casey et al., 2020; Tanabe et al., 2020). 1/11 (9.1%) reported a significant difference in the IL-8 kinetic curve(Beloosesky et al., 2007). One record reported more IL-8 levels were over the detection limit in the delirium group(de Rooij et al., 2007). 3/11 (27.3%) reported no association(Cerejeira et al., 2012, 2013; Khan et al., 2022).

##### Monocyte Chemoattractant-1 (MCP-1)/CCL2

Seven studies measured monocyte chemoattractant-1 (MCP-1)/CCL2 and qualitative results were available for six. Three studies measured MCP-1 preceding delirium. Rudolph et al. reported higher pre-operative MCP-1 levels in those that developed delirium(Rudolph et al., 2008). However, Ballweg et al. found no difference in levels preoperatively(Ballweg et al., 2021) and Simons et al. reported no difference in MCP-1 levels on admission to intensive care in those that developed delirium(Simons et al., 2018). Three records measured MCP-1 during delirium, although two reported results from the same dataset. Van den Boogaard reported MCP-1 was higher in patients with delirium in intensive care compared to those without delirium(van den Boogaard et al., 2011). Ballweg et al. and Tanabe et al. reported a greater rise in MCP-1 between pre-op and postoperative day 1 in those with delirium compared to those without(Ballweg et al., 2021; Tanabe et al., 2020). One study measured delirium in a mixed population of prevalent and incident delirium. Skrede at al. reported no difference in preoperative MCP-1 in those with hip fracture and prevalent delirium on admission and incident delirium following surgery(Skrede et al., 2015). However, they did report a greater change in MCP-1 between pre-op and post-op in the delirium group.

##### Other Chemokines

A range of other chemokines were measured in 4 studies and qualitative results available for 3. However no associations between the remaining chemokines and delirium were found(Rudolph et al., 2008; Thisayakorn et al., 2022; Vasunilashorn et al., 2021).

### Cell Receptors, Surface Markers and Adhesion Molecules

##### Cell Receptors

Cell receptors are important in immune cell communication and their soluble form can be measured. Soluble interleukin-1 receptor antagonist (sIL-1RA) was measured in 9 records. 7 records were included in the qualitative synthesis with data from 6 datasets (Adamis et al., 2007, 2009; Ballweg et al., 2021; Beloosesky et al., 2007; Cape et al., 2014; Rudolph et al., 2008; van den Boogaard et al., 2011). Two records measured sIL-1RA preceding delirium (Ballweg et al., 2021; Rudolph et al., 2008). 1/2 (50%) reported that sIL-1RA was significantly lower preoperatively in patients that developed delirium compared to those that did not(Ballweg et al., 2021). Five records measured sIL-1RA during delirium(Adamis et al., 2007; Ballweg et al., 2021; Beloosesky et al., 2007; Tanabe et al., 2020; van den Boogaard et al., 2011) and 1/5 (20%) reported sIL-1RA was significantly higher in intensive care patients with delirium compared to those without delirium(van den Boogaard et al., 2011). 1/4 (25%) reported a greater change in sIL-1RA levels between pre-op and post-operative day 1 in those with delirium(Ballweg et al., 2021). 3/5 (60%) reported no association(Adamis et al., 2007; Beloosesky et al., 2007; Tanabe et al., 2020). Two studies measured sIL-1RA in a mixed population of incident and prevalent delirium(Adamis et al., 2009; Cape et al., 2014). 1/2 (50%) found IL-1RA was significantly lower in the delirium group compared to the no delirium group(Adamis et al., 2009). Three records were identified that measured TNF receptors preceding delirium(Girard et al., 2012; Ritter et al., 2014; Rudolph et al., 2008). 2/3 (66%) reported significantly higher soluble tumour necrosis factor receptor 1 (sTNFR1) levels in those that developed delirium compared to those that did not(Girard et al., 2012; Ritter et al., 2014). In one study this result remained after controlling for confounders(Girard et al., 2012). 1/2 (50%) reported significantly higher soluble tumour necrosis factor 2 (sTNFR2) levels in those that developed delirium compared to those that did not(Ritter et al., 2014). Soluble interleukin-2 receptor (sIL-2R) was measured in two records(Baranyi, 2014; Rudolph et al., 2008). Baranyi et al. reported significantly higher sIL-2R levels on postoperative day 2 in patients with delirium lasting >1 day compared to those without delirium(Baranyi, 2014) however Rudolph et al. found no difference in sIL-2R levels pre-operatively(Rudolph et al., 2008). Translocator protein, which is a benzodiazepine receptor, was found to be significantly higher in patients with delirium post-operatively compared to those without(Lu et al., 2020). No other receptors measured were found to be associated with delirium(Neerland et al., 2016; Rudolph et al., 2008; Vasunilashorn et al., 2021).

##### Cell Surface Molecules

Cell surface molecules are important in immune cell communication. Vasunilashorn et al. found no association between any cell surface molecules measured and delirium(Vasunilashorn et al., 2021).

##### Cell Adhesion Molecules

Cell adhesion molecules have an important role in immune cell migration to sites of inflammation. Three records measured cell adhesion molecules(Mao et al., 2022; Mietani, Hasegawa-Moriyama, Yagi, Inoue, Ogata, Kurano, et al., 2022; Mietani, Hasegawa-Moriyama, Yagi, Inoue, Ogata, Shimojo, et al., 2022), although two of these reported results from the same dataset. No difference was found in P-selectin or PECAM-1 preceding delirium(Mietani, Hasegawa-Moriyama, Yagi, Inoue, Ogata, Kurano, et al., 2022; Mietani, Hasegawa-Moriyama, Yagi, Inoue, Ogata, Shimojo, et al., 2022). During delirium the change in p-selectin between post-operative day 1-2 and 3-2 was significantly greater in those with delirium compared to those without(Mietani, Hasegawa-Moriyama, Yagi, Inoue, Ogata, Shimojo, et al., 2022). Preceding delirium Mao et al. reported no difference in VCAM-1 or e-selectin between those that developed delirium and those that did not(Mao et al., 2022).

### Immune Cells and Complement

Many studies compared immune cell counts or fractions in patients with and without delirium.

##### Leucocyte Count and Fraction

25 records measured leucocyte count preceding delirium and 24 were included in the qualitative synthesis. 8/24 (33.3%) reported significantly higher leucocyte count in those that developed delirium compared to those that did not (Alvarez-Perez F.J., 2017; De Castro et al., 2014; Guo et al., 2016; Kotfis, Ślozowska, et al., 2019; Lechowicz et al., 2021; Lv et al., 2021; Pasqui et al., 2022; Xu et al., 2019). 1/24 (4.2%) reported significantly lower leucocyte count(Feng et al., 2019). 2/24 (8.3%) reported significantly more patients in the delirium group had a “high” leucocyte count(Kim et al., 2016; Watts et al., 2007). 13/24 (54.2%) records reported no association(J. Chen et al., 2022; Chu et al., 2016; Dittrich T. et al., 2016; Guenther et al., 2013; Hindiskere et al., 2020; Jiang et al., 2020; Kozak et al., 2017; Mietani, Hasegawa-Moriyama, Yagi, Inoue, Ogata, Kurano, et al., 2022; Peng et al., 2019; Pol R.A. et al., 2011; Sakaguchi T. et al., 2018; Q. Shi et al., 2019; L. Zhang et al., 2022). 14 records measured leucocyte count during delirium. 12 records were included in the qualitative synthesis and presented results from 11 datasets. 5/12 (41.7%) records reported significantly higher leucocyte count in delirium(Kotfis, Bott-Olejnik, Szylińska, Listewnik, et al., 2019; Kotfis, Bott-Olejnik, Szylińska, & Rotter, 2019; Kowalska et al., 2018; Nagase et al., 2012; C.-G. Wang et al., 2018) and 1/12 (8.3%) reported a that a “high” leucocyte count was predictive of delirium(van der Sluis F.J. et al., 2017). 6/12 (50%) reported no association(Dittrich T. et al., 2016; Kotfis, Ślozowska, et al., 2019; Plaschke et al., 2010; Sánchez et al., 2013; Soler-Sanchis et al., 2022; Zhang L.-N. et al., 2016). Three records measured leucocyte count in a mixed population. 1/3 (33.3%) reported a significantly higher leucocyte count in those with delirium at any point(Reznik et al., 2021). 2/3 (66.7%) reported no association(Egberts & Mattace-Raso, 2017; Thisayakorn et al., 2021).

##### Neutrophil Count and Fraction

11 records measured neutrophil count or fraction preceding delirium and 10 were included in the qualitative synthesis. 7/10 (70%) reported significantly higher neutrophil counts or percentage of neutrophils in patients that developed delirium(Guldolf et al., 2021; Guo et al., 2016; He et al., 2020; Kotfis, Ślozowska, et al., 2019; Lechowicz et al., 2021; X. Li et al., 2022; Pasqui et al., 2022). 3/10 (30%) reported no association between neutrophils and delirium(Feng et al., 2019; Jiang et al., 2020; Watts et al., 2007). Five records were identified that measured neutrophil count during delirium. Four were included in the qualitative analysis with results from three databases. Kotfis et al. reported significantly higher neutrophil count in patients with post-stroke delirium in two studies presenting results from one dataset(Kotfis, Bott-Olejnik, Szylińska, Listewnik, et al., 2019; Kotfis, Bott-Olejnik, Szylińska, & Rotter, 2019). However 2/4 records (50%) reported no association between neutrophils and delirium(Kotfis, Ślozowska, et al., 2019; Soler-Sanchis et al., 2022). Two records measured neutrophil fraction in a mixed delirium population and both reported an association(Egberts & Mattace-Raso, 2017; Thisayakorn et al., 2021).

##### Lymphocyte Count or Fraction

Nine records measured lymphocyte count or fraction preceding delirium and 8 were included in the qualitative synthesis. 2/8 (25%) records reported lower lymphocyte counts in those that developed delirium(Guldolf et al., 2021; Pasqui et al., 2022). 6/8 (75%) records reported no association between lymphocytes and delirium(Feng et al., 2019; He et al., 2020; Jiang et al., 2020; Kotfis, Ślozowska, et al., 2019; Lechowicz et al., 2021; L. Zhang et al., 2022). Five records measured lymphocytes during delirium. Four were included in the qualitative synthesis from three datasets. 2/4 (50%) reported significantly lower lymphocytes in post-stroke delirium(Kotfis, Bott-Olejnik, Szylińska, Listewnik, et al., 2019; Kotfis, Bott-Olejnik, Szylińska, & Rotter, 2019) and ¼ (25%) reported significantly higher lymphocytes on postoperative day 3 and 5, but not 1, in those with delirium following cardiac surgery(Kotfis, Ślozowska, et al., 2019). ¼ (25%) reported no association between lymphocytes and delirium(Soler-Sanchis et al., 2022). Two records measured lymphocytes in a mixed population and both reported lymphocyte count was significantly lower in those with delirium at any point(Egberts & Mattace-Raso, 2017; Thisayakorn et al., 2021).

##### Monocyte Count and Fraction

Three records measured monocyte count. Kowalska et al. found a significantly higher monocyte in those with delirium compared to those without(Kowalska et al., 2018). In contrast Soler-Sanchis et al. reported no association however they did find higher monocyte fraction in those with delirium compared to those without(Soler-Sanchis et al., 2022). Thisayakorn et al. reported no difference in monocyte count in a mixed population of delirium(Thisayakorn et al., 2021). They also measured basophils in a mixed population and found no association(Thisayakorn et al., 2021).

##### Immune Cell Function

A small number of studies measured immune cell function. Two records assessed NK cell activity. Hatta et al. reported no difference in NK cell function on admission inpatients that developed delirium compared to those that did not(Hatta et al., 2014). However they did find a significantly greater increase in NK cell activity between day 1 and 2 of admission in those developing delirium compared to those without(Hatta et al., 2014). In contrast Nakamura et al. reported no difference in NK cell activity in cardiac surgery patients with delirium compared to those without(Nakamura et al., 2001). Two records assessed monocyte cytokine production. Hatta et al. reported no difference in monocyte production of IL-1β on admission to ITU in those that developed delirium compared to those that did not (Hatta et al., 2014). They also reported no difference in the change in mean IL-1β production between day 1 and day 2 of admission in those developing delirium compared to those without(Hatta et al., 2014). In contrast Kowalska et al. reported reduced monocyte release of TNFα, IP-10, IL-1β, IL-6, and IL-12(Kowalska et al., 2018). CheheiliSobbi et al. measured cytokine production by leucocytes in whole blood but reported no difference in levels of IL-6, IL-8, IL-10 and TNF- α preceding or during delirium(CheheiliSobbi et al., 2021).

##### Immune Cell Ratios

Many studies calculated ratios based on immune cells. 16 records measured NLR preceding delirium and 15 were included in the qualitative synthesis. 10/15 reported significantly higher NLR in those that developed delirium compared to those that did not(J. Chen et al., 2022; Fernández-Jiménez et al., 2021; Guldolf et al., 2021; He et al., 2020; Kinoshita et al., 2021; X. Li et al., 2022; Oyama et al., 2022; Pasqui et al., 2022; Yang et al., 2022; Zhao et al., 2021). 5/15 records reported no association(Jiang et al., 2020; Kotfis, Ślozowska, et al., 2019; Lechowicz et al., 2021; Theologou et al., 2018; L. Zhang et al., 2022). Eight records measured NLR during delirium. Seven were included in the qualitative synthesis with results from six databases. 3/7 (42.9%) records reported significantly higher NLR in those with delirium compared to those without(Kotfis, Bott-Olejnik, Szylińska, Listewnik, et al., 2019; Kotfis, Bott-Olejnik, Szylińska, & Rotter, 2019; X. Li et al., 2022). 2/7 (28.6%) reported significantly higher NLR in those with delirium on post-operative day 3 and 5 but not on postoperative day 1(Kotfis, Ślozowska, et al., 2019; Theologou et al., 2018). 1/7 (14.3%) reported a significantly lower NLR in delirium(Soler-Sanchis et al., 2022) and 1/6 (20%) reported no association(Oyama et al., 2022). Three records measured NLR in a mixed delirium population. All reported a significantly higher NLR in delirium at any point(Egberts et al., 2015; Reznik et al., 2021; Theologou et al., 2018) and this was independent of confounders in one study.(Reznik et al., 2021) Seven records measured PLR preceding delirium. 2/7 (28.6%) reported a significantly lower PLR(Kotfis, Ślozowska, et al., 2019; X. Li et al., 2022), and 2/7 (28.6%) a significantly higher PLR (Jiang et al., 2020; Pasqui et al., 2022) in those that developed delirium compared to those that did not. 3/7 (42.9%) reported no association(Lechowicz et al., 2021; Oyama et al., 2022; Yang et al., 2022). Three records measured PLR during delirium. Soler-Sanchis et al. reported a significantly lower PLR in delirium. In contrasts Li et al. reported a significantly higher postoperative PLR, and a significantly higher rise in PLR between pre-op and post-op in those with delirium compared to those without(X. Li et al., 2022). Oyama et al. reported no association(Oyama et al., 2022). Two records measured PWR preceding delirium and both reported significantly lower PWR in those that developed delirium compared to those that did not(Kotfis, Ślozowska, et al., 2019; X. Li et al., 2022). Four records measured PWR during delirium and all reported lower PWR in those with delirium compared to those without(Kotfis, Bott-Olejnik, Szylińska, Listewnik, et al., 2019; Kotfis, Ślozowska, et al., 2019; Lechowicz et al., 2021; X. Li et al., 2022). One study measured MLR preceding delirium and reported higher levels in those that developed delirium compared to those that did not(Yang et al., 2022). One record measured PNR during delirium and reported that PNR was lower in the delirium group(Kotfis, Bott-Olejnik, Szylińska, Listewnik, et al., 2019). Two studies measured the Systemic Immune-Inflammation Index preceding delirium and both reported higher levels in those that developed delirium compared to those that did not(Pasqui et al., 2022; Song et al., 2022).

##### Complement

Three studies measured complement, three preceding delirium(Mao et al., 2022; Vasunilashorn et al., 2019, 2021) and two during(Vasunilashorn et al., 2019, 2021). No association was found with any complement components measured.

### Hormones

Some hormones have an immunomodulating role.

##### Cortisol

Ten records measured cortisol preceding delirium. Eight were included in qualitative synthesis with data from eight datasets. 3/8 (37.5%) found cortisol was significantly higher in those that develop delirium compared to those that did not(Colkesen et al., 2013; Kazmierski, Banys, Latek, Bourke, Jaszewski, et al., 2014; Kazmierski et al., 2013). 3/8 (50%) confirmed this to be independent after adjusting for confounders. 5/8 (62.5%) reported no association(Avila-Funes et al., 2015; Cerejeira et al., 2013; Deiner et al., 2014; Eshmawey et al., 2019; Sun et al., 2016). 14 studies measured cortisol during delirium, presenting data from 12 datasets. 12/14 (85.7%) reported that cortisol was significantly higher in those with delirium compared to those without(Bisschop et al., 2011; Cerejeira et al., 2013; Kazmierski, Banys, Latek, Bourke, Jaszewski, et al., 2014; Kazmierski et al., 2013; Mu et al., 2010; Pfister et al., 2008; Plaschke et al., 2010; C. Shi et al., 2010; Sun et al., 2016; van den Boogaard et al., 2011; van Munster B.C. et al., 2010) and 1/14 (7.1%) reported delirium was associated with a prolonged rise in cortisol(McIntosh et al., 1985). 1/14 (7.1%) reported no association(Fassbender et al., 1994).

##### Insulin-Like Growth Factor-1 (IGF-1)

Ten records measured Insulin-like growth factor-1 (IGF-1) preceding delirium. 3/10 (30%) reported a significantly lower IGF-1 in those that developed delirium(Miao et al., 2018; Shen et al., 2016; Wilson et al., 2005), IGF-1 was an independent predictor in one study(Wilson et al., 2005). 7/10 (70%) found no association(Cerejeira et al., 2013; Chu et al., 2016; Khan et al., 2022, 2022; Lemstra et al., 2008; Morandi et al., 2011; Yen et al., 2016). Four records measured IGF-1 during delirium. 1/3 (25%) reported significantly lower IGF-1 levels in patients with delirium(Adamis et al., 2007). 3/4 (75%) reported no association(Cerejeira et al., 2013; Khan et al., 2022; B. Wang et al., 2022). Three records measured IGF-1 in a mixed population of delirium. 2/3 (66.7%) reported significantly lower IGF-1 levels in those with delirium at any point to those without(Adamis et al., 2020; Egberts et al., 2015). 1/3 (33.3%) found no association(Cape et al., 2014).

##### Leptin

Two records measured leptin, an adipokine with hormonal function associated with energy balance that rises with inflammation but has also been shown to be neuroprotective, preceding delirium. Li et al. found leptin on ITU admission was significantly lower in patients that developed delirium(G. Li et al., 2017). Chen et al found leptin was significantly lower pre-operatively in those that developed delirium(X.-W. Chen et al., 2014). One record assessed leptin during delirium and reported significantly lower levels in patients with delirium(Sánchez et al., 2013).

### Other Inflammatory Markers

##### Procalcitonin (PCT)

Three studies measured Procalcitonin (PCT), a precursor to the hormone calcitonin which rises with inflammation, preceding delirium. 2/3 (66.7%) reported no association(Lv et al., 2021; Sun et al., 2016). 1/3 (33.3%) reported that delirium was associated with a PCT level above the reference range(Kupiec et al., 2020). Four studies measured PCT during delirium. 2/4 (50%) reported PCT was significantly higher in those with delirium(Kupiec et al., 2020; van den Boogaard et al., 2011). ¼ (25%) reported the peak level of PCT was significantly higher in the delirium group(Sun et al., 2016). ¼ (25%) found no association(Zhang L.-N. et al., 2016).

##### Erythrocyte Sedimentation Rate (ESR)

Three studies measured erythrocyte sedimentation rate (ESR) preceding delirium, all found no association(Alvarez-Perez F.J., 2017; Chung K.S. et al., 2015; Guo et al., 2016). One study measured ESR during delirium and found no association(Beloosesky et al., 2004). One study measured ESR in a mixed population and reported a “higher” ESR was associated with delirium(Foroughan M. et al., 2016).

##### Other Inflammatory Markers

Three studies measured neopterin preceding delirium. 2/3 (66.7%) reported higher levels of neopterin in those that developed delirium which was independent of confounders(Miao et al., 2018; Osse et al., 2012). 1/3 (33.3%) found no association(Simons et al., 2018). Two studies measured neopterin during delirium. ½ (50%) reported significantly higher levels in those with delirium whilst another reported no association(Osse et al., 2012; Simons et al., 2018). Two studies measured neopterin in a mixed population and both reported significantly higher levels in those with delirium at any point compared to those without(Egberts et al., 2015; Hall et al., 2016). Two studies measured myeloperoxidase (MPO), an enzyme associated with neutrophil bacterial killing preceding delirium. Kazmierski et al. reported significantly higher MPO in delirium(Kaźmierski et al., n.d.) and Girard et al. reported no association(Girard et al., 2012). Kazmierski et al. also measured MPO during delirium and reported higher MPO levels were independently associated with delirium(Kaźmierski et al., n.d.). Vasunilashorn et al. measured CHI3L1 which was associated as the sole predictor protein in both a pre-operative and post-operative model(Vasunilashorn et al., 2021). One study measured micro-RNA-320 (B. Wang et al., 2022), mRNAs are small non-coding RNAs that regulate gene expression, preceding delirium. Wang et al. reported microRNA-320 was upregulated(B. Wang et al., 2022) preoperatively in patients that developed delirium compared to those that did not.

##
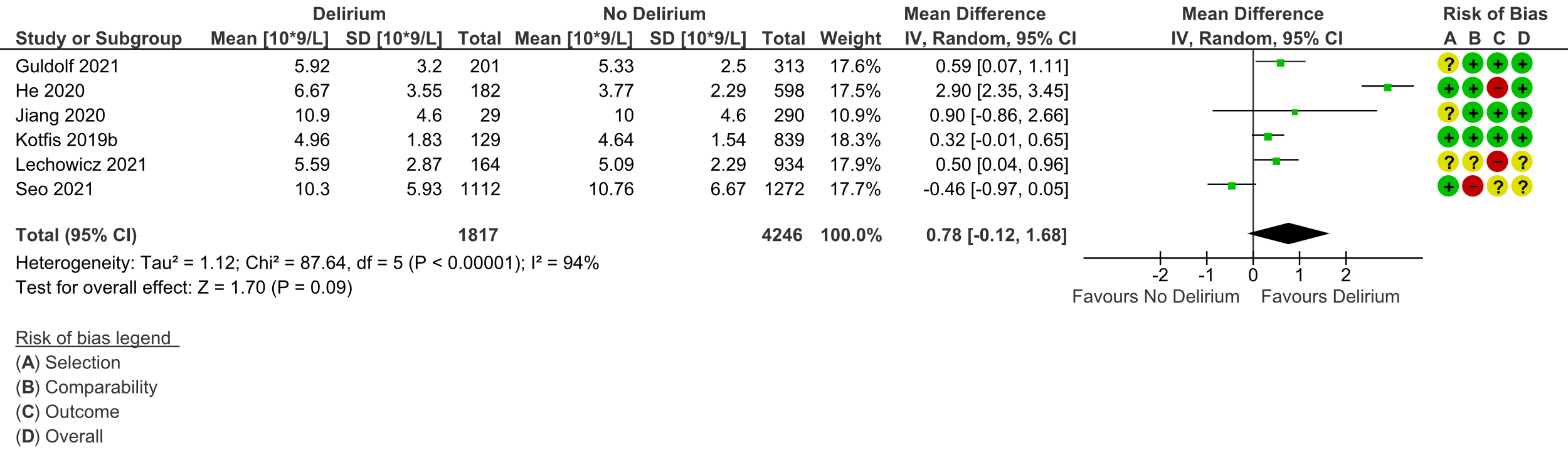
Supplementary Forest Plots of Inflammatory Markers Measured Preceding Delirium

Figure 3) Forest plots of meta-analysis of records measuring Lymphocyte Count (10^9^/L) preceding delirium, using random effects model. Mean difference (MD) and 95% CI (confidence interval) in participants that did and did not develop delirium. The green squares represent the mean difference for each study and the size of the square represents the weight of the study. The black lines represent the 95% CI. The black diamonds represent the overall MD.


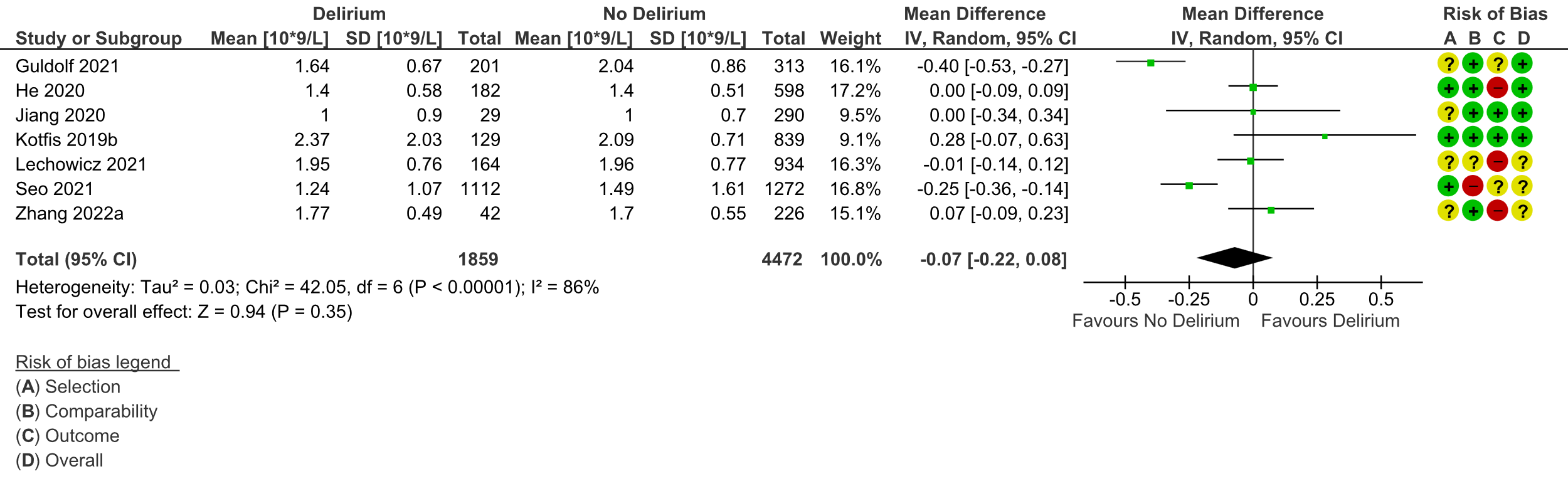

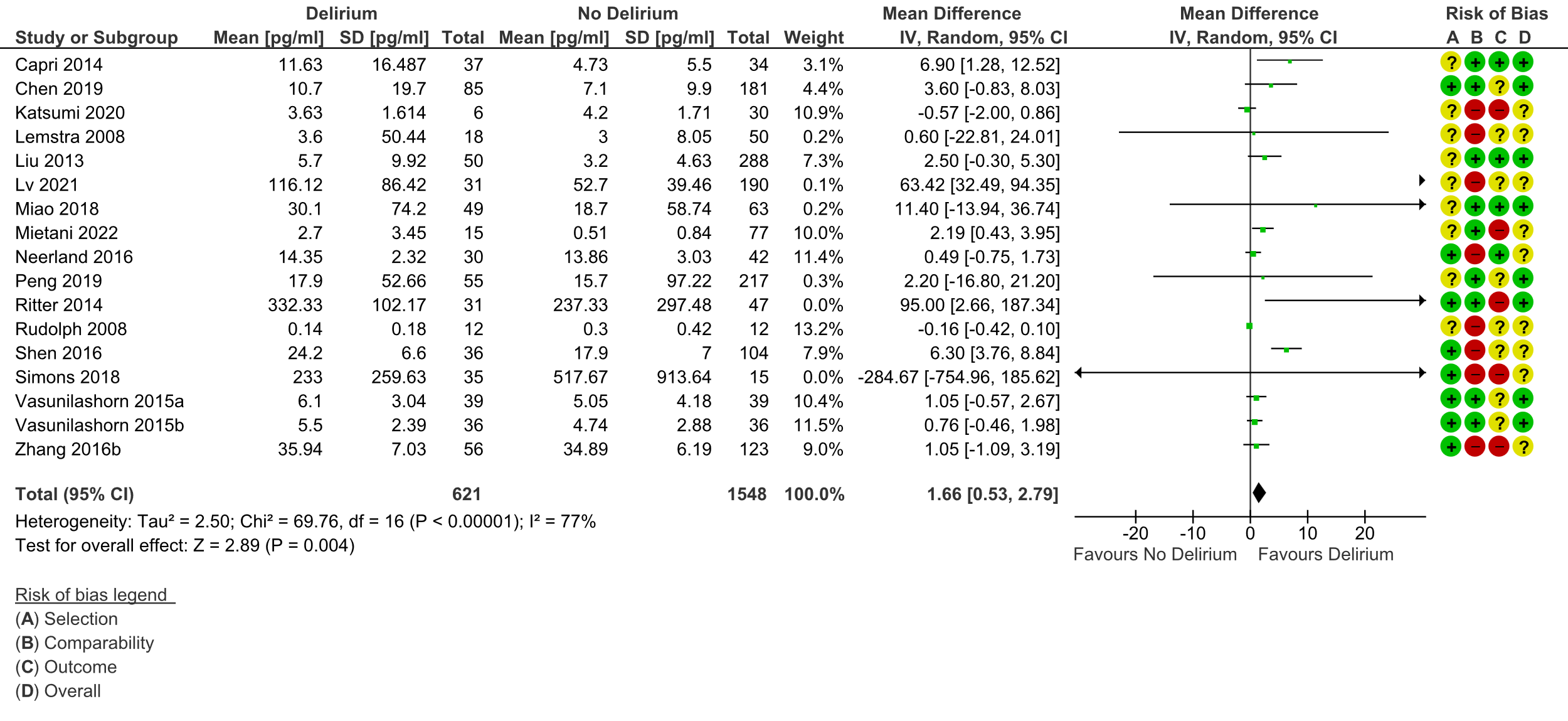


Low risk

Medium risk

High risk


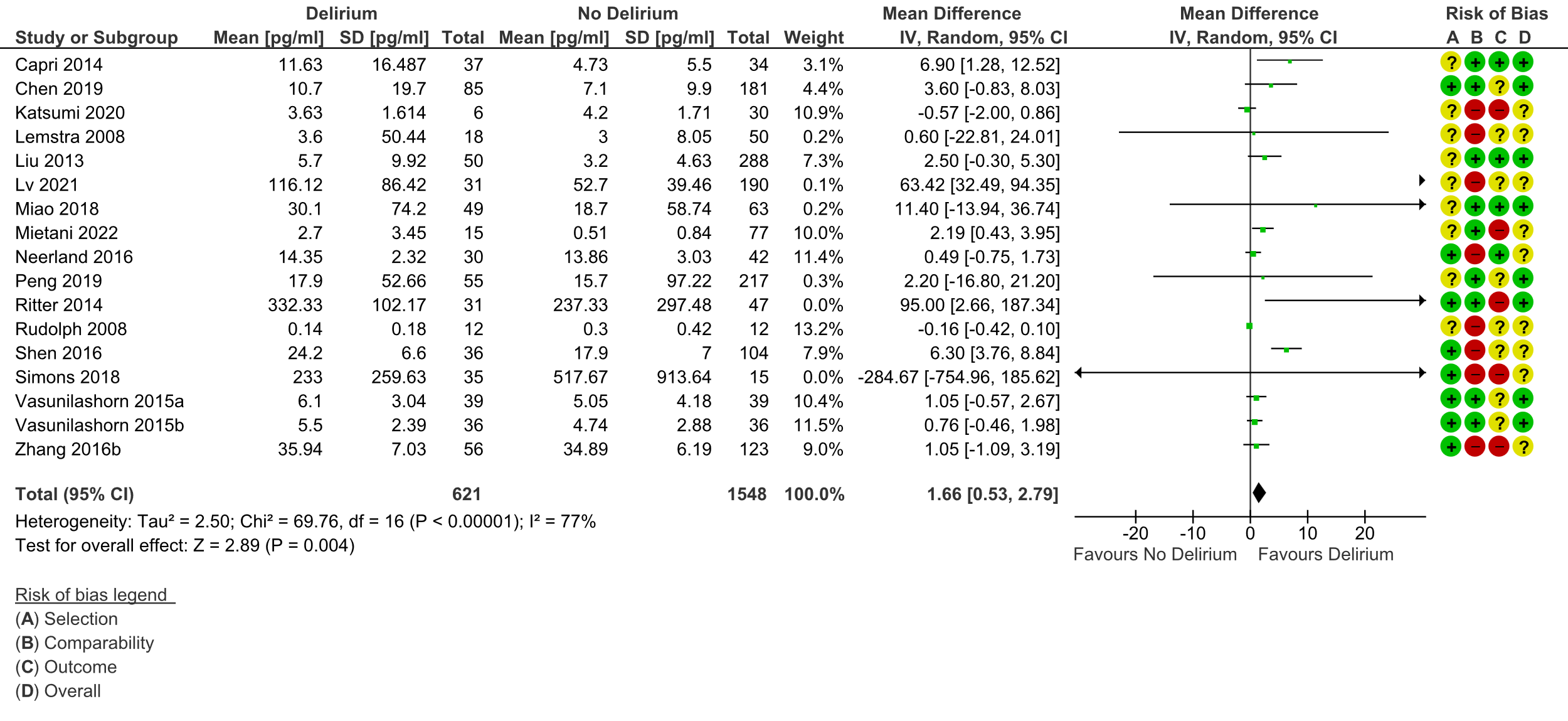


Low risk

Medium risk

High risk


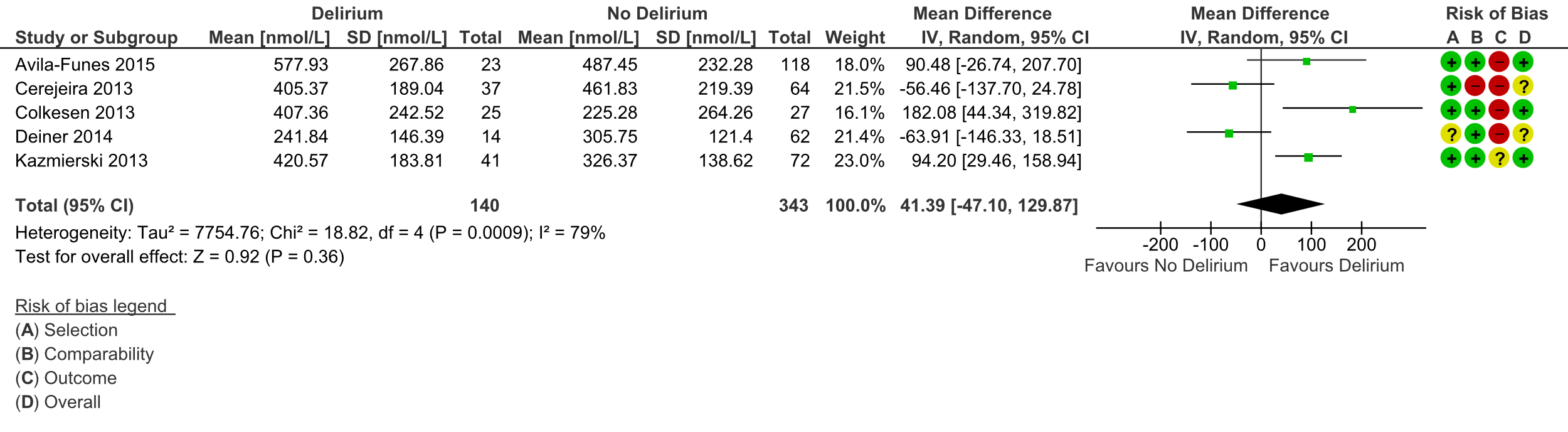

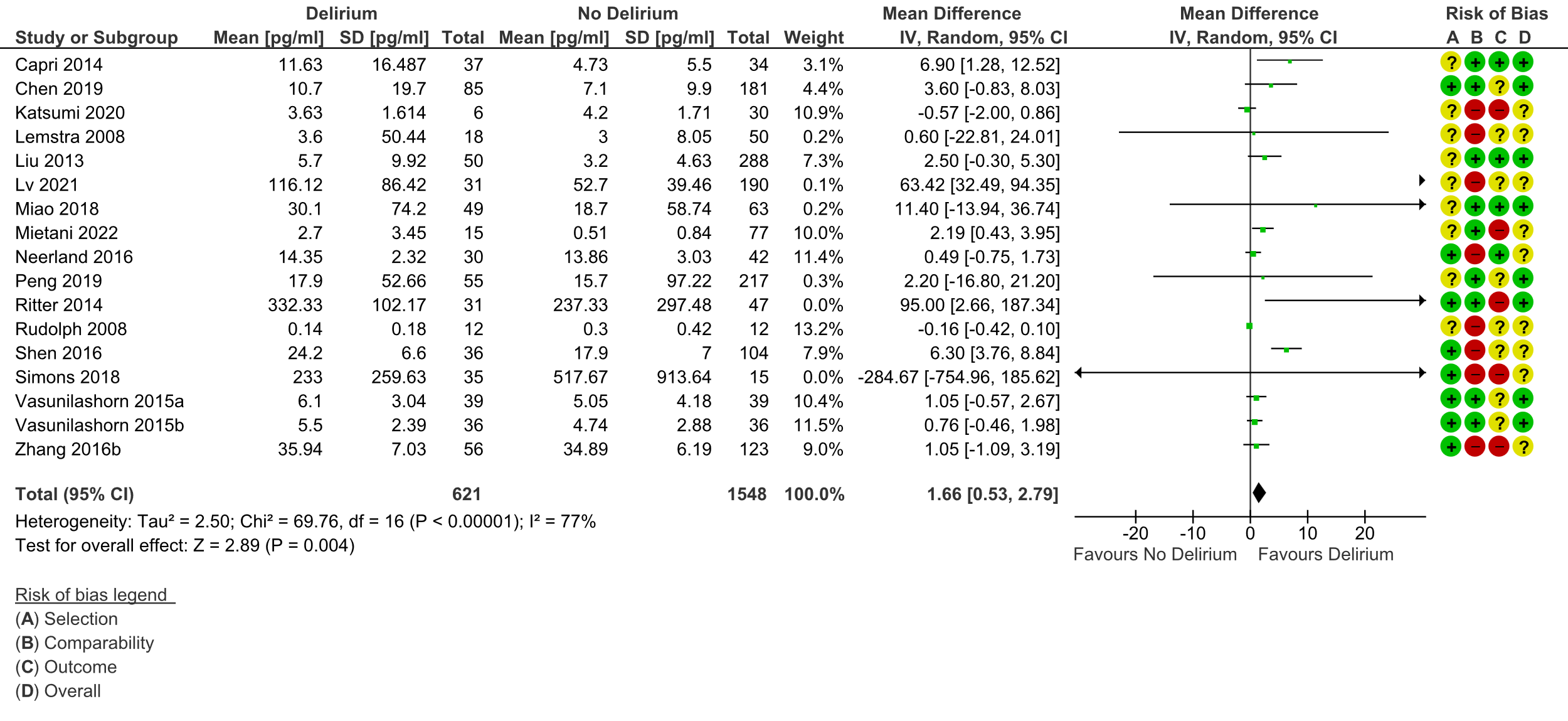


Low risk

Medium risk

High risk

Figure 1) Forest plot of meta-analysis of records measuring Cortisol (nmol/L) preceding delirium, using random effects model. Mean difference (MD) and 95% CI (confidence interval) in participants that did and did not develop delirium. The green squares represent the mean difference for each study and the size of the square represents the weight of the study. The black lines represent the 95% CI. The black diamonds represent the overall MD.

Figure 2) Forest plots of meta-analysis of records measuring Neutrophil Count (10^9^/L) preceding delirium, using random effects model. Mean difference (MD) and 95% CI (confidence interval) in participants that did and did not develop delirium. The green squares represent the mean difference for each study and the size of the square represents the weight of the study. The black lines represent the 95% CI. The black diamonds represent the overall MD.


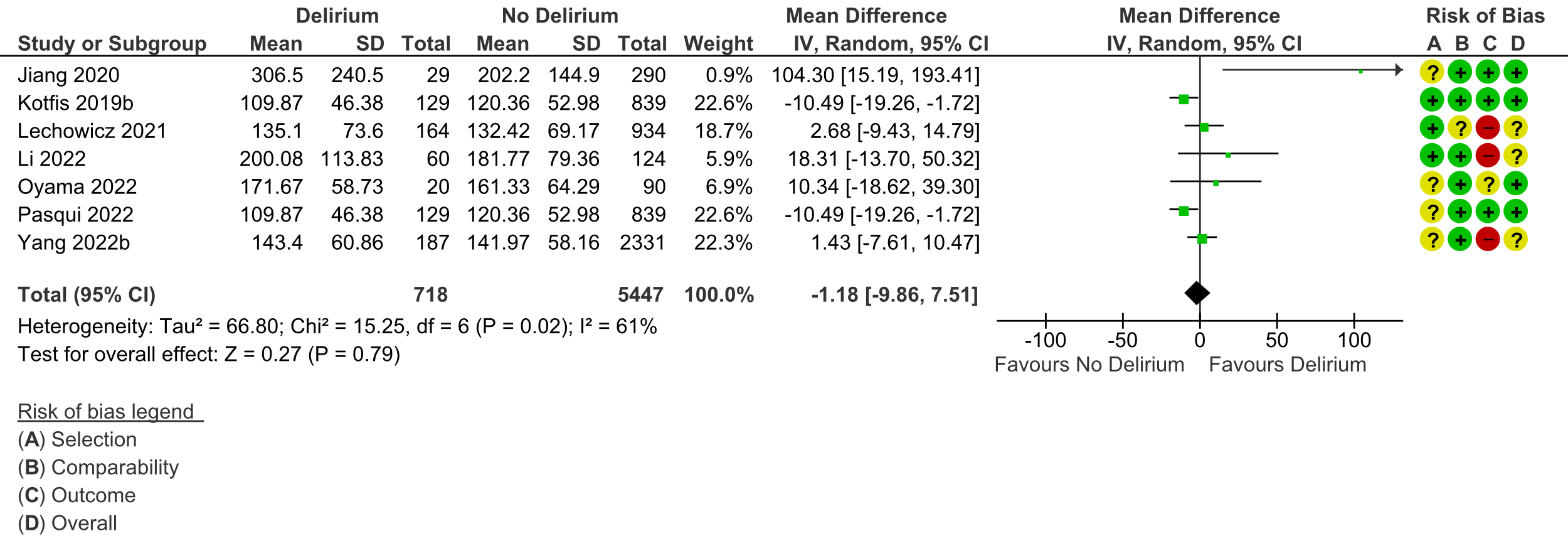

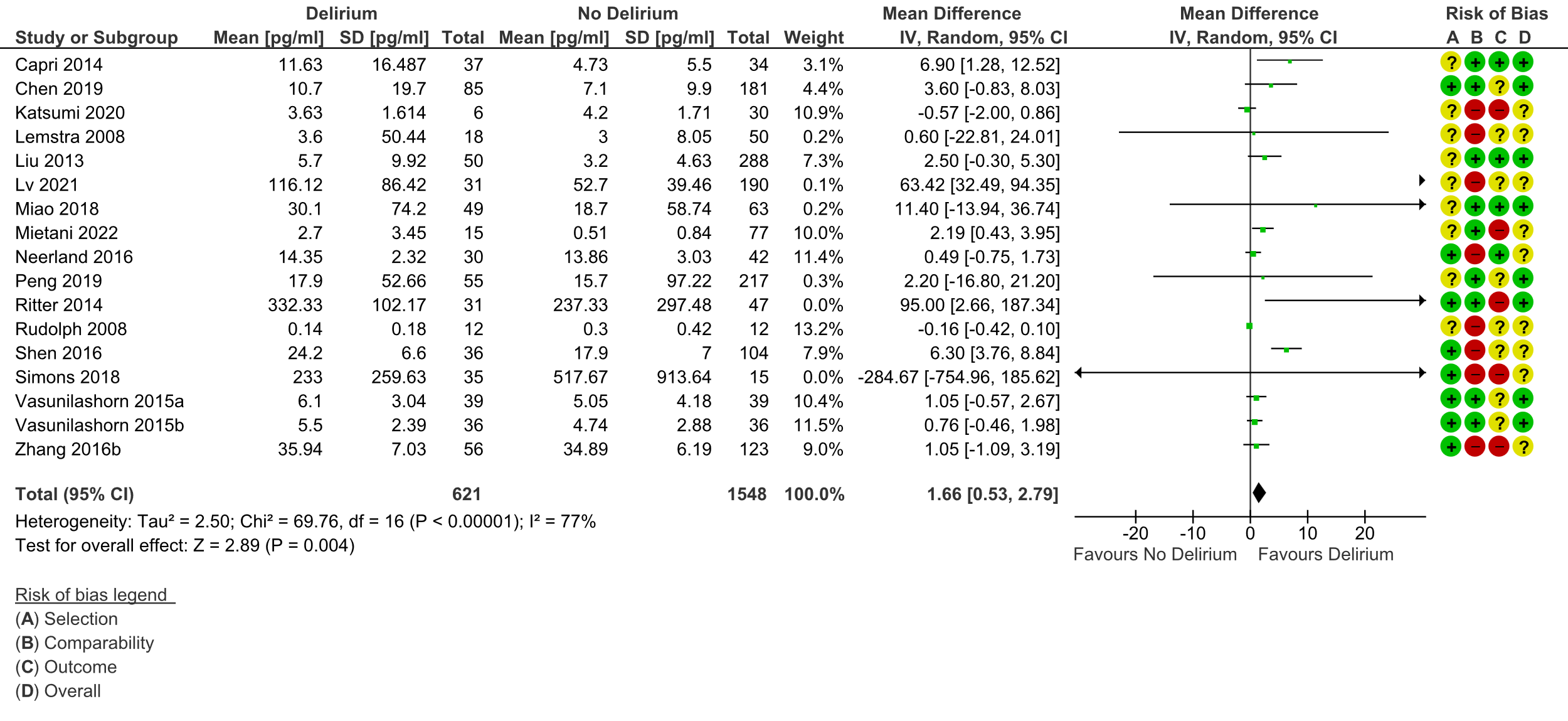


Low risk

Medium risk

High risk

Figure 4) Forest plots of meta-analysis of records measuring Platelet to Lymphocyte Ratio (PLR) preceding delirium, using random effects model. Mean difference (MD) and 95% CI (confidence interval) in participants that did and did not develop delirium. The green squares represent the mean difference for each study and the size of the square represents the weight of the study. The black lines represent the 95% CI. The black diamonds represent the overall MD.

## Supplementary Forest Plots of Inflammatory Markers Measured During Delirium


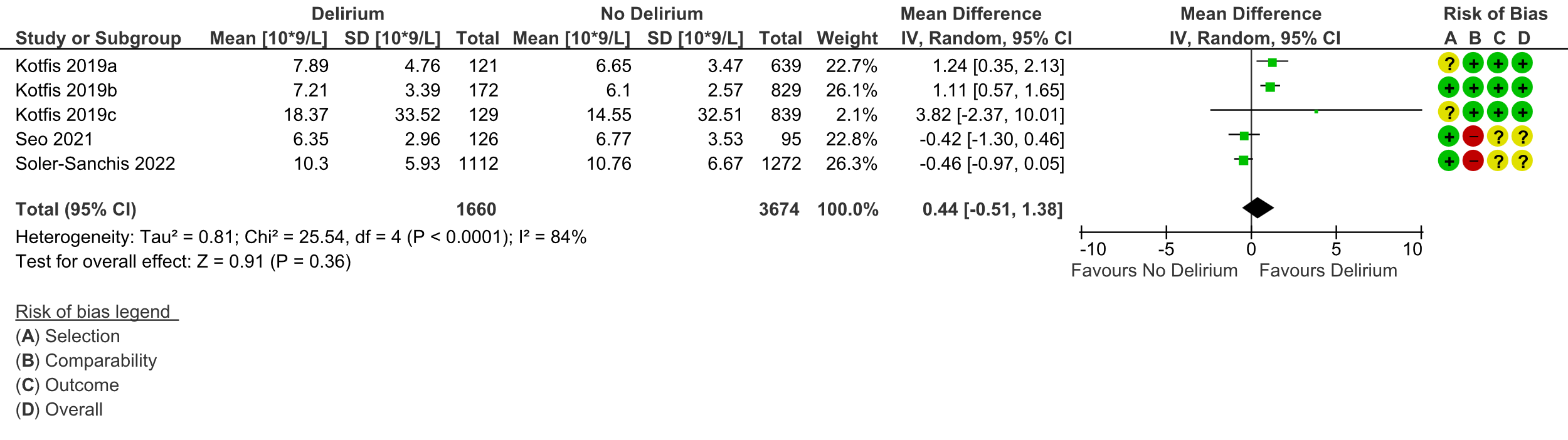

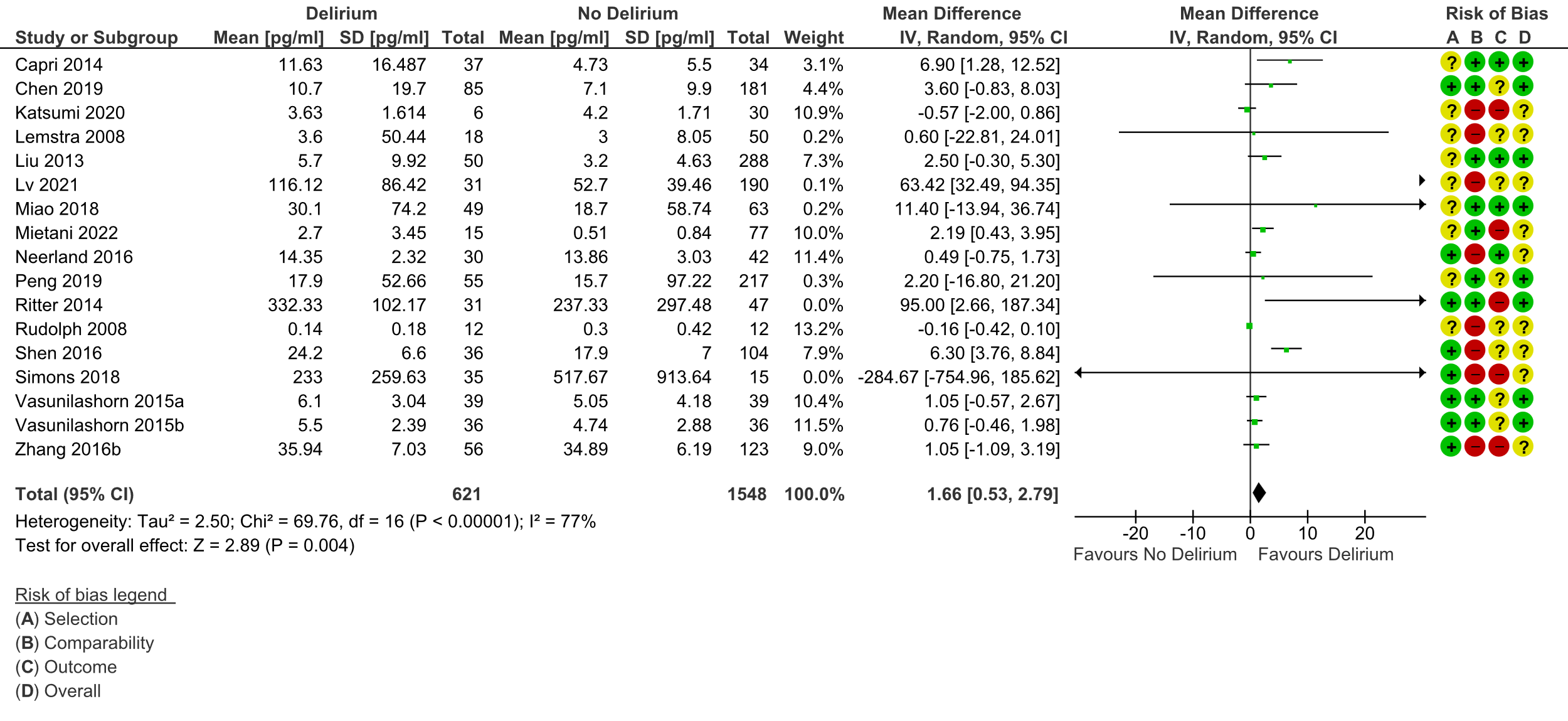


Low risk

Medium risk

High risk


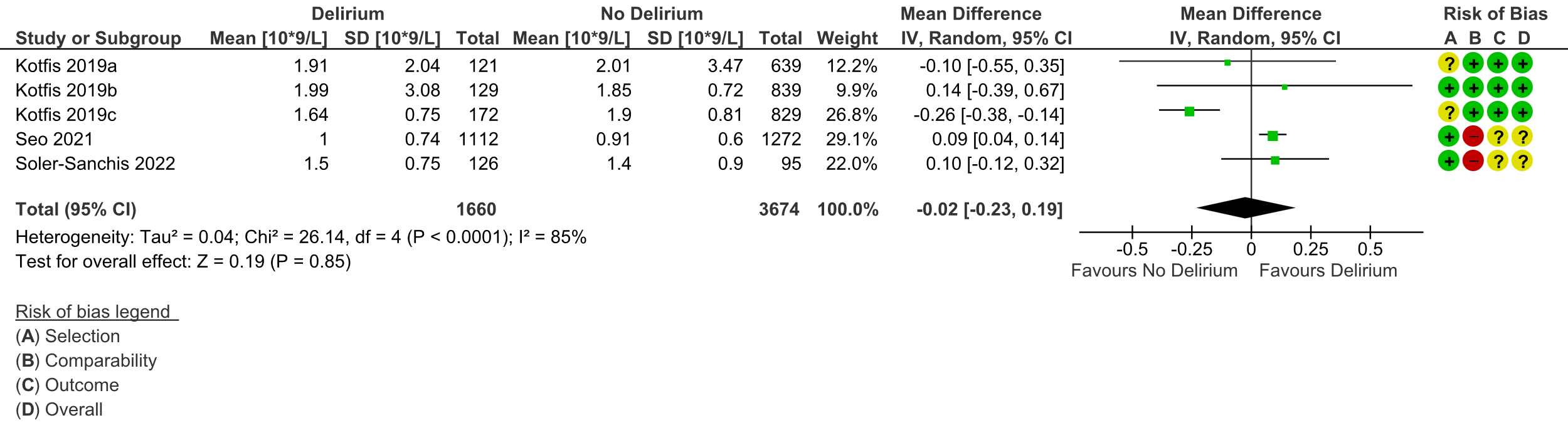

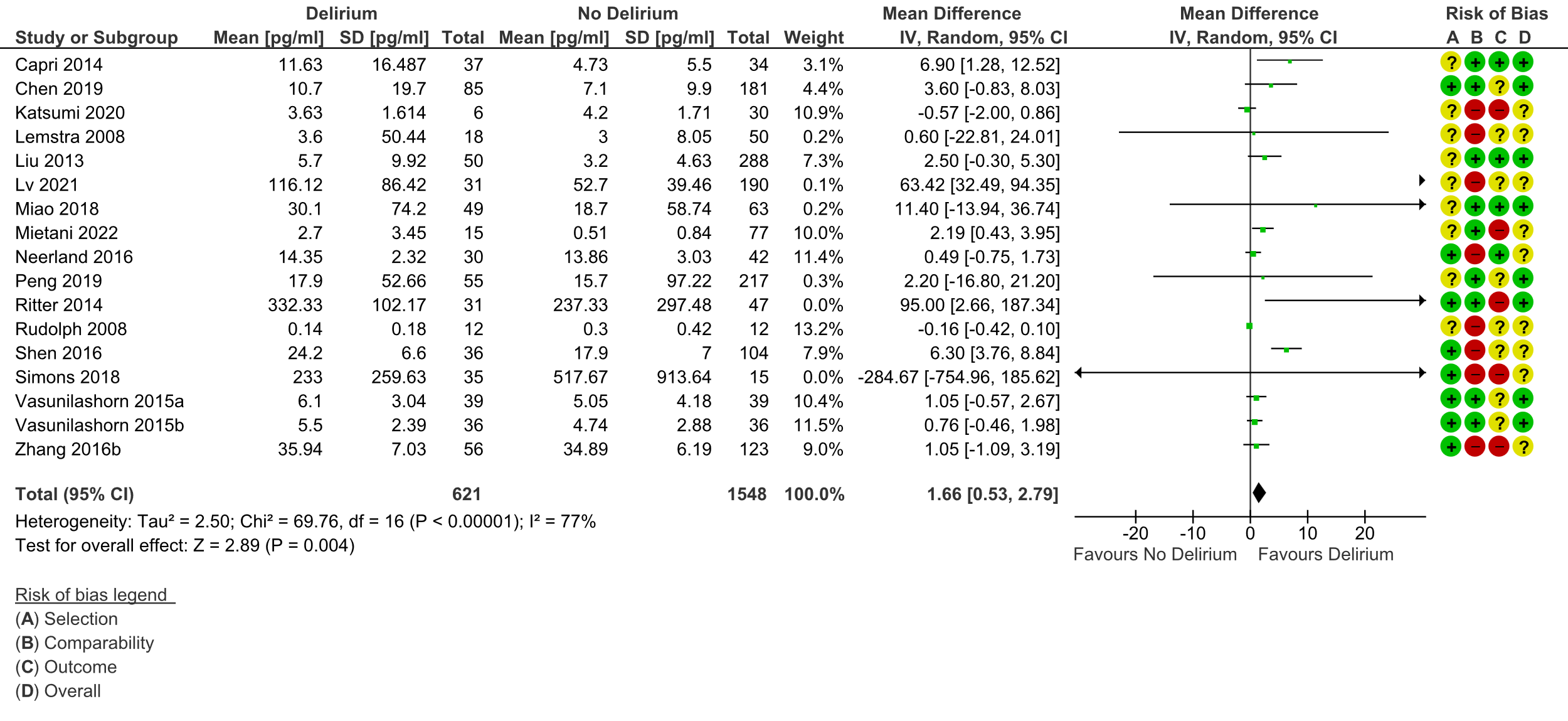


Low risk

Medium risk

High risk

Figure 6) Forest plots of meta-analysis of records measuring Lymphocyte Count (10^9^/L) during delirium, using random effects model. Mean difference (MD) and 95% CI (confidence interval) in participants with and without delirium. The green squares represent the mean difference for each study and the size of the square represents the weight of the study. The black lines represent the 95% CI. The black diamonds represent the overall MD.

Figure 5) Forest plots of meta-analysis of records measuring Neutrophil Count (10^9^/L) during delirium, using random effects model. Mean difference (MD) and 95% CI (confidence interval) in participants with and without delirium. The green squares represent the mean difference for each study and the size of the square represents the weight of the study. The black lines represent the 95% CI. The black diamonds represent the overall MD.

## Supplementary Forest Plots of Subgroup Analysis with Inflammatory Markers Measured Preceding Delirium


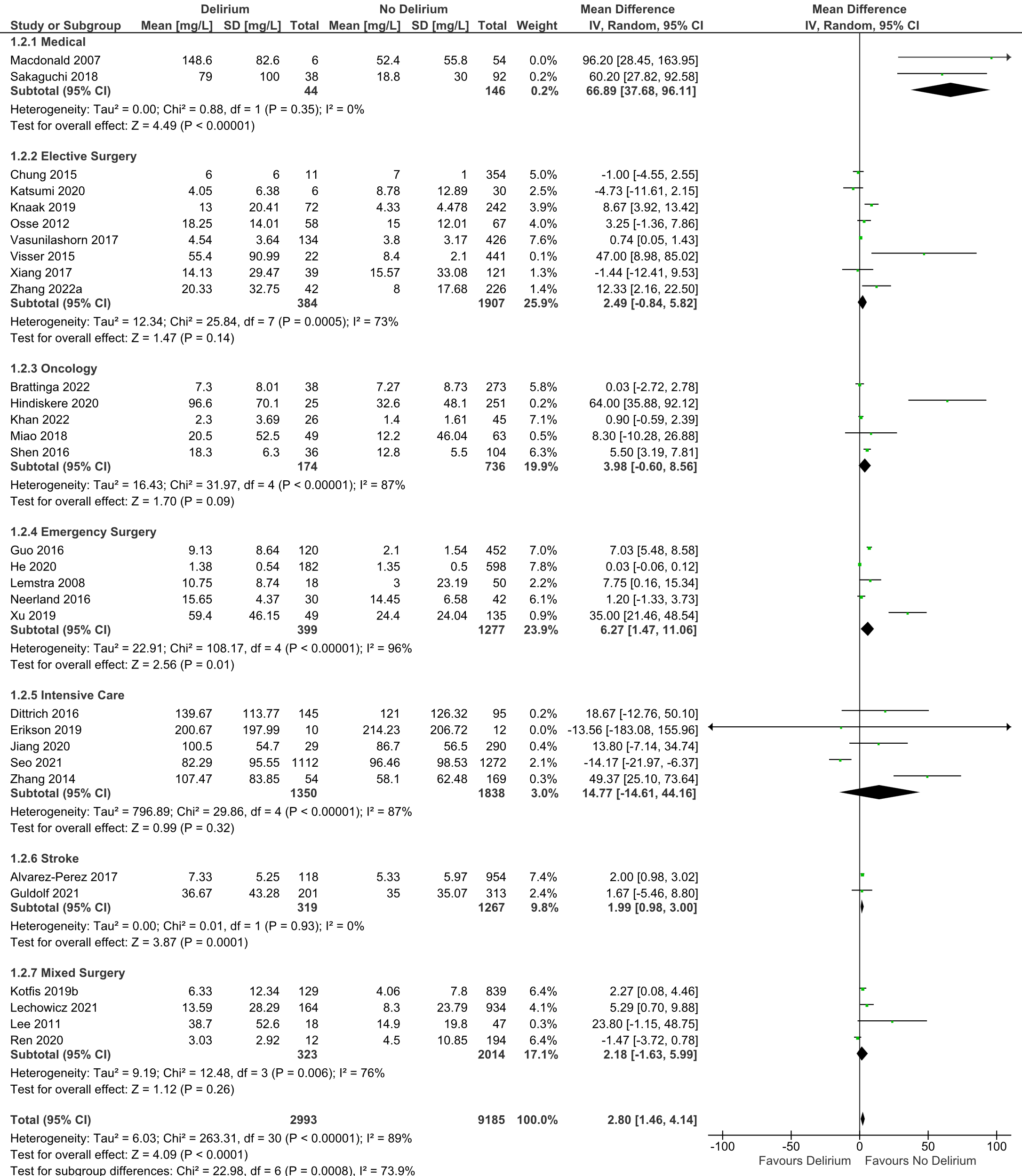


Figure 7) Forest plots of meta-analysis of records measuring C-Reactive Protein (CRP) (mg/L) preceding delirium, using random effects model. Mean difference (MD) and 95% CI (confidence interval) in participants that did and did not develop delirium. The green squares represent the mean difference for each study and the size of the square represents the weight of the study. The black lines represent the 95% CI. The black diamonds represent the overall MD. Records split into groups of different clinical populations.


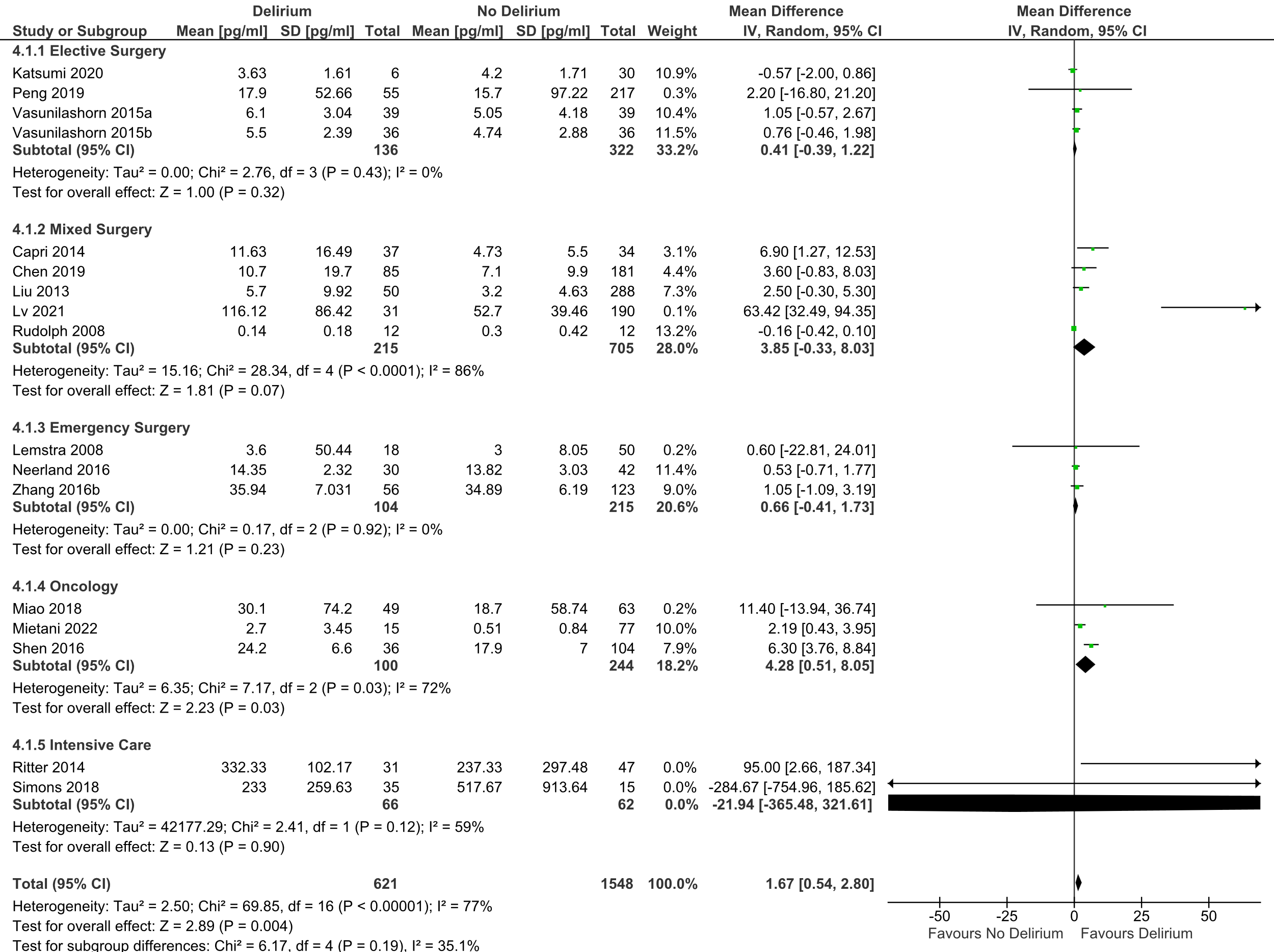


Figure 8) Forest plots of meta-analysis of records measuring Interleukin-6 (IL-6) (pg/ml) preceding delirium, using random effects model. Mean difference (MD) and 95% CI (confidence interval) in participants that did and did not develop delirium. The green squares represent the mean difference for each study and the size of the square represents the weight of the study. The black lines represent the 95% CI. The black diamonds represent the overall MD. Records split into groups of different clinical populations.

###
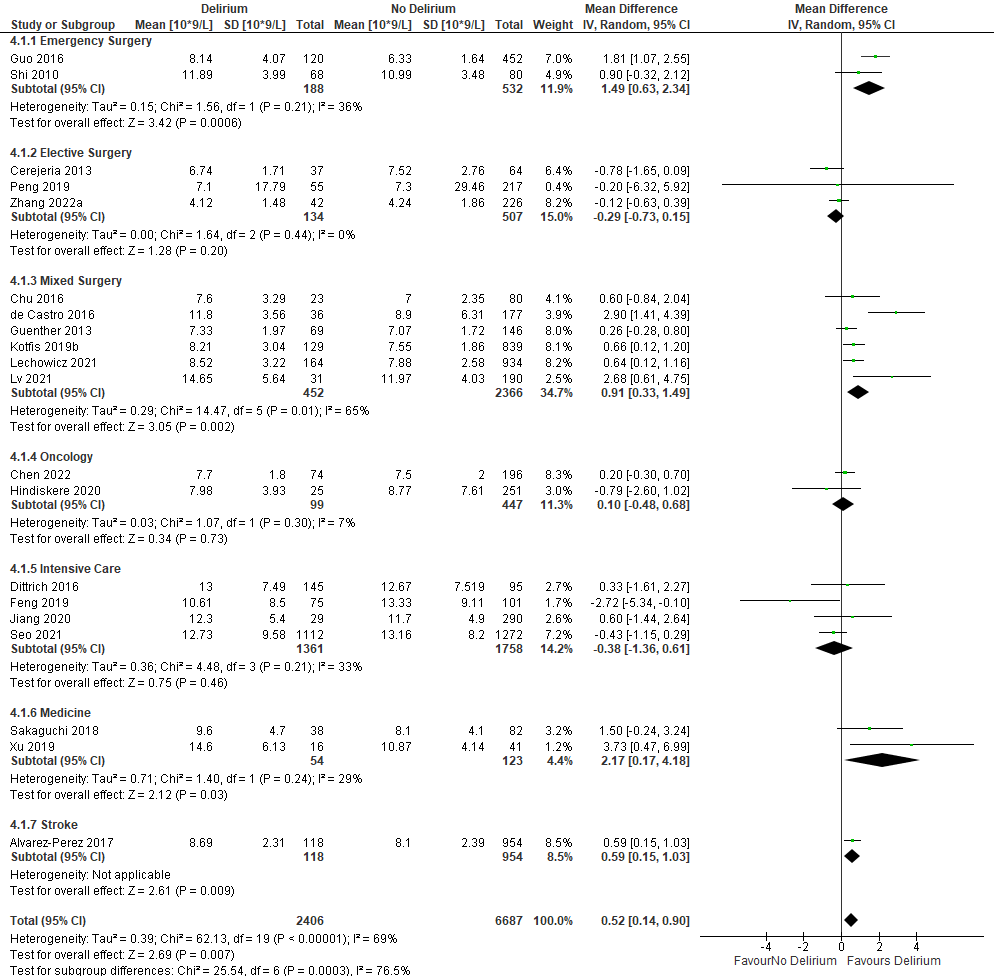


Figure 9) Forest plots of meta-analysis of records measuring Leucocyte Count (10^9^/L) preceding delirium, using random effects model. Mean difference (MD) and 95% CI (confidence interval) in participants that did and did not develop delirium. The green squares represent the mean difference for each study and the size of the square represents the weight of the study. The black lines represent the 95% CI. The black diamonds represent the overall MD and MD in each subgroup. Records split into groups of different clinical populations.

###
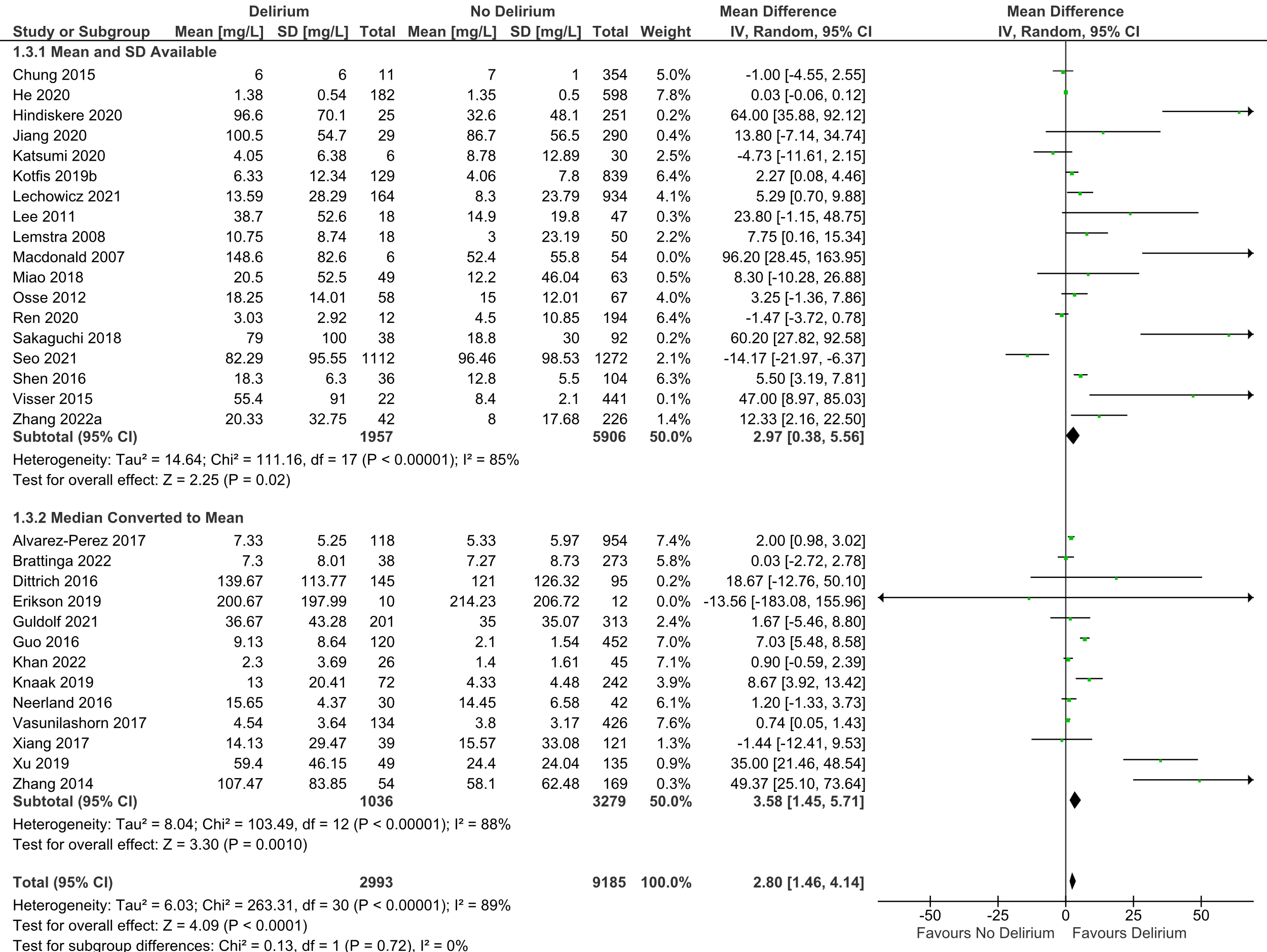


Figure 10) Forest plots of meta-analysis of records measuring C-Reactive Protein (CRP) (mg/L) preceding delirium, using random effects model. Mean difference (MD) and 95% CI (confidence interval) in participants that did and did not develop delirium. The green squares represent the mean difference for each study and the size of the square represents the weight of the study. The black lines represent the 95% CI. The black diamonds represent the overall MD. Records split into two groups of those that reported a mean and standard deviation (SD) and those that reported a median which was converted to a mean.


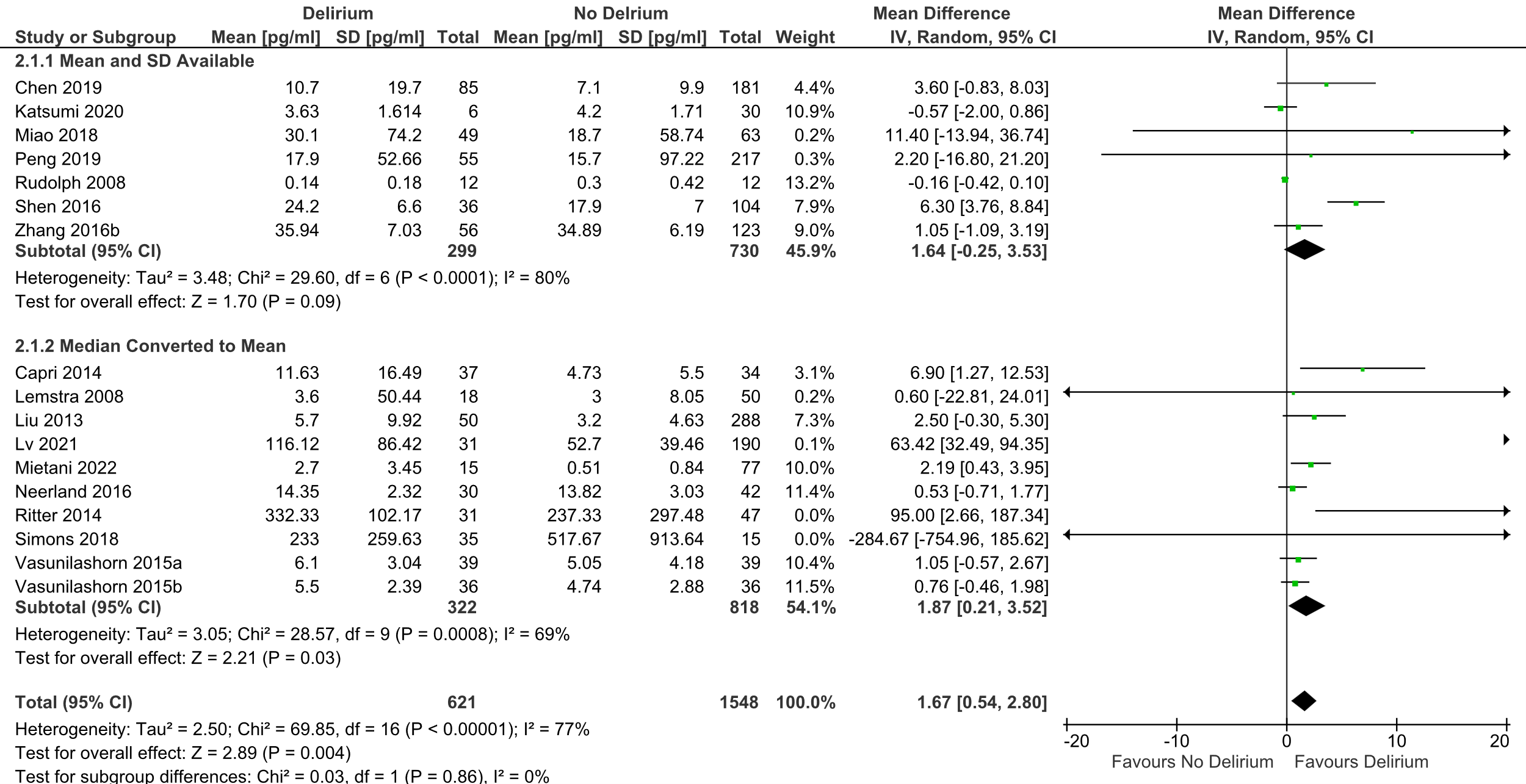


Figure 11) Forest plots of meta-analysis of records measuring Interleukin-6 (IL-6) (pg/ml) preceding delirium, using random effects model. Mean difference (MD) and 95% CI (confidence interval) in participants that did and did not develop delirium. The green squares represent the mean difference for each study and the size of the square represents the weight of the study. The black lines represent the 95% CI. The black diamonds represent the overall MD. Records split into two groups of those that reported a mean and standard deviation (SD) and those that reported a median which was converted to a mean.

###
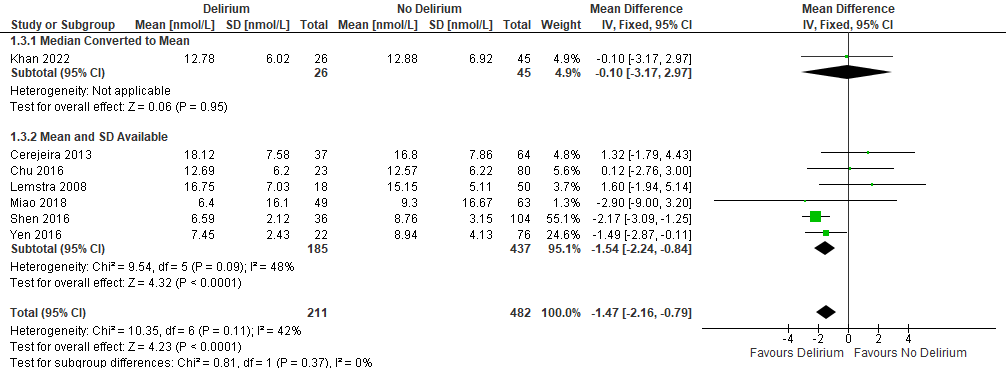

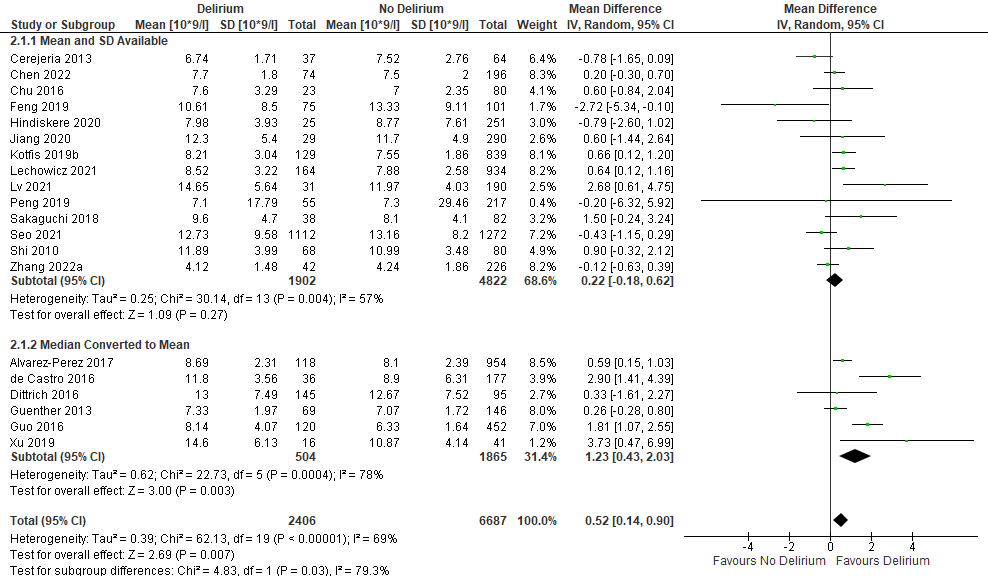


Figure 12) Forest plots of meta-analysis of records measuring Insulin-Like Growth Factor-1 (IGF-1) (nmol/L) preceding delirium, using fixed effects model. Mean difference (MD) and 95% CI (confidence interval) in participants that did and did not develop delirium. The green squares represent the mean difference for each study and the size of the square represents the weight of the study. The black lines represent the 95% CI. The black diamonds represent the overall MD and MD in each subgroup. Records split into two groups of those that reported a mean and standard deviation (SD) and those that reported a median which was converted to a mean.

Figure 13) Forest plots of meta-analysis of records measuring Leucocyte Count (10^9^/L) preceding delirium, using random effects model. Mean difference (MD) and 95% CI (confidence interval) in participants that did and did not develop delirium. The green squares represent the mean difference for each study and the size of the square represents the weight of the study. The black lines represent the 95% CI. The black diamonds represent the overall MD and MD in each subgroup. Records split into two groups of those that reported a mean and standard deviation (SD) and those that reported a median which was converted to a mean.

###
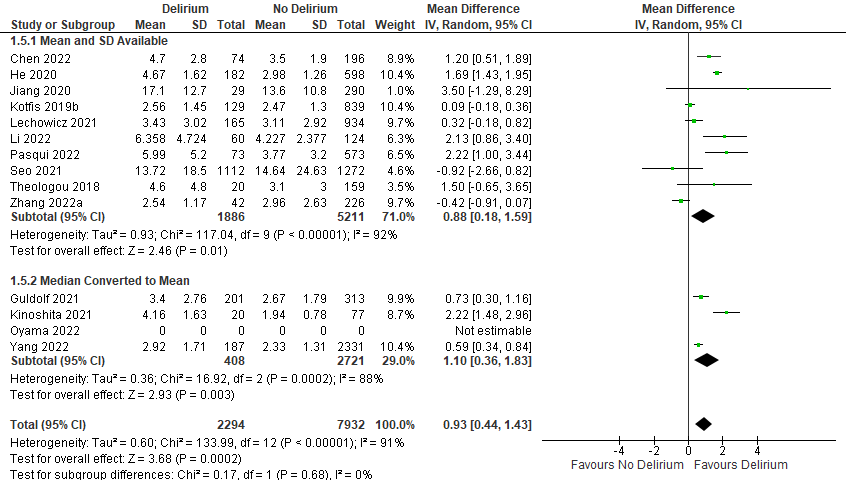


Figure 14) Forest plots of meta-analysis of records measuring Neutrophil to Lymphocyte Ratio (NLR) preceding delirium, using random effects model. Mean difference (MD) and 95% CI (confidence interval) in participants that did and did not develop delirium. The green squares represent the mean difference for each study and the size of the square represents the weight of the study. The black lines represent the 95% CI. The black diamonds represent the overall MD and MD in each subgroup. Records split into two groups of those that reported a mean and standard deviation (SD) and those that reported a median which was converted to a mean.

###
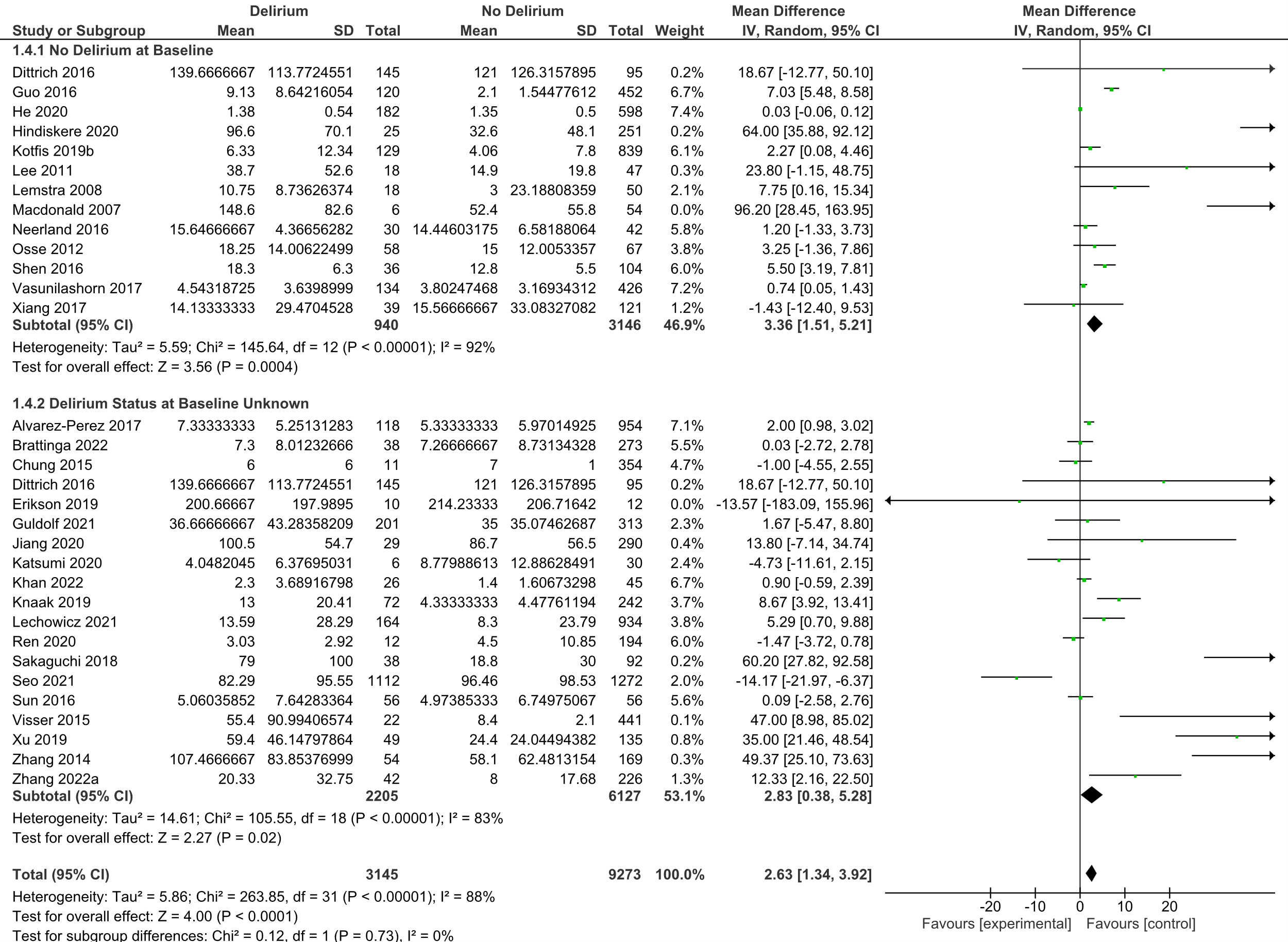


Figure 15) Forest plots of meta-analysis of records measuring C-Reactive Protein (CRP) (mg/L) preceding delirium, using random effects model. Mean difference (MD) and 95% CI (confidence interval) in participants that did and did not develop delirium. The green squares represent the mean difference for each study and the size of the square represents the weight of the study. The black lines represent the 95% CI. The black diamonds represent the overall MD and MD in each subgroup. Records split into two groups of those that there was no delirium at baseline and those that did not screen for delirium at baseline or did not report this.

###
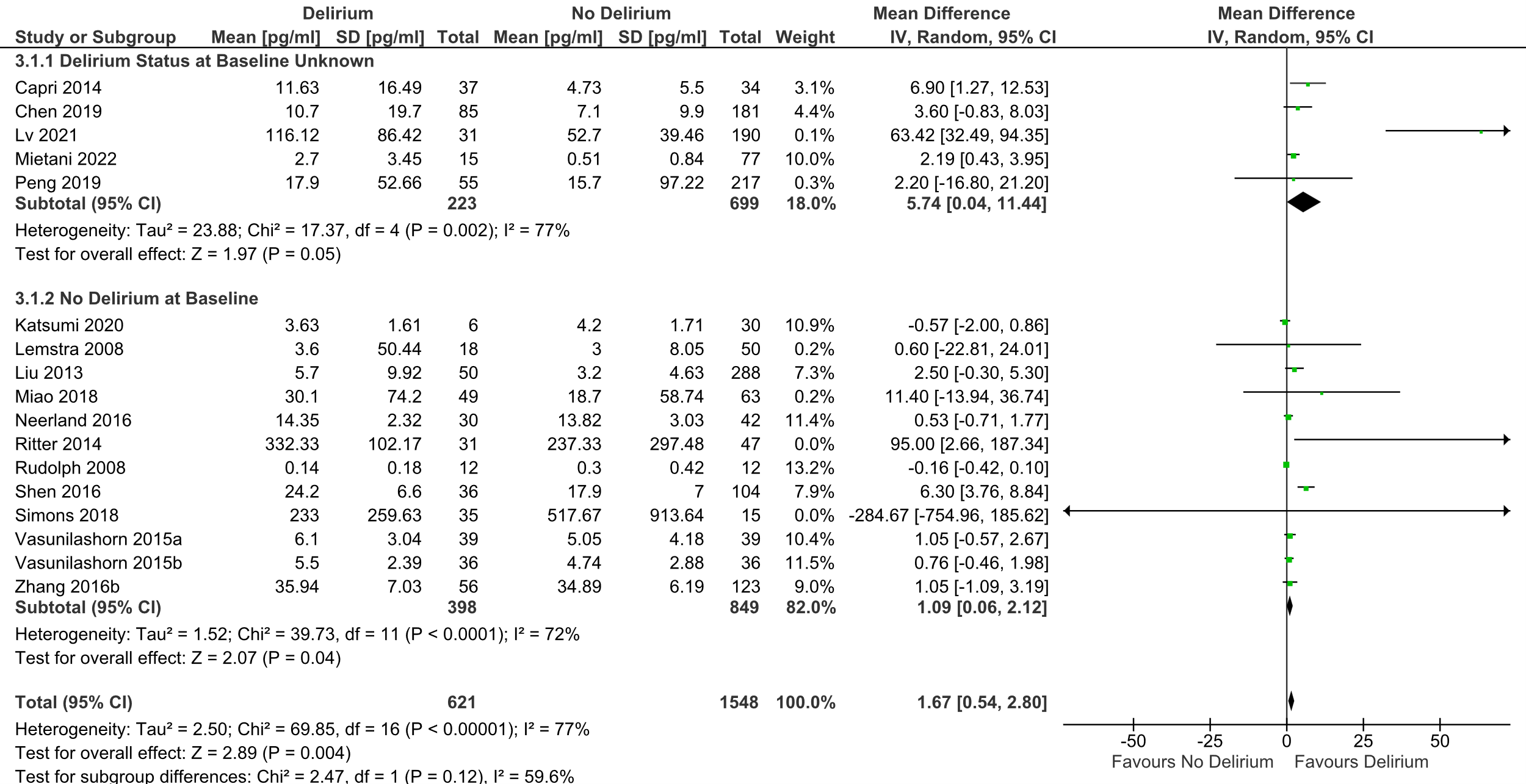


Figure 16) Forest plots of meta-analysis of records measuring Interleukin-6 (IL-6) (pg/ml) preceding delirium, using random effects model. Mean difference (MD) and 95% CI (confidence interval) in participants that did and did not develop delirium. The green squares represent the mean difference for each study and the size of the square represents the weight of the study. The black lines represent the 95% CI. The black diamonds represent the overall MD and MD in each subgroup. Records split into two groups of those that there was no delirium at baseline and those that did not screen for delirium at baseline or did not report this.

###
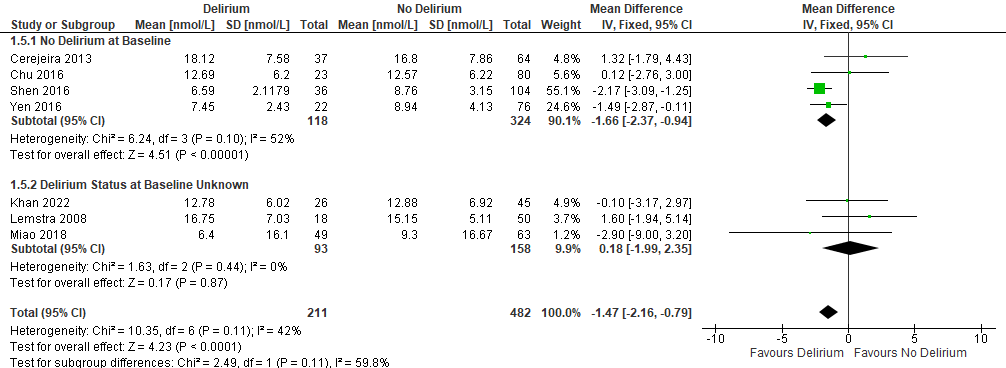


Figure 17) Forest plots of meta-analysis of records measuring Insulin-Like Growth Factor-1 (IGF-1) (nmol/L) preceding delirium, using fixed effects model. Mean difference (MD) and 95% CI (confidence interval) in participants that did and did not develop delirium. The green squares represent the mean difference for each study and the size of the square represents the weight of the study. The black lines represent the 95% CI. The black diamonds represent the overall MD and MD in each subgroup. Records split into two groups of those that there was no delirium at baseline and those that did not screen for delirium at baseline or did not report this.

###
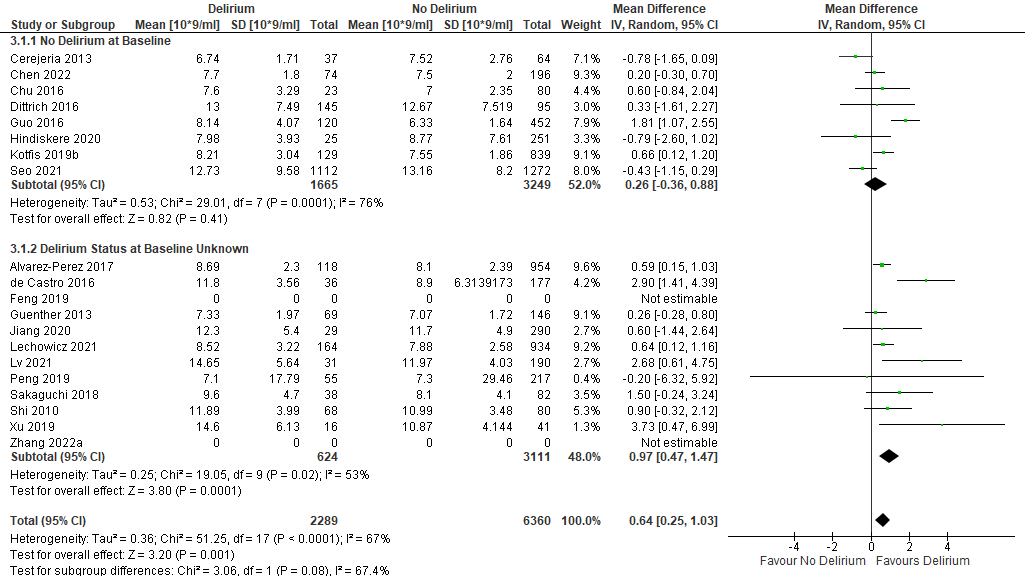


Figure 18) Forest plots of meta-analysis of records measuring Leucocyte Count (10^9^/L) preceding delirium, using random effects model. Mean difference (MD) and 95% CI (confidence interval) in participants that did and did not develop delirium. The green squares represent the mean difference for each study and the size of the square represents the weight of the study. The black lines represent the 95% CI. The black diamonds represent the overall MD and MD in each subgroup. Records split into two groups of those that there was no delirium at baseline and those that did not screen for delirium at baseline or did not report this.

###
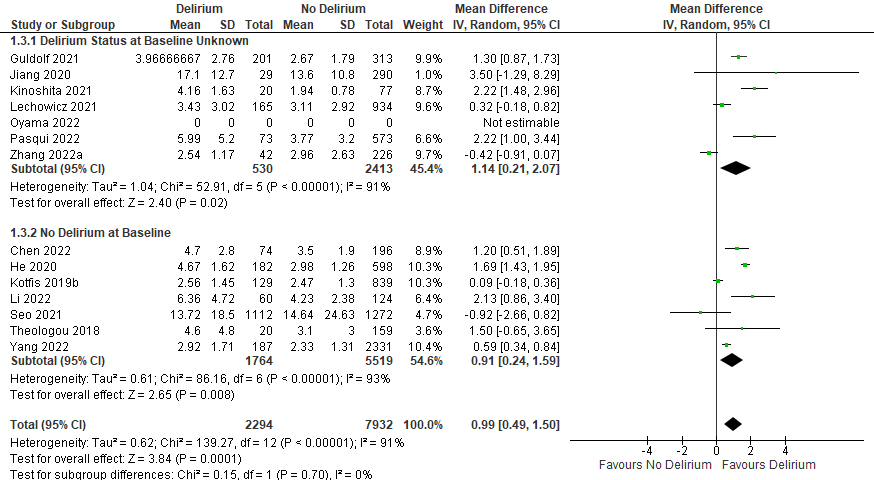


Figure 19) Forest plots of meta-analysis of records measuring Neutrophil to Lymphocyte Ratio (NLR) preceding delirium, using random effects model. Mean difference (MD) and 95% CI (confidence interval) in participants that did and did not develop delirium. The green squares represent the mean difference for each study and the size of the square represents the weight of the study. The black lines represent the 95% CI. The black diamonds represent the overall MD and MD in each subgroup. Records split into two groups of those that there was no delirium at baseline and those that did not screen for delirium at baseline or did not report this.

## Forest Plots of Subgroup Analysis with Inflammatory Markers Measured During Delirium


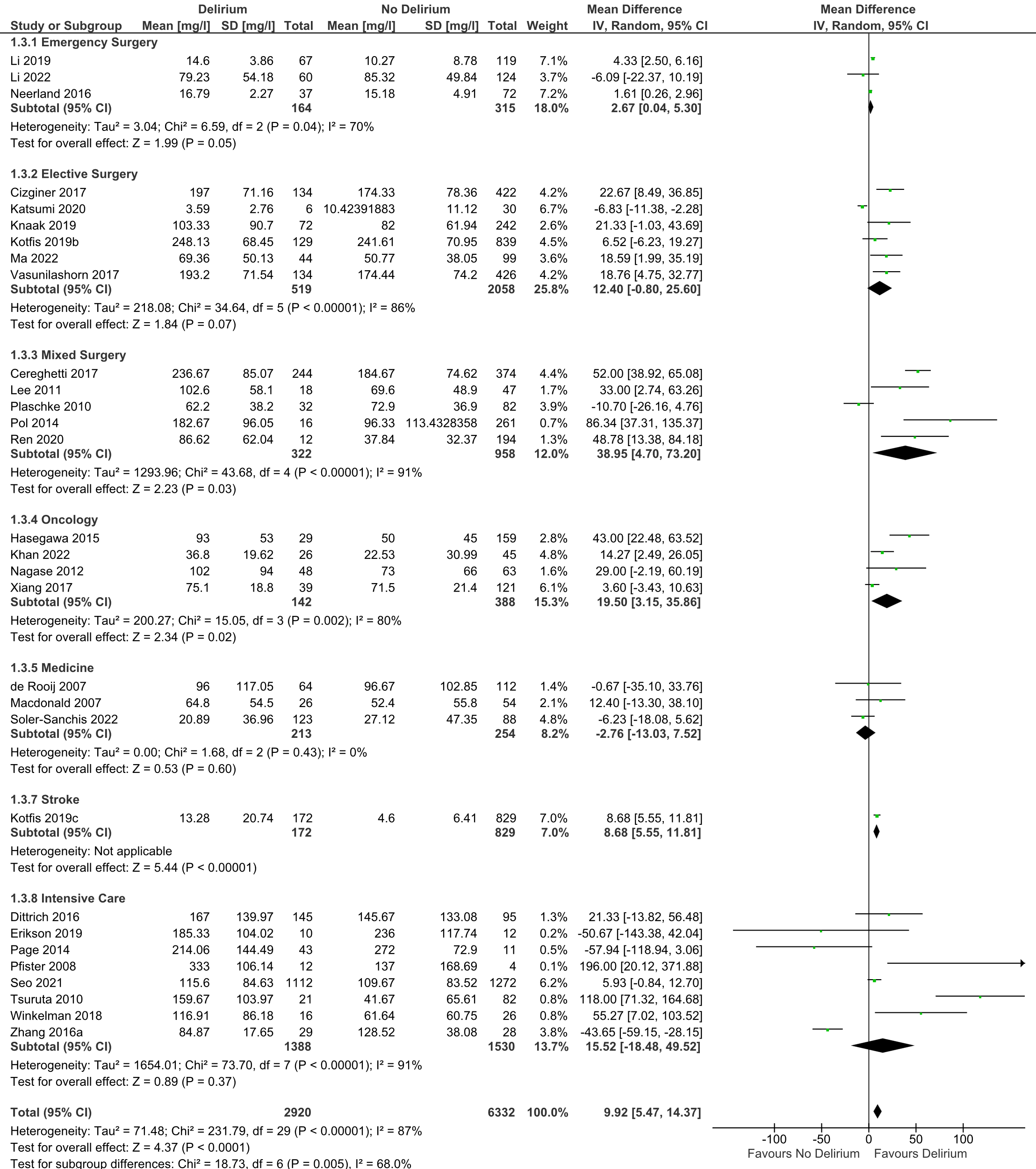


Figure 20) Forest plots of meta-analysis of records measuring C-Reactive Protein (CRP) (mg/L) during delirium, using random effects model. Mean difference (MD) and 95% CI (confidence interval) in participants with and without delirium. The green squares represent the mean difference for each study and the size of the square represents the weight of the study. The black lines represent the 95% CI. The black diamonds represent the overall MD. Records split into groups of different clinical populations.


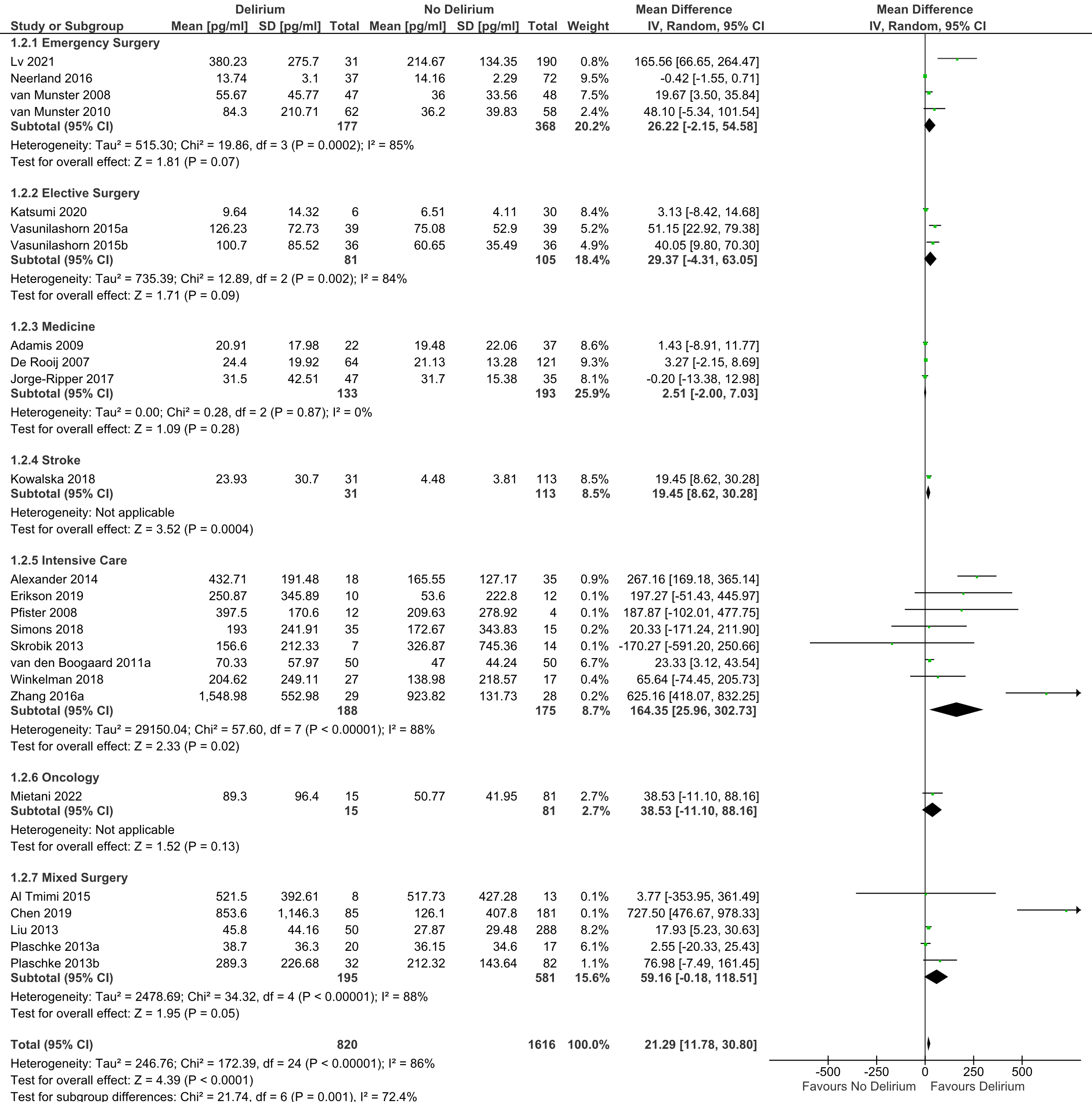


Figure 21) Forest plots of meta-analysis of records measuring Interleukin-6 (IL-6) (pg/ml) during delirium, using random effects model. Mean difference (MD) and 95% CI (confidence interval) in participants with and without delirium. The green squares represent the mean difference for each study and the size of the square represents the weight of the study. The black lines represent the 95% CI. The black diamonds represent the overall MD. Records split into groups of different clinical populations.


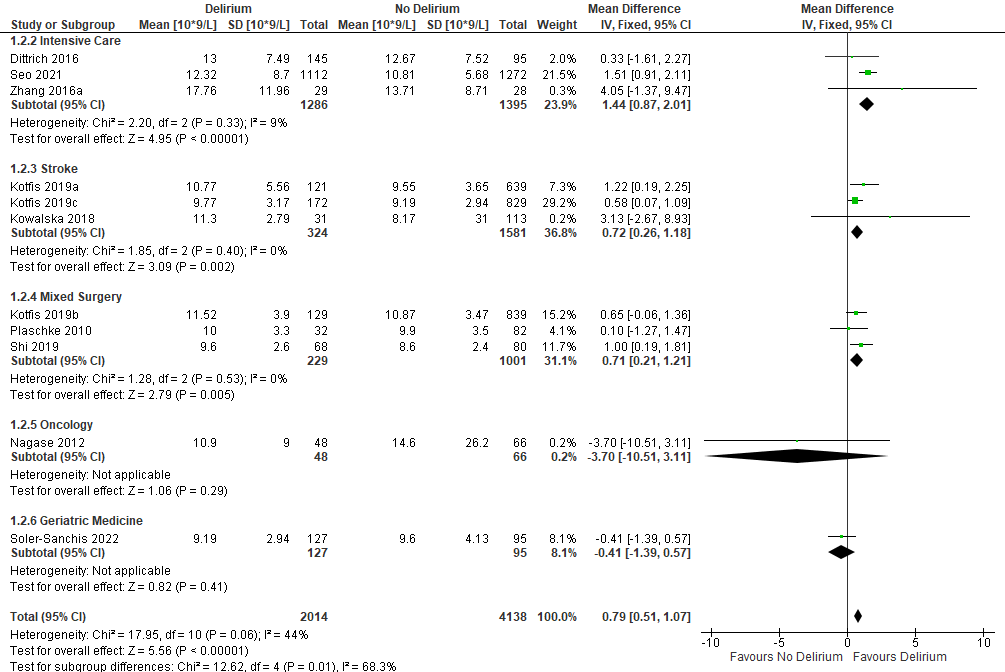


Figure 22) Forest plots of meta-analysis of records measuring Leucocyte Count (10^9^/L) during delirium, using fixed effects model. Mean difference (MD) and 95% CI (confidence interval) in participants with and without delirium. The green squares represent the mean difference for each study and the size of the square represents the weight of the study. The black lines represent the 95% CI. The black diamonds represent the overall MD. Records split into groups of different clinical populations.


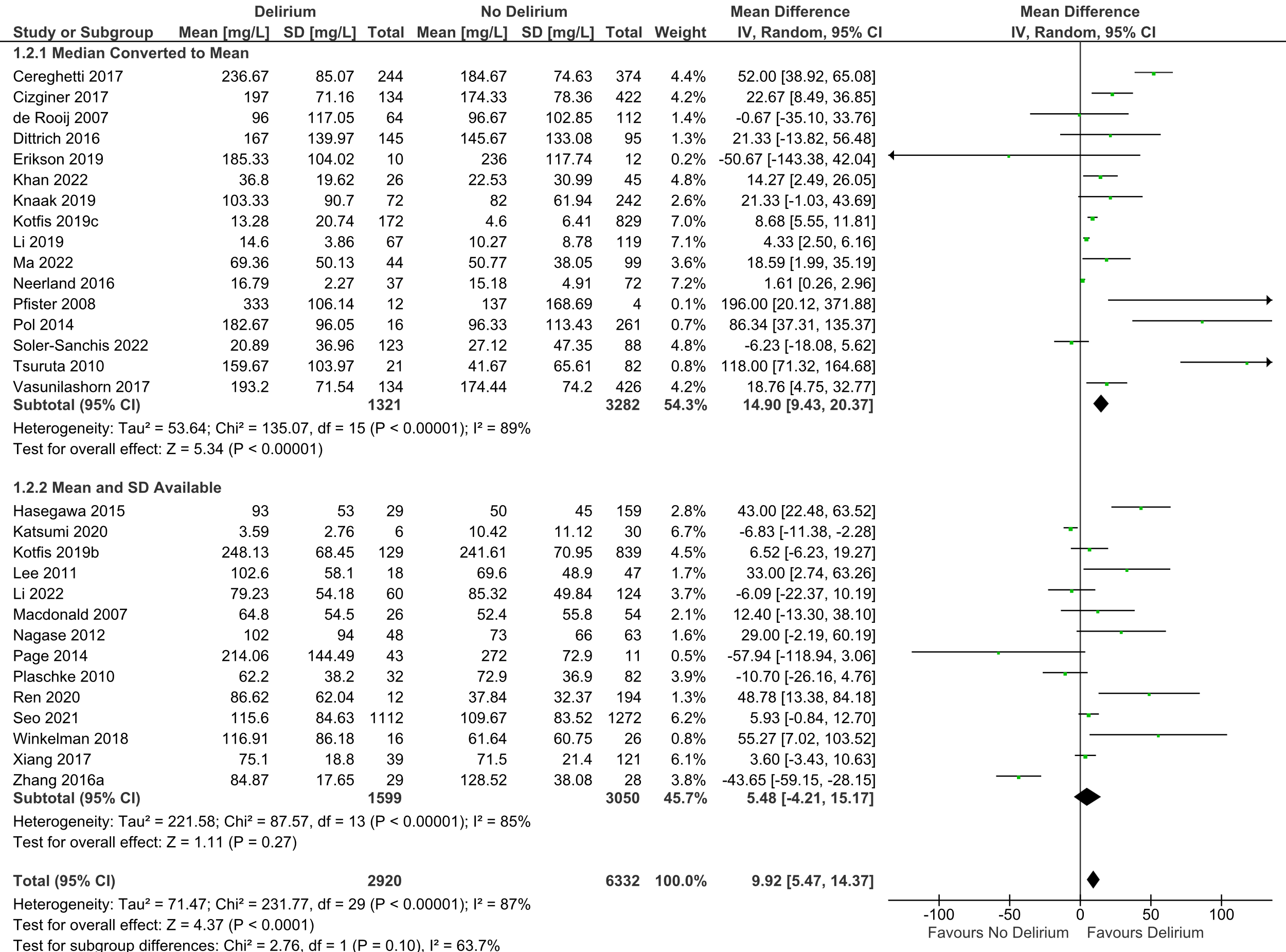


Figure 23) Forest plots of meta-analysis of records measuring C-Reactive Protein (CRP) (mg/L) during delirium, using random effects model. Mean difference (MD) and 95% CI (confidence interval) in participants with and without delirium. The green squares represent the mean difference for each study and the size of the square represents the weight of the study. The black lines represent the 95% CI. The black diamonds represent the overall MD. Records split into two groups of those that reported a mean and standard deviation (SD) and those that reported a median which was converted to a mean.


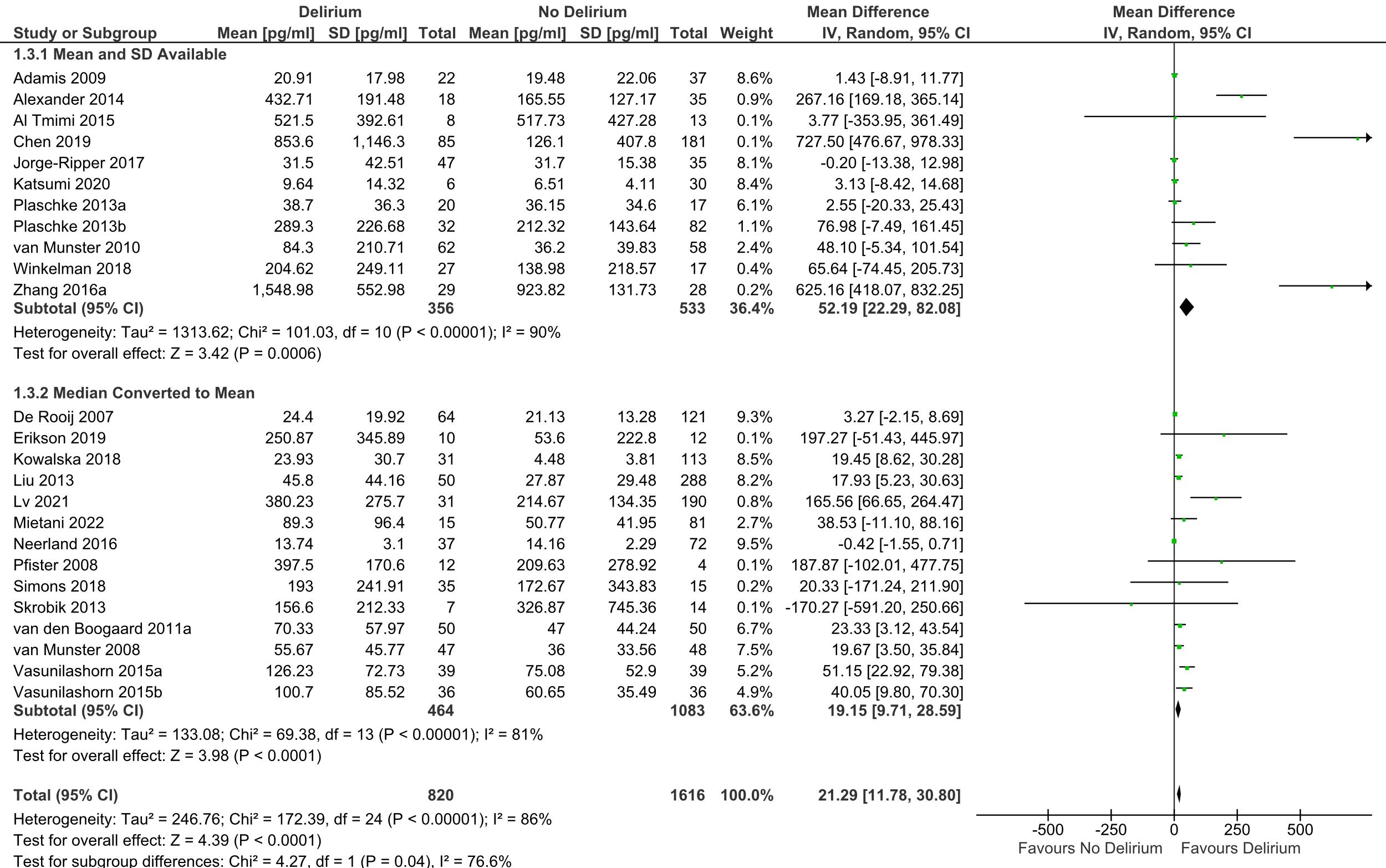


Figure 24) Forest plots of meta-analysis of records measuring Interleukin-6 (IL-6) (pg/ml) during delirium, using random effects model. Mean difference (MD) and 95% CI (confidence interval) in participants with and without delirium. The green squares represent the mean difference for each study and the size of the square represents the weight of the study. The black lines represent the 95% CI. The black diamonds represent the overall MD. Records split into two groups of those that reported a mean and standard deviation (SD) and those that reported a median which was converted to a mean.


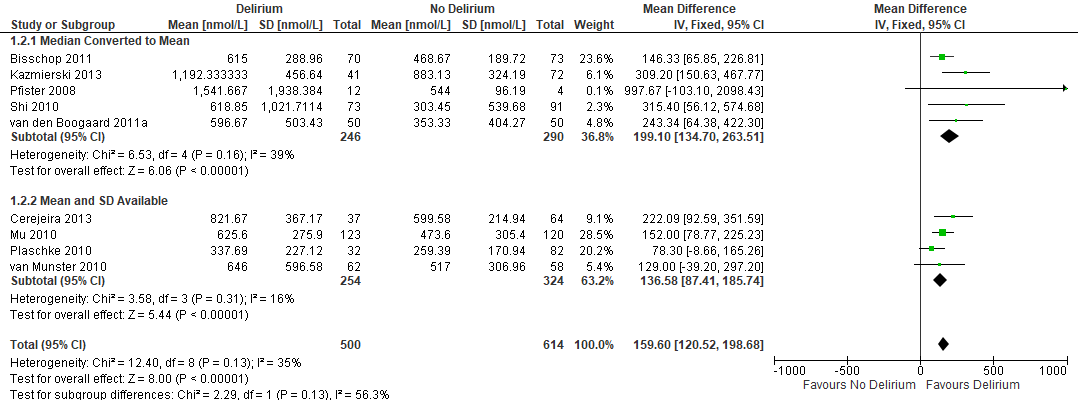


Figure 25) Forest plots of meta-analysis of records measuring Cortisol (nmol/L) during delirium, using random effects model. Mean difference (MD) and 95% CI (confidence interval) in participants with and without delirium. The green squares represent the mean difference for each study and the size of the square represents the weight of the study. The black lines represent the 95% CI. The black diamonds represent the overall MD. Records split into two groups of those that reported a mean and standard deviation (SD) and those that reported a median which was converted to a mean.


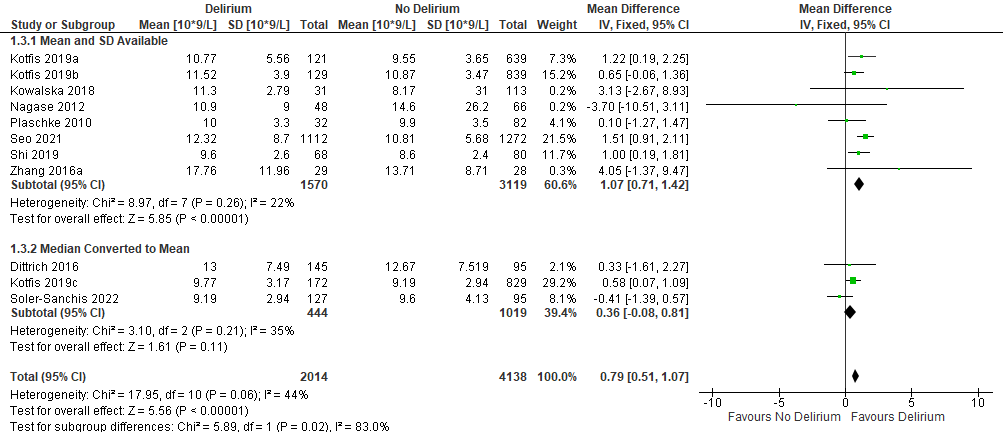


Figure 26) Forest plots of meta-analysis of records measuring Leucocyte Count (10^9^/L) during delirium, using fixed effects model. Mean difference (MD) and 95% CI (confidence interval) in participants with and without delirium. The green squares represent the mean difference for each study and the size of the square represents the weight of the study. The black lines represent the 95% CI. The black diamonds represent the overall MD. Records split into two groups of those that reported a mean and standard deviation (SD) and those that reported a median which was converted to a mean.

###
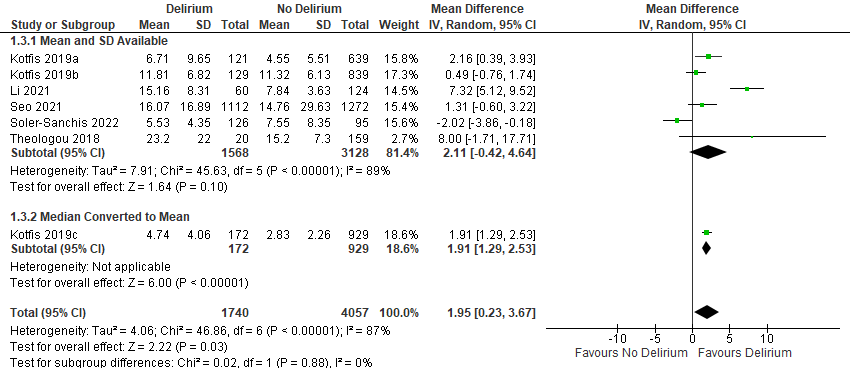


Figure 27) Forest plots of meta-analysis of records measuring Neutrophil to Lymphocyte Ratio (NLR) during delirium, using random effects model. Mean difference (MD) and 95% CI (confidence interval) in participants with and without delirium. The green squares represent the mean difference for each study and the size of the square represents the weight of the study. The black lines represent the 95% CI. The black diamonds represent the overall MD. Records split into two groups of those that reported a mean and standard deviation (SD) and those that reported a median which was converted to a mean.

###
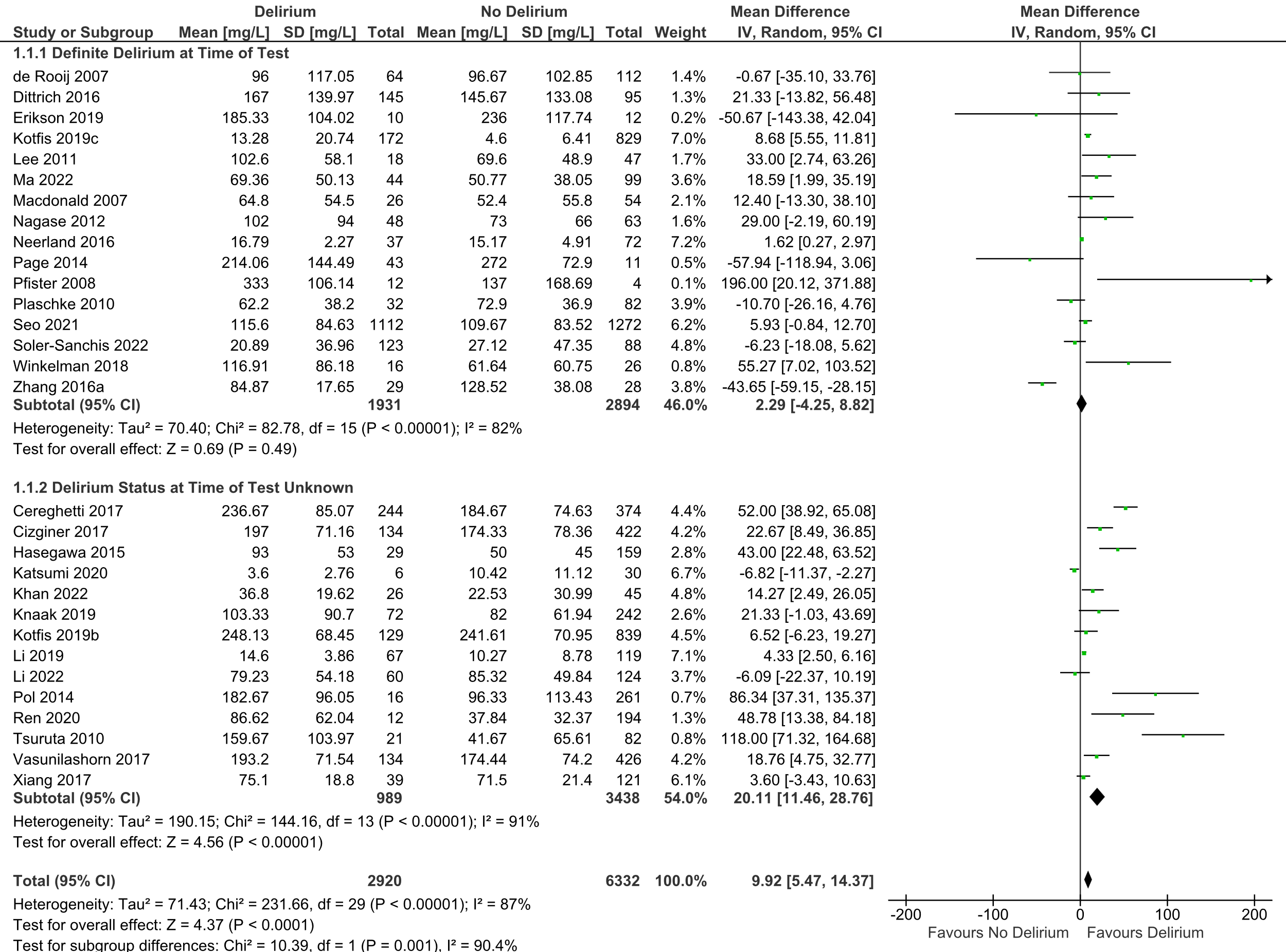


Figure 28) Forest plots of meta-analysis of records measuring C-reactive Protein (CRP) (mg/L) during delirium, using random effects model. Mean difference (MD) and 95% CI (confidence interval) in participants with and without delirium. The green squares represent the mean difference for each study and the size of the square represents the weight of the study. The black lines represent the 95% CI. The black diamonds represent the overall MD. Records split into two groups of those that definitely had delirium at time of test and those that did not did not report this.


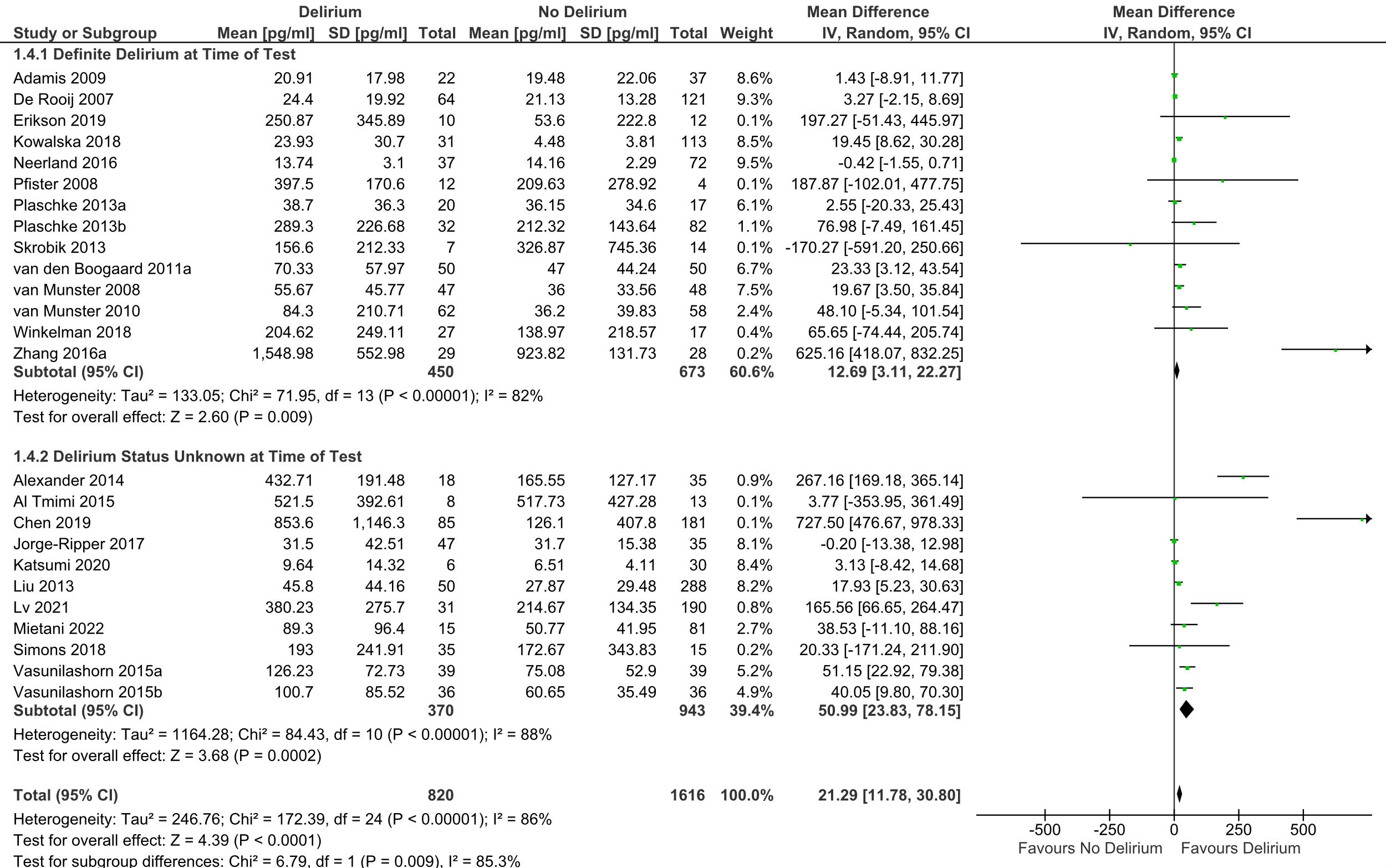


Figure 29) Forest plots of meta-analysis of records measuring Interleukin- 6 (IL-6) (pg/ml) during delirium, using random effects model. Mean difference (MD) and 95% CI (confidence interval) in participants with and without delirium. The green squares represent the mean difference for each study and the size of the square represents the weight of the study. The black lines represent the 95% CI. The black diamonds represent the overall MD. Records split into two groups of those that definitely had delirium at time of test and those that did not did not report this.

###
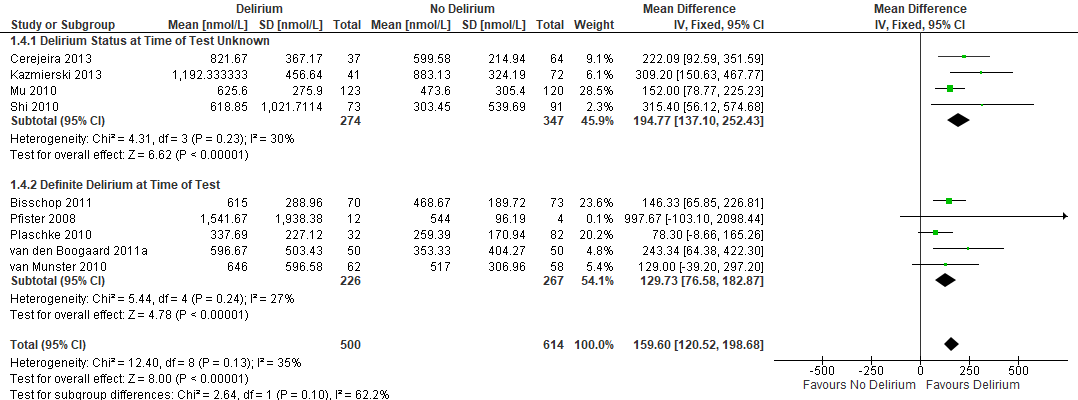


Figure 30) Forest plots of meta-analysis of records measuring Cortisol (nmol/L) during delirium, using fixed effects model. Mean difference (MD) and 95% CI (confidence interval) in participants with and without delirium. The green squares represent the mean difference for each study and the size of the square represents the weight of the study. The black lines represent the 95% CI. The black diamonds represent the overall MD. Records split into two groups of those that definitely had delirium at time of test and those that did not did not report this.


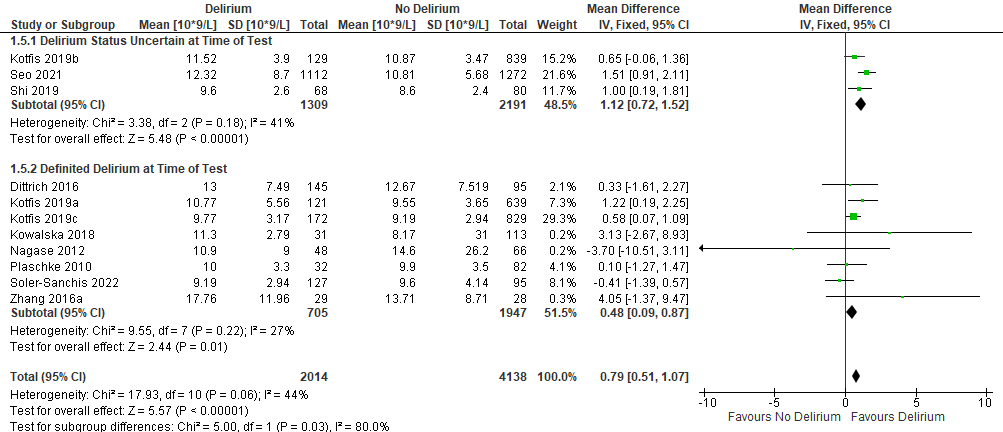


Figure 31) Forest plots of meta-analysis of records measuring Leucocyte Count (10^9^/L) during delirium, using fixed effects model. Mean difference (MD) and 95% CI (confidence interval) in participants with and without delirium. The green squares represent the mean difference for each study and the size of the square represents the weight of the study. The black lines represent the 95% CI. The black diamonds represent the overall MD. Records split into two groups of those that definitely had delirium at time of test and those that did not did not report this.


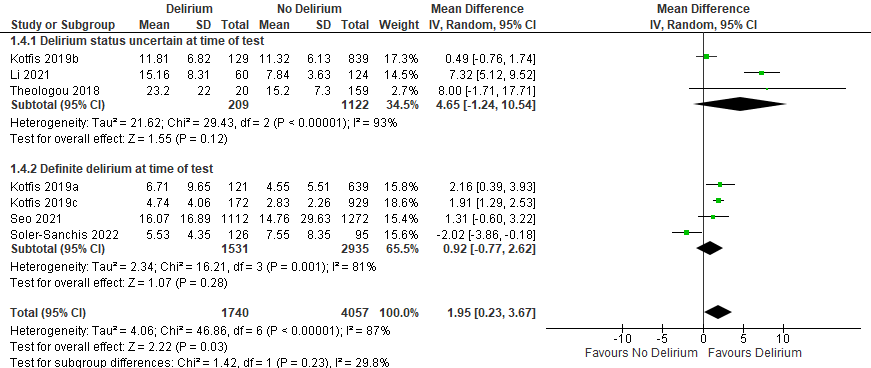


Figure 32) Forest plots of meta-analysis of records measuring Neutrophil to Lymphocyte Ratio (NLR) during delirium, using random effects model. Mean difference (MD) and 95% CI (confidence interval) in participants with and without delirium. The green squares represent the mean difference for each study and the size of the square represents the weight of the study. The black lines represent the 95% CI. The black diamonds represent the overall MD. Records split into two groups of those that definitely had delirium at time of test and those that did not did not report this.

## Risk Of Bias Tables

Table 11 Risk of Bias of Studies Included in the Systematic Review Measured with the Newcastle Ottawa Scale for Cohort Studies . High Risk 0-3, Moderate risk 4-6, Low risk 7-9

| **Author** | SELECTION | | | | | COMPARABILITY | OUTCOME | | | | NOS Score |
| --- | --- | --- | --- | --- | --- | --- | --- | --- | --- | --- | --- |
|  | 1 | 2 | 3 | 4 | Overall |  | 1 | 2 | 3 | Overall |  |
| Adamis, D., et al., 2020 | a | a | a | b | *** | ** | d | a | d | * | 6 |
| Adamis, D., et al., 2009 | a | a | a | b | *** | ** | d | a | d | * | 6 |
| Adamis, D., et al., 2007 | a | a | a | b | *** | ** | d | a | d | * | 6 |
| Al Tmimi, L., et al., 2015 | b | a | a | a | **** | None | d | a | a | ** | 6 |
| Alexander, S. A., et al., 2014 | b | a | a | b | *** | ** | d | a | d | * | 6 |
| Alvarez-Perez, F. J. and Paiva, F., 2017 | a | a | a | b | *** | ** | d | a | d | * | 6 |
| Avila-Funes, J. A., et al., 2015 | b | a | a | a | **** | ** | d | a | d | * | 7 |
| Ballweg, T., et al., 2021 | b | a | a | b | *** | None | d | a | b | ** | 5 |
| Baranyi, A., and Rothenhausler, H. B., 2014 | b | a | a | b | *** | None | d | a | d | * | 4 |
| Beloosesky, Y., et al., 2007 | a | a | a | b | *** | None | d | b | d | None | 3 |
| Beloosesky, Y., et al., 2004 | a | a | a | b | *** | None | d | b | d | None | 3 |
| Bisschop, P. H., et al., 2011 | a | a | a | b | *** | ** | d | a | d | * | 6 |
| Brattinga, B., et al., 2022 | a | a | a | b | *** | ** | d | a | d | * | 6 |
| Burkhart, C. S., et al., 2010 | b | a | a | b | *** | ** | d | a | d | * | 6 |
| Cape, E., et al., 2014 | a | a | a | b | *** | None | d | b | d | None | 3 |
| Casey, C. P., et al., 2020 | b | a | a | b | *** | None | d | a | b | ** | 5 |
| Cereghetti, C., et al., 2017 | a | a | a | b | *** | ** | d | a | d | * | 6 |
| Cerejeira, J., et al., 2013 | a | a | a | a | **** | None | d | b | d | None | 4 |
| Cerejeira, J., et al., 2012 | b | a | a | a | **** | None | d | b | d | None | 4 |
| CheheiliSobbi, S. et al., 2021 | b | a | a | a | **** | None | d | a | d | * | 5 |
| Chen, J., et al., 2022 | b | a | a | a | **** | ** | d | a | d | * | 7 |
| Chen, Y., et al., 2019 | b | a | a | a | **** | ** | d | a | a | ** | 8 |
| Chen, X. W., et al., 2014 | b | a | a | b | *** | * | d | b | a | * | 5 |
| Chu, C. S., et al., 2016 | a | a | a | a | **** | ** | d | a | d | * | 7 |
| Chung, K. S., et al., 2015 | a | a | a | b | *** | ** | d | b | b | * | 6 |
| Cizginer, S., et al., 2017 | b | a | a | a | **** | ** | d | a | d | * | 7 |
| Colkesen, Y., et al., 2013 | b | a | a | a | **** | ** | d | a | d | * | 7 |
| de Castro, S., 2014 | a | a | a | b | *** | ** | d | a | d | * | 6 |
| de Rooij, S. E., et al., 2007 | a | a | a | b | *** | ** | d | a | d | * | 6 |
| Deiner, S., et al., 2014 | b | a | a | b | *** | ** | d | b | d | None | 5 |
| Dittrich, T., et al., 2016 | a | a | a | a | **** | ** | b | b | a | * | 8 |
| Egberts, A. and Mattace-Rraso, F. U. S., 2017 | a | a | a | b | *** | None | b | a | d | ** | 5 |
| Egberts, A., et al., 2015 | b | a | a | b | *** | None | d | a | d | * | 4 |
| Erikson, K., et al., 2019 | a | a | a | b | *** | None | d | a | d | * | 4 |
| Eshmawey, M., et al., 2019 | a | a | a | b | *** | None | d | a | d | * | 4 |
| Fassbender, K., et al., 1994 | a | a | a | b | *** | None | d | b | d | None | 3 |
| Feng, Q., et al., 2019 | b | a | a | b | *** | None | d | a | d | * | 4 |
| Fernandez-Jimenez, E., et al., 2021 | a | a | a | b | *** | ** | b | b | d | * | 5 |
| Forget, M. F., et al.,2021 | a | a | a | b | *** | ** | b | a | a | ** | 7 |
| Foroughan, M., et al., 2016 | a | a | a | b | *** | None | d | a | d | * | 4 |
| Girard, T. D., et al., 2012 | b | a | a | b | *** | ** | d | b | a | * | 6 |
| Guenther, U., et al., 2013 | a | a | a | b | *** | ** | d | a | d | * | 6 |
| Guldolf, K., et al., 2021 | a | a | a | b | *** | ** | b | a | a | ** | 7 |
| Guo, Y., et al., 2016 | b | a | a | a | *** | ** | b | b | a | ** | 7 |
| Hall, R. J., et al., 2016 | b | a | a | b | *** | ** | d | a | a | ** | 7 |
| Hasegawa, T., et al. ,2015 | a | a | a | b | *** | ** | b | a | a | *** | 8 |
| Hatta, K., et al., 2014 | b | a | a | a | **** | ** | d | a | d | * | 7 |
| He, R., et al., 2020 | b | a | a | a | **** | ** | d | a | d | * | 7 |
| Hindiskere, S., et al., 2020 | b | a | a | a | **** | ** | b | a | a | ** | 8 |
| Jiang, X., et al., 2020 | a | a | a | b | *** | ** | b | a | a | *** | 8 |
| John, M., et al., 2017 | b | a | a | a | **** | None | a | b | b | ** | 6 |
| Jorge-Ripper, C., et al., 2017 | a | a | a | b | *** | ** | b | a | a | *** | 8 |
| Katsumi, Y., et al., 2020 | b | a | a | b | *** | None | d | a | d | * | 4 |
| Kazmierski, J., et al., 2022 | a | a | a | a | **** | ** | d | a | b | ** | 8 |
| Kazmierski, J., et al. 2014 | a | a | a | a | **** | ** | d | a | d | * | 7 |
| Kazmierski, J., et al. 2013 | a | a | a | a | **** | ** | d | a | d | * | 7 |
| Kazmierski, J., et al. 2014 | a | a | a | a | **** | ** | d | a | d | * | 7 |
| Khan, S. H., et al., 2022 | b | a | a | b | *** | ** | d | a | d | * | 6 |
| Kim, M. Y., et al., 2016 | b | a | a | a | **** | ** | d | a | d | * | 7 |
| Kinoshita, H., et al., 2021 | b | a | a | b | *** | ** | a | a | d | ** | 7 |
| Knaak, C., et al., 2019 | a | a | a | b | *** | ** | d | a | d | * | 6 |
| Kotfis, K., et al., 2019a | a | a | a | b | *** | ** | b | a | a | *** | 8 |
| Kotfis, K., et al., 2019b | b | a | a | a | **** | ** | b | a | a | *** | 9 |
| Kotfis, K., et al., 2019c | a | a | a | b | *** | ** | b | a | a | *** | 8 |
| Kowalska, K., et al., 2018 | a | a | a | a | **** | ** | b | a | d | * | 7 |
| Kozak, H. H., et al., 2017 | b | a | a | b | *** | None | d | a | d | * | 4 |
| Kupiec, A., et al., 2020 | b | a | a | b | *** | ** | d | b | d | None | 5 |
| Lammers-Lietz, F., et al., 2022 | b | a | a | b | *** | ** | d | a | d | * | 6 |
| Lechowicz, K., et al., 2021 | b | a | a | b | *** | * | d | a | d | * | 5 |
| Lee, H. J., et al., 2011 | b | a | a | a | **** | None | d | a | d | * | 5 |
| Lemstra, A. W., et al., 2008 | a | b | a | a | *** | None | a | a | c | ** | 5 |
| Li, X., et al., 2022 | b | a | a | a | **** | ** | d | b | d | None | 6 |
| Li, G., et al., 2017 | b | a | a | a | **** | ** | a | b | d | ** | 7 |
| Li, Q-H., et al., 2019 | b | a | a | a | **** | ** | d | a | d | * | 7 |
| Liu, P., et al., 2013 | b | a | a | b | *** | ** | a | a | a | *** | 8 |
| Lu, G. W., et al., 2020 | b | a | a | b | *** | ** | d | a | d | * | 6 |
| Lv, X. C., et al., 2021 | b | a | a | b | *** | None | a | a | d | ** | 5 |
| Ma, X., et al., 2022 | b | a | a | a | **** | ** | d | b | d | None | 6 |
| Macdonald, A., et al., 2007 | a | a | a | b | *** | ** | d | a | d | * | 6 |
| Mao, M., et al., 2022 | a | a | a | a | **** | ** | d | b | d | None | 6 |
| McIntosh, T. K., et al., 1985 | b | a | a | b | *** | None | d | a | d | * | 4 |
| McManus, J., et al., 2009 | a | a | a | b | *** | ** | d | a | d | ** | 7 |
| Miao, S., et al., 2018 | b | a | a | b | *** | ** | a | a | a | *** | 8 |
| Mietani, K., et al., 2022a | b | a | a | b | *** | ** | d | b | d | None | 5 |
| Mietani, K., et al., 2022b | b | a | a | b | *** | ** | d | b | d | None | 5 |
| Morandi, A., et al., 2011 | a | a | a | b | **** | ** | d | a | d | * | 7 |
| Mu, D. L., et al., 2010 | a | a | a | b | *** | ** | a | a | d | ** | 8 |
| Nagase, M., et al., 2012 | a | a | a | b | *** | ** | b | a | a | *** | 8 |
| Nakamura, J., et al., 2001 | a | a | a | b | *** | None | d | b | d | None | 3 |
| Neerland, B. E., et al., 2016 | a | a | a | b | *** | None | d | a | b | *** | 6 |
| Nydahl, P., et al., 2017 | a | a | a | b | *** | ** | d | a | d | ** | 7 |
| Osse, R. J., et al., 2012 | a | a | a | a | **** | ** | d | a | d | * | 7 |
| Oyama, T., et al., 2022 | a | a | a | b | *** | ** | b | b | a | ** | 7 |
| Page, V. J., et al., 2014 | a | a | a | b | *** | None | d | a | a | ** | 5 |
| Pasqui, E., et al., 2022 | a | a | a | b | *** | ** | b | a | a | *** | 8 |
| Peng, J., et al., 2019 | b | a | a | b | *** | ** | a | a | d | ** | 7 |
| Pfister, D., et al., 2008 | b | a | a | b | *** | None | d | b | a | * | 4 |
| Pol, R. A., et al., 2014 | a | a | a | b | *** | ** | d | a | d | * | 6 |
| Pol, R. A., et al., 2011 | a | a | a | b | *** | ** | d | a | d | * | 6 |
| Ren, Q., et al., 2020 | b | a | a | b | *** | ** | b | b | d | * | 6 |
| Reznik, M. E., et al., 2022 | a | a | a | b | *** | ** | b | a | a | *** | 8 |
| Ritchie, C. W., et al., 2014 | a | a | a | b | *** | ** | d | a | d | * | 6 |
| Ritter, C., et al., 2014 | a | a | a | a | **** | ** | a | b | d | * | 7 |
| Sakaguchi, T., et al., 2018 | b | a | a | b | *** | ** | d | a | d | * | 6 |
| Seo, C. L., et al., 2021 | a | a | a | a | **** | None | d | a | a | ** | 6 |
| Shen, H., et al., 2016 | b | a | a | a | **** | None | d | a | d | ** | 6 |
| Shi, C., et al., 2010 | b | a | a | b | *** | ** | d | a | d | * | 6 |
| Shi, Q., et al., 2019 | a | a | a | b | *** | ** | d | a | d | * | 6 |
| Simons, K. S., et al., 2018 | a | a | a | a | **** | None | d | a | d | * | 5 |
| Skrede, K., et al., 2015 | b | a | a | a | **** | None | d | a | d | * | 5 |
| Skrobik, Y., et al., 2013 | a | a | a | b | *** | None | d | b | a | * | 4 |
| Slor, C. J., et al., 2019 | b | a | a | b | *** | ** | d | a | b | ** | 7 |
| Song, Y., et al., 2022 | a | a | a | a | **** | ** | d | a | d | * | 7 |
| Sun, L., et al., 2016 | b | a | a | b | *** | None | d | b | d | None | 3 |
| Tanabe, S., et al., 2020 | b | a | a | b | *** | None | d | a | b | ** | 5 |
| Theologou, S., et al., 2018 | a | a | a | a | **** | ** | d | b | d | None | 6 |
| Thisayakorn, P., et al., 2022 | a | a | a | b | *** | ** | d | b | d | None | 6 |
| Thisayakorn, P., et al., 2021 | b | a | a | b | **** | ** | d | a | d | * | 7 |
| Tsuruta, R., et al., 2010 | b | a | a | b | *** | ** | d | a | d | * | 5 |
| van den Boogaard, M., et al., 2011 | b | a | a | a | **** | ** | a | a | d | ** | 8 |
| van der Sluis, F. J., et al., 2017 | a | a | a | b | *** | ** | d | b | c | * | 5 |
| van Munster, B. C., et al., 2008 | a | a | a | b | *** | ** | d | a | d | * | 6 |
| van Munster, B. C., et al., 2010 | a | a | a | b | *** | ** | d | a | d | * | 6 |
| Vasunilashorn, S. M., et al., 2017 | b | a | a | a | **** | ** | d | a | d | * | 7 |
| Visser, L., et al., 2015 | a | a | a | b | *** | ** | d | a | d | * | 6 |
| Wang, C. G., at al., 2018 | b | a | a | b | *** | ** | d | a | d | * | 6 |
| Watts, G., et al., 2007 | b | a | a | a | **** | None | b | a | d | ** | 6 |
| Wilson, K., et al., 2005 | a | a | a | a | **** | ** | d | b | d | None | 6 |
| Winkelman, C., et al., 2018 | a | a | a | b | *** | None | a | a | a | *** | 6 |
| Wu, J. G., et al., 2022 | b | a | a | b | *** | None | d | a | d | * | 4 |
| Xiang, D., et al., 2017 | b | a | a | a | **** | ** | d | a | d | * | 7 |
| Xu, W-B., et al., 2019 | b | a | a | b | *** | None | d | a | d | * | 4 |
| Yang, J. S., et al., 2022 | a | a | a | a | **** | ** | b | b | d | None | 6 |
| Yen, T. E., et al., 2016 | b | a | a | a | **** | ** | d | b | d | None | 6 |
| Zhang, S., et al., 2022b | a | a | a | b | *** | ** | d | a | d | ** | 6 |
| Zhang, L., et al., 2022a | b | a | a | b | *** | ** | d | a | d | * | 6 |
| Zhang, W., et al., 2022c | a | a | a | a | **** | None | d | a | d | * | 5 |
| Zhang, Z. Y., et al 2016b | b | a | a | a | **** | None | d | a | d | * | 5 |
| Zhang, Z., et al., 2014 | a | a | a | a | **** | ** | d | a | d | * | 7 |
| Zhang, L. N., et al., 2016a | b | a | a | b | *** | None | d | a | d | * | 4 |
| Zhao, Y., et al., 2021 | a | a | a | a | **** | None | d | a | a | ** | 6 |

Table 12 Risk of Bias of Studies included in the Systematic Review measured with the Newcastle Ottawa Scale for Case-Control Studies. High risk 0-3, Moderate Risk 4-6, Low Risk 7-9

| **Author** | SELECTION | | | | | COMPARABILITY | EXPOSURE | | | | NOS Score |
| --- | --- | --- | --- | --- | --- | --- | --- | --- | --- | --- | --- |
|  | 1 | 2 | 3 | 4 | Overall |  | 1 | 2 | 3 | Overall |  |
| Brum, C., et al., 2015 | a | a | a | a | **** | ** | a | a | a | *** | 9 |
| Capri, M., et al., 2014 | a | b | a | a | *** | ** | a | a | a | *** | 8 |
| Dillon, S. T., et al., 2017 | a | b | a | a | *** | None | a | a | c | ** | 5 |
| McKay, T. B., et al., 2022 | a | b | a | a | *** | None | a | a | c | ** | 5 |
| Plaschke, K., et al., 2013 | a | b | a | a | *** | None | a | a | c | ** | 5 |
| Plaschke, K., et al., 2010 | a | b | a | a | *** | None | a | a | c | ** | 5 |
| Rudolph, J. L., et al., 2008 | a | b | a | a | *** | None | a | a | c | ** | 5 |
| Sanchez, J. C., et al., 2021 | a | b | a | a | *** | None | a | a | a | *** | 6 |
| Soler-Sanchis, A., et al, 2022 | a | a | a | a | **** | None | a | a | c | ** | 6 |
| van den Boogaard, M., et al., 2011 | a | b | a | a | *** | None | a | a | c | ** | 5 |
| Vasunilashorn, S. M., et al, 2019 | a | a | a | a | **** | ** | a | a | c | ** | 8 |
| Vasunilashorn, S. M., et al., 2022 | a | a | a | a | **** | ** | a | a | c | ** | 8 |
| Vasunilashorn, S. M., et al., 2015 | a | a | a | a | **** | ** | a | a | c | ** | 8 |
| Wanderlind, M. L. Z., et al., 2020 | a | a | a | a | **** | None | a | a | c | ** | 6 |
| Wang, B., et al., 2022 | a | a | a | a | **** | None | a | a | a | *** | 7 |


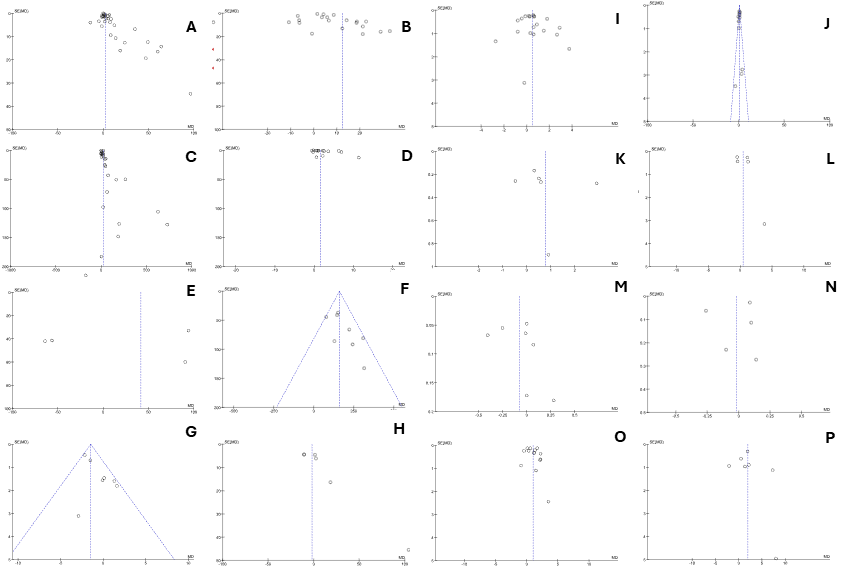


**Figure 33) Funnels plots of records included in meta-analyses A** CRP measured preceding delirium, **B** CRP measured during delirium, **C** IL-6 measured preceding delirium, **D** IL-6 measured during delirium, **E** Cortisol measured preceding delirium, **F** Cortisol measured during delirium, **G** IGF-1 measured preceding delirium, **H** PLR measured preceding delirium, **I** Leucocyte count measured preceding delirium, **J** Leucocyte count measured during delirium, **K** Neutrophil count measured preceding delirium, **L** Neutrophil count measured during delirium, **M** Lymphocyte count measured preceding delirium, **N** Lymphocyte count measured during delirium, **O** NLR measured preceding delirium, **P** NLR measured during delirium.

*IL-6 – Interleukin-6, PLR – Platelet to Lymphocyte Ratio, NLR – Neutrophil to lymphocyte ratio*

## References

Adamis, D., Coada, I., Eikelenboom, P., Chu, C.-S., Finn, K., Melvin, V., Williams, J., Meagher, D. J., & McCarthy, G. (2020). Delirium, insulin-like growth factor I, growth hormone in older inpatients. *World Journal of Psychiatry*, *10*(9), 212–222. https://doi.org/10.5498/wjp.v10.i9.212

Adamis, D., Lunn, M., Martin, F. C., Treloar, A., Gregson, N., Hamilton, G., & Macdonald, A. J. D. (2009). Cytokines and IGF-I in delirious and non-delirious acutely ill older medical inpatients. *Age and Ageing*, *38*(3), 251–326. https://dx.doi.org/10.1093/ageing/afp014

Adamis, D., Treloar, A., Martin, F. C., Gregson, N., Hamilton, G., & Macdonald, A. J. D. (2007). APOE and cytokines as biological markers for recovery of prevalent delirium in elderly medical inpatients. *International Journal of Geriatric Psychiatry*, *22*(7), 688–694.

Alexander, S. A., Ren, D., Gunn, S. R., Kochanek, P. M., Tate, J., Ikonomovic, M., & Conley, Y. P. (2014). Interleukin 6 and apolipoprotein E as predictors of acute brain dysfunction and survival in critical care patients. *American Journal of Critical Care : An Official Publication, American Association of Critical-Care Nurses*, *23*(1), 49–57. https://dx.doi.org/10.4037/ajcc2014578

Alvarez-Perez F.J. (2017). Prevalence and Risk Factors for Delirium in Acute Stroke Patients. A Retrospective 5-Years Clinical Series. *Journal of Stroke and Cerebrovascular Diseases*, *26*(3), 567–573. http://dx.doi.org/10.1016/j.jstrokecerebrovasdis.2016.11.120

Avila-Funes, J. A., Ledesma-Heyer, J. P., Navarrete-Reyes, A. P., Chavira-Ramírez, R., Boeck-Quirasco, L., & Aguilar-Navarro, S. (2015). Association between high serum estradiol levels and delirium among hospitalized elderly women. *Revista De Investigacion Clinica; Organo Del Hospital De Enfermedades De La Nutricion*, *67*(1), 20–24.

Ballweg, T., White, M., Parker, M., Casey, C., Bo, A., Farahbakhsh, Z., Kayser, A., Blair, A., Lindroth, H., Pearce, R. A., Blennow, K., Zetterberg, H., Lennertz, R., & Sanders, R. D. (2021). Association between plasma tau and postoperative delirium incidence and severity: A prospective observational study. *BJA: British Journal of Anaesthesia*, *126*(2), 458–466. https://doi.org/10.1016/j.bja.2020.08.061

Baranyi, A. (2014). The impact of soluble interleukin-2 receptor as a biomarker of delirium. *Psychosomatics*, *55*(1), 51–60. http://dx.doi.org/10.1016/j.psym.2013.06.004

Beloosesky, Y., Grinblat, J., Pirotsky, A., Weiss, A., & Hendel, D. (2004). Different C-reactive protein kinetics in post-operative hip-fractured geriatric patients with and without complications. *Gerontology*, *50*(4), 216–222.

Beloosesky, Y., Hendel, D., Weiss, A., Hershkovitz, A., Grinblat, J., Pirotsky, A., & Barak, V. (2007). Cytokines and C-reactive protein production in hip-fracture-operated elderly patients. *The Journals of Gerontology. Series A, Biological Sciences and Medical Sciences*, *62*(4), 420–426.

Bisschop, P. H., de Rooij, S. E., Zwinderman, A. H., van Oosten, H. E., & van Munster, B. C. (2011). Cortisol, insulin, and glucose and the risk of delirium in older adults with hip fracture. *Journal of the American Geriatrics Society*, *59*(9), 1692–1696. https://doi.org/10.1111/j.1532-5415.2011.03575.x

Brattinga, B., Plas, M., Spikman, J. M., Rutgers, A., de Haan, J. J., Absalom, A. R., van der Wal-Huisman, H., de Bock, G. H., & van Leeuwen, B. L. (2022). The association between the inflammatory response following surgery and post-operative delirium in older oncological patients: A prospective cohort study. *Age and Ageing*, *51*(2), afab237. https://doi.org/10.1093/ageing/afab237

Brum, C., Stertz, L., Borba, E., Rumi, D., Kapczinski, F., Camozzato, A., Brum, C., Stertz, L., Borba, E., Rumi, D., Kapczinski, F., & Camozzato, A. (2015). Association of serum brain-derived neurotrophic factor (BDNF) and tumor necrosis factor-alpha (TNF-α) with diagnosis of delirium in oncology inpatients. *Revista Brasileira de Psiquiatria*, *37*(3), 197–202. https://doi.org/10.1590/1516-4446-2014-1450

Burkhart C.S., Dell-Kuster S., Gamberini M., Moeckli A., Grapow M., Filipovic M., Seeberger M.D., Monsch A.U., & Strebel S.P. (2010). Modifiable and nonmodifiable risk factors for postoperative delirium after cardiac surgery with cardiopulmonary bypass. *Journal of Cardiothoracic and Vascular Anesthesia*, *24*(4), 555–559. http://dx.doi.org/10.1053/j.jvca.2010.01.003

Cape, E., Hall, R. J., van Munster, B. C., de Vries, A., Howie, S. E. M., Pearson, A., Middleton, S. D., Gillies, F., Armstrong, I. R., White, T. O., Cunningham, C., de Rooij, S. E., & MacLullich, A. M. J. (2014). Cerebrospinal fluid markers of neuroinflammation in delirium: A role for interleukin-1beta in delirium after hip fracture. *Journal of Psychosomatic Research*, *77*(3), 219–225. https://dx.doi.org/10.1016/j.jpsychores.2014.06.014

Capri, M., Yani, S. L., Chattat, R., Fortuna, D., Bucci, L., Lanzarini, C., Morsiani, C., Catena, F., Ansaloni, L., Adversi, M., Melotti, M. R., Di Nino, G., & Franceschi, C. (2014). Pre-Operative, High-IL-6 Blood Level is a Risk Factor of Post-Operative Delirium Onset in Old Patients. *Frontiers in Endocrinology*, *5*, 173. https://doi.org/10.3389/fendo.2014.00173

Casey, C. P., Lindroth, H., Mohanty, R., Farahbakhsh, Z., Ballweg, T., Twadell, S., Miller, S., Krause, B., Prabhakaran, V., Blennow, K., Zetterberg, H., & Sanders, R. D. (2020). Postoperative delirium is associated with increased plasma neurofilament light. *Brain: A Journal of Neurology*, *143*(1), 47–54. https://doi.org/10.1093/brain/awz354

Cereghetti C., Siegemund M., Schaedelin S., Fassl J., Seeberger M.D., Eckstein F.S., & Steiner L.A. (2017). Independent Predictors of the Duration and Overall Burden of Postoperative Delirium After Cardiac Surgery in Adults: An Observational Cohort Study. *Journal of Cardiothoracic and Vascular Anesthesia*, *31*(6), 1966–1973. http://dx.doi.org/10.1053/j.jvca.2017.03.042

Cerejeira, J., Batista, P., Nogueira, V., Vaz-Serra, A., & Mukaetova-Ladinska, E. B. (2013). The stress response to surgery and postoperative delirium: Evidence of hypothalamic-pituitary-adrenal axis hyperresponsiveness and decreased suppression of the GH/IGF-1 Axis. *Journal of Geriatric Psychiatry and Neurology*, *26*(3), 185–194. https://dx.doi.org/10.1177/0891988713495449

Cerejeira, J., Nogueira, V., Luís, P., Vaz-Serra, A., & Mukaetova-Ladinska, E. B. (2012). The Cholinergic System and Inflammation: Common Pathways in Delirium Pathophysiology. *Journal of the American Geriatrics Society*, *60*(4), 669–675. https://doi.org/10.1111/j.1532-5415.2011.03883.x

CheheiliSobbi, S., Peters van Ton, A. M., Wesselink, E. M., Looije, M. F., Gerretsen, J., Morshuis, W. J., Slooter, A. J. C., Abdo, W. F., Pickkers, P., & van den Boogaard, M. (2021). Case–control study on the interplay between immunoparalysis and delirium after cardiac surgery. *Journal of Cardiothoracic Surgery*, *16*(1), 239. https://doi.org/10.1186/s13019-021-01627-3

Chen, J., Ji, X., & Xing, H. (2022). Risk factors and a nomogram model for postoperative delirium in elderly gastric cancer patients after laparoscopic gastrectomy. *World Journal of Surgical Oncology*, *20*(1), 319. https://doi.org/10.1186/s12957-022-02793-x

Chen, X.-W., Shi, J.-W., Yang, P.-S., & Wu, Z.-Q. (2014). Preoperative plasma leptin levels predict delirium in elderly patients after hip fracture surgery. *Peptides*, *57*, 31–35. https://dx.doi.org/10.1016/j.peptides.2014.04.016

Chen, Y., Lu, S., Wu, Y., Shen, Y., Zhao, H., Ding, S., Feng, X., Sun, L., Tao, X., Li, J., Ma, R., Liu, W., Wu, F., & Feng, Y. (2019). Change in Serum Level of Interleukin 6 and Delirium After Coronary Artery Bypass Graft. *American Journal of Critical Care*, *28*(6), 462–470. https://doi.org/10.4037/ajcc2019976

Chu, C.-S., Liang, C.-K., Chou, M.-Y., Lin, Y.-T., Hsu, C.-J., Chu, C.-L., & Chou, P.-H. (2016). Lack of Association between Pre-Operative Insulin-Like Growth Factor-1 and the Risk of Post-Operative Delirium in Elderly Chinese Patients. *Psychiatry Investigation*, *13*(3), 327–332. https://doi.org/10.4306/pi.2016.13.3.327

Chung K.S., Lee J.K., & Park J.S. (2015). Risk factors of delirium in patients undergoing total knee arthroplasty. *Archives of Gerontology and Geriatrics*, *60*(3), 443–447. http://dx.doi.org/10.1016/j.archger.2015.01.021

Cizginer S., Marcantonio E., Vasunilashorn S., Pascual-Leone A., Shafi M., Schmitt E.M., Inouye S.K., & Richard N.; ORCID: http://orcid.org/0000-0002-1049-218X Jones R.N. AO - Jones. (2017). The Cognitive Reserve Model in the Development of Delirium: The Successful Aging after Elective Surgery Study. *Journal of Geriatric Psychiatry and Neurology*, *30*(6), 337–345. http://dx.doi.org/10.1177/0891988717732152

Colkesen, Y., Giray, S., Ozenli, Y., Sezgin, N., & Coskun, I. (2013). Relation of serum cortisol to delirium occurring after acute coronary syndromes. *The American Journal of Emergency Medicine*, *31*(1), 161–165. https://doi.org/10.1016/j.ajem.2012.07.001

De Castro, SM. m., Unlu, C., Tuynman, J. B., Honig, A., Van Wagensveld, B. A., & Steller, E. P. (2014). Incidence and risk factors of delirium in the elderly general surgical patient. *American Journal of Surgery*, *208*(1), 26–32. http://dx.doi.org/10.1016/j.amjsurg.2013.12.029

de Rooij, S. E., van Munster, B. C., Korevaar, J. C., & Levi, M. (2007). Cytokines and acute phase response in delirium. *Journal of Psychosomatic Research*, *62*(5), 521–525.

Deiner, S., Lin, H.-M., Bodansky, D., Silverstein, J., & Sano, M. (2014). Do stress markers and anesthetic technique predict delirium in the elderly? *Dementia and Geriatric Cognitive Disorders*, *38*(5–6), 366–374. https://doi.org/10.1159/000363762

Dillon, S. T., Vasunilashorn, S. M., Ngo, L., Otu, H. H., Inouye, S. K., Jones, R. N., Alsop, D. C., Kuchel, G. A., Metzger, E. D., Arnold, S. E., Marcantonio, E. R., & Libermann, T. A. (2017). Higher C-Reactive Protein Levels Predict Postoperative Delirium in Older Patients Undergoing Major Elective Surgery: A Longitudinal Nested Case-Control Study. *Biological Psychiatry*, *81*(2), 145–153. https://dx.doi.org/10.1016/j.biopsych.2016.03.2098

Dittrich T., Tschudin-Sutter S., Widmer A.F., Ruegg S., & Marsch S. (2016). Risk factors for new-onset delirium in patients with bloodstream infections: Independent and quantitative effect of catheters and drainages-a four-year cohort study. *Annals of Intensive Care*, *6*(1), 104. http://dx.doi.org/10.1186/s13613-016-0205-x

Egberts, A., & Mattace-Raso, F. U. (2017). Increased neutrophil-lymphocyte ratio in delirium: A pilot study. *Clinical Interventions in Aging*, *12*, 1115–1121. https://dx.doi.org/10.2147/CIA.S137182

Egberts, A., Wijnbeld, E. H. A., Fekkes, D., van der Ploeg, M. A., Ziere, G., Hooijkaas, H., van der Cammen, T. J. M., & Mattace-Raso, F. U. S. (2015). Neopterin: A potential biomarker for delirium in elderly patients. *Dementia and Geriatric Cognitive Disorders*, *39*(1–2), 116–124. https://dx.doi.org/10.1159/000366410

Erikson, K., Ala-Kokko, T. I., Koskenkari, J., Liisanantti, J. H., Kamakura, R., Herzig, K. H., & Syrjälä, H. (2019). Elevated serum S-100β in patients with septic shock is associated with delirium. *Acta Anaesthesiologica Scandinavica*, *63*(1), 69–73. https://doi.org/10.1111/aas.13228

Eshmawey, M., Arlt, S., Ledschbor-Frahnert, C., Guenther, U., & Popp, J. (2019). Preoperative Depression and Plasma Cortisol Levels as Predictors of Delirium after Cardiac Surgery. *Dementia and Geriatric Cognitive Disorders*, *48*(3–4), 207–214. https://doi.org/10.1159/000505574

Fassbender, K., Schmidt, R., Mössner, R., Daffertshofer, M., & Hennerici, M. (1994). Pattern of activation of the hypothalamic-pituitary-adrenal axis in acute stroke. Relation to acute confusional state, extent of brain damage, and clinical outcome. *Stroke*, *25*(6), 1105–1108. https://doi.org/10.1161/01.str.25.6.1105

Feng, Q., Ai, Y.-H., Gong, H., Wu, L., Ai, M.-L., Deng, S.-Y., Huang, L., Peng, Q.-Y., & Zhang, L.-N. (2019). Characterization of Sepsis and Sepsis-Associated Encephalopathy. *Journal of Intensive Care Medicine*, *34*(11–12), 938–945. https://doi.org/10.1177/0885066617719750

Fernández-Jiménez, E., Muñoz-Sanjose, A., Mediavilla, R., Martínez-Alés, G., Louzao, I. I., Andreo, J., Cebolla, S., Bravo-Ortiz, M.-F., & Bayón, C. (2021). Prospective Analysis Between Neutrophil-to-Lymphocyte Ratio on Admission and Development of Delirium Among Older Hospitalized Patients With COVID-19. *Frontiers in Aging Neuroscience*, *13*, 764334. https://doi.org/10.3389/fnagi.2021.764334

Forget, M.-F., Del Degan, S., Leblanc, J., Tannous, R., Desjardins, M., Durand, M., Vu, T. T. M., Nguyen, Q. D., & Desmarais, P. (2021). Delirium and Inflammation in Older Adults Hospitalized for COVID-19: A Cohort Study. *Clinical Interventions in Aging*, *16*, 1223–1230. https://doi.org/10.2147/CIA.S315405

Foroughan M., Delbari A., Said S.E., AkbariKamrani A.A., & Rashedi V. (2016). Risk factors and clinical aspects of delirium in elderly hospitalized patients in Iran. *Aging Clinical and Experimental Research*, *28*(2), 313–319. http://dx.doi.org/10.1007/s40520-015-0400-x

Girard, T. D., Ware, L. B., Bernard, G. R., Pandharipande, P. P., Thompson, J. L., Shintani, A. K., Jackson, J. C., Dittus, R. S., & Ely, E. W. (2012). Associations of markers of inflammation and coagulation with delirium during critical illness. *Intensive Care Medicine*, *38*(12), 1965–1973. https://dx.doi.org/10.1007/s00134-012-2678-x

Guenther, U., Theuerkauf, N., Frommann, I., Brimmers, K., Malik, R., Stori, S., Scheidemann, M., Putensen, C., & Popp, J. (2013). Predisposing and Precipitating Factors of Delirium After Cardiac Surgery: A Prospective Observational Cohort Study. *Annals of Surgery*, *257*(6), 1160–1167. https://doi.org/10.1097/SLA.0b013e318281b01c

Guldolf, K., Vandervorst, F., Gens, R., Ourtani, A., Scheinok, T., & De Raedt, S. (2021). Neutrophil-to-lymphocyte ratio predicts delirium after stroke. *Age and Ageing*, *50*(5), 1626–1632. https://doi.org/10.1093/ageing/afab133

Guo, Y., Jia, P., Zhang, J., Wang, X., Jiang, H., & Jiang, W. (2016). Prevalence and risk factors of postoperative delirium in elderly hip fracture patients. *The Journal of International Medical Research*, *44*(2), 317–327. https://dx.doi.org/10.1177/0300060515624936

Hall, R. J., Watne, L. O., Idland, A.-V., Raeder, J., Frihagen, F., MacLullich, A. M. J., Staff, A. C., Wyller, T. B., & Fekkes, D. (2016). Cerebrospinal fluid levels of neopterin are elevated in delirium after hip fracture. *Journal of Neuroinflammation*, *13*(1), 170. https://dx.doi.org/10.1186/s12974-016-0636-1

Hasegawa, T., Saito, I., Takeda, D., Iwata, E., Yonezawa, N., Kakei, Y., Sakakibara, A., Akashi, M., Minamikawa, T., & Komori, T. (2015). Risk factors associated with postoperative delirium after surgery for oral cancer. *Journal of Cranio-Maxillo-Facial Surgery : Official Publication of the European Association for Cranio-Maxillo-Facial Surgery*, *43*(7), 1094–1098. https://dx.doi.org/10.1016/j.jcms.2015.06.011

Hatta, K., Kishi, Y., Takeuchi, T., Wada, K., Odawara, T., Usui, C., Machida, Y., Nakamura, H., & Group, D.-J. (2014). The predictive value of a change in natural killer cell activity for delirium. *Progress in Neuro-Psychopharmacology & Biological Psychiatry*, *48*, 26–31. https://dx.doi.org/10.1016/j.pnpbp.2013.09.008

He, R., Wang, F., Shen, H., Zeng, Y., & LijuanZhang. (2020). Association between increased neutrophil-to-lymphocyte ratio and postoperative delirium in elderly patients with total hip arthroplasty for hip fracture. *BMC Psychiatry*, *20*(1), 496. https://doi.org/10.1186/s12888-020-02908-2

Hindiskere, S., Kim, H.-S., & Han, I. (2020). Postoperative delirium in patients undergoing surgery for bone metastases. *Medicine*, *99*(20), e20159. https://doi.org/10.1097/MD.0000000000020159

Jiang, X., Shen, Y., Fang, Q., Zhang, W., & Cheng, X. (2020). Platelet-to-lymphocyte ratio as a predictive index for delirium in critically ill patients. *Medicine*, *99*(43), e22884. https://doi.org/10.1097/MD.0000000000022884

John M., Ely E.W., Halfkann D., Schoen J., Sedemund-Adib B., Klotz S., Radtke F., & Stehr S. (2017). Acetylcholinesterase and butyrylcholinesterase in cardiosurgical patients with postoperative delirium. *Journal of Intensive Care*, *5*(1), 29. http://dx.doi.org/10.1186/s40560-017-0224-1

Kazmierski, J., Banys, A., Latek, J., Bourke, J., & Jaszewski, R. (2013). Cortisol levels and neuropsychiatric diagnosis as markers of postoperative delirium: A prospective cohort study. *Critical Care*, *17*(2), R38. https://doi.org/10.1186/cc12548

Kazmierski, J., Banys, A., Latek, J., Bourke, J., & Jaszewski, R. (2014). Raised IL-2 and TNF-alpha concentrations are associated with postoperative delirium in patients undergoing coronary-artery bypass graft surgery. *International Psychogeriatrics*, *26*(5), 845–855. https://dx.doi.org/10.1017/S1041610213002378

Kazmierski, J., Banys, A., Latek, J., Bourke, J., Jaszewski, R., Sobow, T., & Kloszewska, I. (2014). Mild cognitive impairment with associated inflammatory and cortisol alterations as independent risk factor for postoperative delirium. *Dementia and Geriatric Cognitive Disorders*, *38*(1–2), 65–78. https://dx.doi.org/10.1159/000357454

Kaźmierski, J., Miler, P., Pawlak, A., Jerczyńska, H., Nowakowska, K., Walkiewicz, G., Woźniak, K., Krejca, M., & Wilczyński, M. (n.d.). Increased postoperative myeloperoxidase concentration associated with low baseline antioxidant capacity as the risk factor of delirium after cardiac surgery. *Annals of Medicine*, *54*(1), 610–616. https://doi.org/10.1080/07853890.2022.2039405

Khan, S. H., Lindroth, H., Jawed, Y., Wang, S., Nasser, J., Seyffert, S., Naqvi, K., Perkins, A. J., Gao, S., Kesler, K., & Khan, B. (2022). Serum Biomarkers in Postoperative Delirium After Esophagectomy. *The Annals of Thoracic Surgery*, *113*(3), 1000–1007. https://doi.org/10.1016/j.athoracsur.2021.03.035

Kim, M. Y., Park, U. J., Kim, H. T., & Cho, W. H. (2016). DELirium Prediction Based on Hospital Information (Delphi) in General Surgery Patients. *Medicine*, *95*(12), e3072. https://doi.org/10.1097/MD.0000000000003072

Kinoshita, H., Saito, J., Takekawa, D., Ohyama, T., Kushikata, T., & Hirota, K. (2021). Availability of preoperative neutrophil-lymphocyte ratio to predict postoperative delirium after head and neck free-flap reconstruction: A retrospective study. *PLOS ONE*, *16*(7), e0254654. https://doi.org/10.1371/journal.pone.0254654

Knaak, C., Vorderwülbecke, G., Spies, C., Piper, S. K., Hadzidiakos, D., Borchers, F., Brockhaus, W.-R., Radtke, F. M., & Lachmann, G. (2019). C-reactive protein for risk prediction of post-operative delirium and post-operative neurocognitive disorder. *Acta Anaesthesiologica Scandinavica*, *63*(10), 1282–1289. https://doi.org/10.1111/aas.13441

Kotfis, K., Bott-Olejnik, M., Szylińska, A., Listewnik, M., & Rotter, I. (2019). Characteristics, Risk Factors And Outcome Of Early-Onset Delirium In Elderly Patients With First Ever Acute Ischemic Stroke—A Prospective Observational Cohort Study. *Clinical Interventions in Aging*, *14*, 1771–1782. https://doi.org/10.2147/CIA.S227755

Kotfis, K., Bott-Olejnik, M., Szylińska, A., & Rotter, I. (2019). Could Neutrophil-to-Lymphocyte Ratio (NLR) Serve as a Potential Marker for Delirium Prediction in Patients with Acute Ischemic Stroke? A Prospective Observational Study. *Journal of Clinical Medicine*, *8*(7), Article 7. https://doi.org/10.3390/jcm8071075

Kotfis, K., Ślozowska, J., Safranow, K., Szylińska, A., & Listewnik, M. (2019). The Practical Use of White Cell Inflammatory Biomarkers in Prediction of Postoperative Delirium after Cardiac Surgery. *Brain Sciences*, *9*(11), 308. https://doi.org/10.3390/brainsci9110308

Kowalska, K., Klimiec, E., Weglarczyk, K., Pera, J., Slowik, A., Siedlar, M., & Dziedzic, T. (2018). Reduced ex vivo release of pro-inflammatory cytokines and elevated plasma interleukin-6 are inflammatory signatures of post-stroke delirium. *Journal of Neuroinflammation*, *15*, 111. https://doi.org/10.1186/s12974-018-1156-y

Kozak, H. H., Uguz, F., Kilinc, I., Uca, A. U., Serhat Tokgoz, O., Akpinar, Z., & Ozer, N. (2017). Delirium in patients with acute ischemic stroke admitted to the non-intensive stroke unit: Incidence and association between clinical features and inflammatory markers. *Neurologia i Neurochirurgia Polska*, *51*(1), 38–44. https://dx.doi.org/10.1016/j.pjnns.2016.10.004

Kupiec, A., Adamik, B., Kozera, N., & Gozdzik, W. (2020). Elevated Procalcitonin as a Risk Factor for Postoperative Delirium in the Elderly after Cardiac Surgery—A Prospective Observational Study. *Journal of Clinical Medicine*, *9*(12), 3837. https://doi.org/10.3390/jcm9123837

Lammers-Lietz, F., Akyuz, L., Feinkohl, I., Lachmann, C., Pischon, T., Volk, H.-D., von Häfen, C., Yürek, F., Winterer, G., & Spies, C. D. (2022). Interleukin 8 in postoperative delirium—Preliminary findings from two studies. *Brain, Behavior, & Immunity - Health*, *20*, 100419. https://doi.org/10.1016/j.bbih.2022.100419

Lechowicz, K., Szylińska, A., Listewnik, M., Drożdżal, S., Tomska, N., Rotter, I., & Kotfis, K. (2021). Cardiac Delirium Index for Predicting the Occurrence of Postoperative Delirium in Adult Patients After Coronary Artery Bypass Grafting. *Clinical Interventions in Aging*, *16*, 487–495. https://doi.org/10.2147/CIA.S302526

Lee, H. J., Hwang, D. S., Wang, S. K., Chee, I. S., & S., S. (2011). Early assessment of delirium in elderly patients after hip surgery. *Psychiatry Investigation*, *8*(4), 340–347. http://dx.doi.org/10.4306/pi.2011.8.4.340

Lemstra, A. W., Kalisvaart, K. J., Vreeswijk, R., van Gool, W. A., & Eikelenboom, P. (2008). Pre-operative inflammatory markers and the risk of postoperative delirium in elderly patients. *International Journal of Geriatric Psychiatry*, *23*(9), 943–948. https://dx.doi.org/10.1002/gps.2015

Li, G., Lei, X., Ai, C., Li, T., & Chen, Z. (2017). Low plasma leptin level at admission predicts delirium in critically ill patients: A prospective cohort study. *Peptides*, *93*, 27–32. https://dx.doi.org/10.1016/j.peptides.2017.05.002

Li, Q.-H., Yu, L., Yu, Z.-W., Fan, X.-L., Yao, W.-X., Ji, C., Deng, F., Luo, X.-Z., & Sun, J.-L. (2019). Relation of postoperative serum S100A12 levels to delirium and cognitive dysfunction occurring after hip fracture surgery in elderly patients. *Brain and Behavior*, *9*(1), e01176. https://doi.org/10.1002/brb3.1176

Li, X., Wang, G., He, Y., Wang, Z., & Zhang, M. (2022). White-Cell Derived Inflammatory Biomarkers in Prediction of Postoperative Delirium in Elderly Patients Undergoing Surgery for Lower Limb Fracture Under Non-General Anaesthesia. *Clinical Interventions in Aging*, *17*, 383–392. https://doi.org/10.2147/CIA.S346954

Liu, P., Li, Y., Wang, X., Zou, X., Zhang, D., Wang, D., & Li, S. (2013). High serum interleukin-6 level is associated with increased risk of delirium in elderly patients after noncardiac surgery: A prospective cohort study. *Chinese Medical Journal*, *126*(19), 3621–3627.

Lu, G.-W., Chou, Y.-E., Jin, W.-L., & Su, X.-B. (2020). Usefulness of postoperative serum translocator protein as a predictive marker for delirium after breast cancer surgery in elderly women. *The Journal of International Medical Research*, *48*(6), 0300060520910044. https://doi.org/10.1177/0300060520910044

Lv, X.-C., Lin, Y., Wu, Q., Wang, L., Hou, Y., Dong, Y., & Chen, L. (2021). Plasma interleukin-6 is a potential predictive biomarker for postoperative delirium among acute type a aortic dissection patients treated with open surgical repair. *Journal of Cardiothoracic Surgery*, *16*, 146. https://doi.org/10.1186/s13019-021-01529-4

Ma, X., Mei, X., Tang, T., Wang, M., Wei, X., Zheng, H., Cao, J., Zheng, H., Cody, K., Xiong, L., Marcantonio, E. R., Xie, Z., & Shen, Y. (2022). Preoperative homocysteine modifies the association between postoperative C-reactive protein and postoperative delirium. *Frontiers in Aging Neuroscience*, *14*. https://www.frontiersin.org/articles/10.3389/fnagi.2022.963421

Macdonald, A., Adamis, D., Treloar, A., & Martin, F. (2007). C-reactive protein levels predict the incidence of delirium and recovery from it. *Age and Ageing*, *36*(2), 222–225.

Mao, M., Wang, L., Zhu, L., Wang, F., Ding, Y., Tong, J., Sun, J., Sun, Q., & Ji, M. (2022). Higher serum PGE2 is a predicative biomarker for postoperative delirium following elective orthopedic surgery in elderly patients. *BMC Geriatrics*, *22*(1), 685. https://doi.org/10.1186/s12877-022-03367-y

McIntosh, T. K., Bush, H. L., Yeston, N. S., Grasberger, R., Palter, M., Aun, F., & Egdahl, R. H. (1985). Beta-endorphin, cortisol and postoperative delirium: A preliminary report. *Psychoneuroendocrinology*, *10*(3), 303–313. https://doi.org/10.1016/0306-4530(85)90007-1

McKay, T. B., Rhee, J., Colon, K., Adelsberger, K., Turco, I., Mueller, A., Qu, J., & Akeju, O. (2022). Preliminary Study of Serum Biomarkers Associated With Delirium After Major Cardiac Surgery. *Journal of Cardiothoracic and Vascular Anesthesia*, *36*(1), 118–124. https://doi.org/10.1053/j.jvca.2021.05.002

McManus, J., Pathansali, R., Hassan, H., Ouldred, E., Cooper, D., Stewart, R., & MacDonald, A. (2009). The course of delirium in acute stroke. *Age and Ageing*, *38*(4), 385–389. http://dx.doi.org/10.1093/ageing/afp038

Miao, S., Shen, P., Zhang, Q., Wang, H., Shen, J., Wang, G., & Lv, D. (2018). Neopterin and Mini-Mental State Examination scores, two independent risk factors for postoperative delirium in elderly patients with open abdominal surgery. *Journal of Cancer Research and Therapeutics*, *0*(0), 0. https://doi.org/10.4103/0973-1482.192764

Mietani, K., Hasegawa-Moriyama, M., Yagi, K., Inoue, R., Ogata, T., Kurano, M., Shimojo, N., Seto, Y., Sumitani, M., & Uchida, K. (2022). Preoperative detection of serum phosphorylated neurofilament heavy chain subunit predicts postoperative delirium: A prospective observational study. *JOURNAL OF GERONTOLOGY AND GERIATRICS*, *70*, 169–177. https://doi.org/10.36150/2499-6564-N488

Mietani, K., Hasegawa-Moriyama, M., Yagi, K., Inoue, R., Ogata, T., Shimojo, N., Seto, Y., Uchida, K., & Sumitani, M. (2022). Elevation of serum plasminogen activator inhibitor-1 predicts postoperative delirium independent of neural damage: A sequential analysis. *Scientific Reports*, *12*(1), 17091. https://doi.org/10.1038/s41598-022-21682-7

Morandi, A., Gunther, M. L., Pandharipande, P. P., Jackson, J. C., Thompson, J. L., Shintani, A. K., Ely, E. W., & Girard, T. D. (2011). Insulin-Like Growth Factor-1 and Delirium in Critically Ill Mechanically Ventilated Patients: A Preliminary Investigation. *International Psychogeriatrics / IPA*, *23*(7), 1175–1181. https://doi.org/10.1017/S1041610210002486

Mu, D.-L., Wang, D.-X., Li, L.-H., Shan, G.-J., Li, J., Yu, Q.-J., & Shi, C.-X. (2010). High serum cortisol level is associated with increased risk of delirium after coronary artery bypass graft surgery: A prospective cohort study. *Critical Care (London, England)*, *14*(6), R238. https://doi.org/10.1186/cc9393

Nagase, M., Okamoto, Y., Tsuneto, S., Tanimukai, H., Matsuda, Y., Okishiro, N., Oono, Y., Tsugane, M., Takagi, T., & Uejima, E. (2012). A retrospective chart review of terminal patients with cancer with agitation and their risk factors. *Journal of Palliative Medicine*, *15*(11), 1185–1190. https://dx.doi.org/10.1089/jpm.2012.0114

Nakamura, J., Yoshimura, R., Okuno, T., Ueda, N., Hachida, M., Yasumoto, K., Egami, H., Maeda, H., Nishi, M., & Aoyagi, S. (2001). Association of plasma free-3-methoxy-4-hydroxyphenyl (ethylene)glycol, natural killer cell activity and delirium in postoperative patients. *International Clinical Psychopharmacology*, *16*(6), 339–343.

Neerland, B. E., Hall, R. J., Seljeflot, I., Frihagen, F., MacLullich, A. M. J., Raeder, J., Wyller, T. B., & Watne, L. O. (2016). Associations Between Delirium and Preoperative Cerebrospinal Fluid C-Reactive Protein, Interleukin-6, and Interleukin-6 Receptor in Individuals with Acute Hip Fracture. *Journal of the American Geriatrics Society*, *64*(7), 1456–1463. https://dx.doi.org/10.1111/jgs.14238

Nydahl, P., Bartoszek, G., Binder, A., Paschen, L., Margraf, N. G., Witt, K., & Ewers, A. (2017). Prevalence for delirium in stroke patients: A prospective controlled study. *Brain and Behavior*, *7*(8), e00748. http://dx.doi.org/10.1002/brb3.748

Osse, R. J., Fekkes, D., Tulen, J. H. M., Wierdsma, A. I., Bogers, A. J. J. C., van der Mast, R. C., & Hengeveld, M. W. (2012). High Preoperative Plasma Neopterin Predicts Delirium After Cardiac Surgery in Older Adults. *Journal of the American Geriatrics Society*, *60*(4), 661–668. https://doi.org/10.1111/j.1532-5415.2011.03885.x

Oyama, T., Kinoshita, H., Takekawa, D., Saito, J., Kushikata, T., & Hirota, K. (2022). Higher neutrophil-to-lymphocyte ratio, mean platelet volume, and platelet distribution width are associated with postoperative delirium in patients undergoing esophagectomy: A retrospective observational study. *Journal of Anesthesia*, *36*(1), 58–67. https://doi.org/10.1007/s00540-021-03007-6

Pasqui, E., de Donato, G., Brancaccio, B., Casilli, G., Ferrante, G., Cappelli, A., & Palasciano, G. (2022). The Predictive Role of Inflammatory Biochemical Markers in Post-Operative Delirium After Vascular Surgery Procedures. *Vascular Health and Risk Management*, *Volume 18*, 747–756. https://doi.org/10.2147/VHRM.S368194

Peng, J., Wu, G., Chen, J., & Chen, H. (2019). Preoperative C-Reactive Protein/Albumin Ratio, a Risk Factor for Postoperative Delirium in Elderly Patients After Total Joint Arthroplasty. *The Journal of Arthroplasty*, *34*(11), 2601–2605. https://doi.org/10.1016/j.arth.2019.06.042

Pfister, D., Siegemund, M., Dell-Kuster, S., Smielewski, P., Rüegg, S., Strebel, S. P., Marsch, S. C., Pargger, H., & Steiner, L. A. (2008). Cerebral perfusion in sepsis-associated delirium. *Critical Care*, *12*(3), R63. https://doi.org/10.1186/cc6891

Plaschke K. (2013). The role of interleukin-6 in postoperative delirium. *Proteomics Research Journal*, *4*(4), 361–368.

Plaschke, K., Fichtenkamm, P., Schramm, C., Hauth, S., Martin, E., Verch, M., Karck, M., & Kopitz, J. (2010). Early postoperative delirium after open-heart cardiac surgery is associated with decreased bispectral EEG and increased cortisol and interleukin-6. *Intensive Care Medicine*, *36*(12), 2081–2089. https://dx.doi.org/10.1007/s00134-010-2004-4

Pol, R. A., van Leeuwen, B. L., Izaks, G. J., Reijnen, M. M. P. J., Visser, L., Tielliu, I. F. J., & Zeebregts, C. J. (2014). C-reactive protein predicts postoperative delirium following vascular surgery. *Annals of Vascular Surgery*, *28*(8), 1923–1930. https://dx.doi.org/10.1016/j.avsg.2014.07.004

Pol R.A., Van Leeuwen B.L., Visser L., Izaks G.J., Van Den Dungen J.J.A.M., & Tielliu I.F.J. (2011). Standardised frailty indicator as predictor for postoperative delirium after vascular surgery: A prospective cohort study. *European Journal of Vascular and Endovascular Surgery*, *42*(6), 824–830. http://dx.doi.org/10.1016/j.ejvs.2011.07.006

Ren, Q., Wen, Y., Wang, J., Yuan, J., Chen, X., Thapa, Y., Qiang, M., & Xu, F. (2020). Elevated Level of Serum C-reactive Protein Predicts Postoperative Delirium among Patients Receiving Cervical or Lumbar Surgery. *BioMed Research International*, *2020*, 5480148. https://doi.org/10.1155/2020/5480148

Reznik, M. E., Kalagara, R., Moody, S., Drake, J., Margolis, S. A., Cizginer, S., Mahta, A., Rao, S. S., Stretz, C., Wendell, L. C., Thompson, B. B., Asaad, W. F., Furie, K. L., Jones, R. N., & Daiello, L. A. (2021). Common Biomarkers of Physiologic Stress and Associations with Delirium in Patients with Intracerebral Hemorrhage. *Journal of Critical Care*, *64*, 62–67. https://doi.org/10.1016/j.jcrc.2021.03.009

Ritchie, C. W., Newman, T. H., Leurent, B., & Sampson, E. L. (2014). The association between C-reactive protein and delirium in 710 acute elderly hospital admissions. *International Psychogeriatrics*, *26*(5), 717–724. https://dx.doi.org/10.1017/S1041610213002433

Ritter, C., Tomasi, C. D., Dal-Pizzol, F., Pinto, B., Dyson, A., de Miranda, A. S., Comim, C. M., Soares, M., Teixeira, A. L., Quevedo, J., & Singer, M. (2014). Inflammation biomarkers and delirium in critically ill patients. *Critical Care*, *18*(3), R106. https://doi.org/10.1186/cc13887

Rudolph, J. L., Salow, M. J., Angelini, M. C., & McGlinchey, R. E. (2008). The anticholinergic risk scale and anticholinergic adverse effects in older persons. *Archives of Internal Medicine*, *168*(5), 508–513.

Sakaguchi T., Watanabe M., Kawasaki C., Kuroda I., Abe H., Date M., Ueda Y., & Yasumura Y. (2018). A novel scoring system to predict delirium and its relationship with the clinical course in patients with acute decompensated heart failure. *Journal of Cardiology*, *71*(6), 564–569. http://dx.doi.org/10.1016/j.jjcc.2017.11.011

Sánchez, J. C., Ospina, J. P., & González, M. I. (2013). Association between leptin and delirium in elderly inpatients. *Neuropsychiatric Disease and Treatment*, *9*, 659–666. https://doi.org/10.2147/NDT.S44573

Shen, H., Shao, Y., Chen, J., & Guo, J. (2016). Insulin-Like Growth Factor-1, a Potential Predicative Biomarker for Postoperative Delirium Among Elderly Patients with Open Abdominal Surgery. *Current Pharmaceutical Design*, *22*(38), 5879–5883. https://doi.org/10.2174/1381612822666160813234311

Shi, C., Wang, D., Chen, K., & Gu, X. (2010). Incidence and risk factors of delirium in critically ill patients after non-cardiac surgery. *Chinese Medical Journal*, *123*(8), 993–999.

Shi, Q., Mu, X., Zhang, C., Wang, S., Hong, L., & Chen, X. (2019). Risk Factors for Postoperative Delirium in Type A Aortic Dissection Patients: A Retrospective Study. *Medical Science Monitor : International Medical Journal of Experimental and Clinical Research*, *25*, 3692–3699. https://doi.org/10.12659/MSM.913774

Simons, K. S., van den Boogaard, M., Hendriksen, E., Gerretsen, J., van der Hoeven, J. G., Pickkers, P., & de Jager, C. P. C. (2018). Temporal biomarker profiles and their association with ICU acquired delirium: A cohort study. *Critical Care*, *22*(1), 137. https://doi.org/10.1186/s13054-018-2054-5

Skrede, K., Wyller, T. B., Watne, L. O., Seljeflot, I., & Juliebo, V. (2015). Is there a role for monocyte chemoattractant protein-1 in delirium? Novel observations in elderly hip fracture patients. *BMC Research Notes*, *8*, 186. https://dx.doi.org/10.1186/s13104-015-1129-5

Slor, C. J., Witlox, J., Adamis, D., Jansen, R. W. M. M., Houdijk, A. P. J., van Gool, W. A., de Jonghe, J. F. M., & Eikelenboom, P. (2019). The trajectory of C-reactive protein serum levels in older hip fracture patients with postoperative delirium. *International Journal of Geriatric Psychiatry*, *34*(10), 1438–1446. https://doi.org/10.1002/gps.5139

Soler-Sanchis, A., Martínez-Arnau, F. M., Sánchez-Frutos, J., & Pérez-Ros, P. (2022). Challenges in the Detection of Clinically Useful Biomarkers for the Diagnosis of Delirium in Older People in the Emergency Department—A Case–Control Study. *Life*, *12*(8), 1127. https://doi.org/10.3390/life12081127

Song, Y., Luo, Y., Zhang, F., Ma, Y., Lou, J., Li, H., Liu, Y., Mi, W., & Cao, J. (2022). Systemic immune-inflammation index predicts postoperative delirium in elderly patients after surgery: A retrospective cohort study. *BMC Geriatrics*, *22*(1), 730. https://doi.org/10.1186/s12877-022-03418-4

Sun, L., Jia, P., Zhang, J., Zhang, X., Zhang, Y., Jiang, H., & Jiang, W. (2016). Production of inflammatory cytokines, cortisol, and Abeta1-40 in elderly oral cancer patients with postoperative delirium. *Neuropsychiatric Disease and Treatment*, *12*, 2789–2795. http://dx.doi.org/10.2147/NDT.S113077

Tanabe, S., Mohanty, R., Lindroth, H., Casey, C., Ballweg, T., Farahbakhsh, Z., Krause, B., Prabhakaran, V., Banks, M. I., & Sanders, R. D. (2020). Cohort study into the neural correlates of postoperative delirium: The role of connectivity and slow-wave activity. *BJA: British Journal of Anaesthesia*, *125*(1), 55–66. https://doi.org/10.1016/j.bja.2020.02.027

Theologou, S., Giakoumidakis, K., & Charitos, C. (2018). Perioperative predictors of delirium and incidence factors in adult patients post cardiac surgery. *Pragmatic and Observational Research*, *9*, 11–19. https://doi.org/10.2147/POR.S157909

Thisayakorn, P., Tangwongchai, S., Tantavisut, S., Thipakorn, Y., Sukhanonsawat, S., Wongwarawipat, T., Sirivichayakul, S., & Maes, M. (2021). Immune, Blood Cell, and Blood Gas Biomarkers of Delirium in Elderly Individuals with Hip Fracture Surgery. *Dementia and Geriatric Cognitive Disorders*, *50*(2), 161–169. https://doi.org/10.1159/000517510

Thisayakorn, P., Thipakorn, Y., Tantavisut, S., Sirivichayakul, S., & Maes, M. (2022). Delirium due to hip fracture is associated with activated immune-inflammatory pathways and a reduction in negative immunoregulatory mechanisms. *BMC Psychiatry*, *22*(1), 369. https://doi.org/10.1186/s12888-022-04021-y

Tsuruta, R., Nakahara, T., Miyauchi, T., Kutsuna, S., Ogino, Y., Yamamoto, T., Kaneko, T., Kawamura, Y., Kasaoka, S., & Maekawa, T. (2010). Prevalence and associated factors for delirium in critically ill patients at a Japanese intensive care unit. *General Hospital Psychiatry*, *32*(6), 607–611. https://dx.doi.org/10.1016/j.genhosppsych.2010.09.001

van den Boogaard, M., Kox, M., Quinn, K. L., van Achterberg, T., van der Hoeven, J. G., Schoonhoven, L., & Pickkers, P. (2011). Biomarkers associated with delirium in critically ill patients and their relation with long-term subjective cognitive dysfunction; indications for different pathways governing delirium in inflamed and noninflamed patients. *Critical Care*, *15*(6), R297. https://doi.org/10.1186/cc10598

van den Boogaard M., van Swelm R.P.L., Russel F.G.M., Heemskerk S., van der Hoeven J.G., & Masereeuw R. (2011). Urinary protein profiling in hyperactive delirium and non-delirium cardiac surgery ICU patients. *Proteome Science*, *9*, 13. http://dx.doi.org/10.1186/1477-5956-9-13

van der Sluis F.J., Buisman P.L., Meerdink M., aan de Stegge W.B., van Etten B., de Bock G.H., & van Leeuwen B.L. (2017). Risk factors for postoperative delirium after colorectal operation. *Surgery (United States)*, *161*(3), 704–711. http://dx.doi.org/10.1016/j.surg.2016.09.010

Van Munster, B. C., Korevaar, J. C., Zwinderman, A. H., Levi, M., Wiersinga, W. J., & De Rooij, S. E. (2008). Time-Course of Cytokines During Delirium in Elderly Patients with Hip Fractures. *Journal of the American Geriatrics Society*, *56*(9), 1704–1709. https://doi.org/10.1111/j.1532-5415.2008.01851.x

van Munster B.C., Bisschop P.H., Zwinderman A.H., Korevaar J.C., Endert E., W.J., W., H.E.V., O., & J.C., G. (2010). Cortisol, interleukins and S100B in delirium in the elderly. *Brain and Cognition*, *74*(1), 18–23. http://dx.doi.org/10.1016/j.bandc.2010.05.010

Vasunilashorn, S. M., Dillon, S. T., Chan, N. Y., Fong, T. G., Joseph, M., Tripp, B., Xie, Z., Ngo, L. H., Lee, C. G., Elias, J. A., Otu, H. H., Inouye, S. K., Marcantonio, E. R., & Libermann, T. A. (2021). Proteome-Wide Analysis Using SOMAscan Identifies and Validates Chitinase-3-Like Protein 1 as a Risk and Disease Marker of Delirium Among Older Adults Undergoing Major Elective Surgery. *The Journals of Gerontology Series A: Biological Sciences and Medical Sciences*, *77*(3), 484–493. https://doi.org/10.1093/gerona/glaa326

Vasunilashorn, S. M., Dillon, S. T., Inouye, S. K., Ngo, L. H., Fong, T. G., Jones, R. N., Travison, T. G., Schmitt, E. M., Alsop, D. C., Freedman, S. D., Arnold, S. E., Metzger, E. D., Libermann, T. A., & Marcantonio, E. R. (2017). High C-Reactive Protein Predicts Delirium Incidence, Duration, and Feature Severity After Major Noncardiac Surgery. *Journal of the American Geriatrics Society*, *65*(8), e109–e116. https://dx.doi.org/10.1111/jgs.14913

Vasunilashorn, S. M., Ngo, L. H., Chan, N. Y., Zhou, W., Dillon, S. T., Otu, H. H., Inouye, S. K., Wyrobnik, I., Kuchel, G. A., McElhaney, J. E., Xie, Z., Alsop, D. C., Jones, R. N., Libermann, T. A., & Marcantonio, E. R. (2019). Development of a Dynamic Multi-Protein Signature of Postoperative Delirium. *The Journals of Gerontology Series A: Biological Sciences and Medical Sciences*, *74*(2), 261–268. https://doi.org/10.1093/gerona/gly036

Vasunilashorn, S. M., Ngo, L., Inouye, S. K., Libermann, T. A., Jones, R. N., Alsop, D. C., Guess, J., Jastrzebski, S., McElhaney, J. E., Kuchel, G. A., & Marcantonio, E. R. (2015). Cytokines and Postoperative Delirium in Older Patients Undergoing Major Elective Surgery. *The Journals of Gerontology. Series A, Biological Sciences and Medical Sciences*, *70*(10), 1289–1295. https://dx.doi.org/10.1093/gerona/glv083

Visser L., Prent A., Van Der Laan M.J., Van Leeuwen B.L., Izaks G.J., & Zeebregts C.J. (2015). Predicting postoperative delirium after vascular surgical procedures. *Journal of Vascular Surgery*, *62*(1), 185–189. http://dx.doi.org/10.1016/j.jvs.2015.01.041

Wanderlind, M. L., Gonçalves, R., Tomasi, C. D., Dal-Pizzol, F., Link to external site, this link will open in a new window, Ritter, C., & Link to external site, this link will open in a new window. (2020). Association of neurogranin with delirium among critically ill patients. *Biomarkers in Medicine*, *14*(17), 1613–1617.

Wang, B., Yin, Z., Lin, Y., Deng, X., Liu, F., Tao, H., Dong, R., Lin, X., & Bi, Y. (2022). Correlation between microRNA-320 and postoperative delirium in patients undergoing tibial fracture internal fixation surgery. *BMC Anesthesiology*, *22*, 75. https://doi.org/10.1186/s12871-022-01612-w

Wang, C.-G., Qin, Y.-F., Wan, X., Song, L.-C., Li, Z.-J., & Li, H. (2018). Incidence and risk factors of postoperative delirium in the elderly patients with hip fracture. *Journal of Orthopaedic Surgery and Research*, *13*(1), 186. https://doi.org/10.1186/s13018-018-0897-8

Watts, G., Roberts, B. L., & Parsons, R. (2007). Delirium in the intensive care unit: Searching for causes and sources. *Critical Care and Resuscitation : Journal of the Australasian Academy of Critical Care Medicine*, *9*(1), 26–29.

Wilson, K., Broadhurst, C., Diver, M., Jackson, M., & Mottram, P. (2005). Plasma insulin growth factor—1 and incident delirium in older people. *International Journal of Geriatric Psychiatry*, *20*(2), 154–159. https://doi.org/10.1002/gps.1265

Xiang, D., Xing, H., Tai, H., & Xie, G. (2017). Preoperative C-Reactive Protein as a Risk Factor for Postoperative Delirium in Elderly Patients Undergoing Laparoscopic Surgery for Colon Carcinoma. *BioMed Research International*, *2017*, 5635640. https://dx.doi.org/10.1155/2017/5635640

Xu, W.-B., Hu, Q.-H., Wu, C.-N., Fan, Z.-K., & Song, Z.-F. (2019). Serum soluble fibrinogen-like protein 2 concentration predicts delirium after acute pancreatitis. *Brain and Behavior*, *9*(4), e01261. https://doi.org/10.1002/brb3.1261

Yang, J. S., Lee, J. J., Kwon, Y.-S., Kim, J.-H., & Sohn, J.-H. (2022). Preoperative Inflammatory Markers and the Risk of Postoperative Delirium in Patients Undergoing Lumbar Spinal Fusion Surgery. *Journal of Clinical Medicine*, *11*(14), Article 14. https://doi.org/10.3390/jcm11144085

Yen, T. E., Allen, J. C., Rivelli, S. K., Patterson, S. C., Metcalf, M. R., Flink, B. J., Mirrakhimov, A. E., Lagoo, S. A., Vail, T. P., Young, C. C., Moon, R. E., Trzepacz, P. T., & Kwatra, M. M. (2016). Association between Serum IGF-I levels and Postoperative Delirium in Elderly Subjects Undergoing Elective Knee Arthroplasty. *Scientific Reports*, *6*, 20736. https://doi.org/10.1038/srep20736

Zhang, L., Li, B., Bai, Y., Liu, X., & Chai, X. (2022). The C-reactive protein/albumin ratio predicts postoperative delirium in patients older than 60 years following total knee arthroplasty. *Frontiers in Surgery*, *9*, 814345. https://doi.org/10.3389/fsurg.2022.814345

Zhang L.-N., Wang X.-H., Wu L., Huang L., Zhao C.-G., & Peng Q.-Y. (2016). Diagnostic and predictive levels of calcium-binding protein a8 and tumor necrosis factor receptor-associated factor 6 in sepsis-associated encephalopathy: A prospective observational study. *Chinese Medical Journal*, *129*(14), 1674–1681. http://dx.doi.org/10.4103/0366-6999.185860

Zhang, S., Ji, M.-H., Ding, S., Wu, Y., Feng, X.-W., Tao, X.-J., Liu, W.-W., Ma, R.-Y., Wu, F.-Q., & Chen, Y.-L. (2022). Inclusion of interleukin-6 improved performance of postoperative delirium prediction for patients undergoing coronary artery bypass graft (POD-CABG): A derivation and validation study. *Journal of Cardiology*, *79*(5), 634–641. https://doi.org/10.1016/j.jjcc.2021.12.003

Zhang, W., Wang, R., Yuan, J., Li, B., Zhang, L., Wang, Y., Zhu, R., Zhang, J., & Huyan, T. (2022). The TLR4/NF-κB/MAGI-2 signaling pathway mediates postoperative delirium. *Aging (Albany NY)*, *14*(6), 2590–2606. https://doi.org/10.18632/aging.203955

Zhang, Z., Pan, L., Deng, H., Ni, H., & Xu, X. (2014). Prediction of delirium in critically ill patients with elevated C-reactive protein. *Journal of Critical Care*, *29*(1), 88–92. https://dx.doi.org/10.1016/j.jcrc.2013.09.002

Zhang Z.-Y., Gao D.-P., Sun X.-R., Zhang H., Hu J., Fang Z.-Y., & Yang J.-J. (2016). Impact of length of red blood cells transfusion on postoperative delirium in elderly patients undergoing hip fracture surgery: A cohort study. *Injury*, *47*(2), 408–412. http://dx.doi.org/10.1016/j.injury.2015.10.009

Zhao, Y., Yue, J., Lei, P., Lin, T., Peng, X., Xie, D., Gao, L., Shu, X., & Wu, C. (2021). Neutrophil-lymphocyte ratio as a predictor of delirium in older internal medicine patients: A prospective cohort study. *BMC Geriatrics*, *21*, 334. https://doi.org/10.1186/s12877-021-02284-w
